# Application of the eHealth Literacy Model in Digital Health Interventions: Scoping Review

**DOI:** 10.2196/23473

**Published:** 2021-06-03

**Authors:** Mariam El Benny, Tamar Kabakian-Khasholian, Fadi El-Jardali, Marco Bardus

**Affiliations:** 1 Department of Health Promotion and Community Health Faculty of Health Sciences American University of Beirut Beirut Lebanon; 2 Department of Health Management and Policy Faculty of Health Sciences American University of Beirut Beirut Lebanon

**Keywords:** eHealth literacy, digital health interventions, consumer health information, scoping review, mHealth, mobile phone

## Abstract

**Background:**

Digital health interventions (DHIs) are increasingly being adopted globally to address various public health issues. DHIs can be categorized according to four main types of technology: mobile based, web based, telehealth, and electronic health records. In 2006, Norman and Skinner introduced the *eHealth literacy model*, encompassing six domains of skills and abilities (basic, health, information, scientific, media, and computer) needed to effectively understand, process, and act on health-related information. Little is known about whether these domains are assessed or accounted for in DHIs.

**Objective:**

This study aims to explore how DHIs assess and evaluate the eHealth literacy model, describe which health conditions are addressed, and which technologies are used.

**Methods:**

We conducted a scoping review of the literature on DHIs, based on randomized controlled trial design and reporting the assessment of any domain of the eHealth literacy model. MEDLINE, CINAHL, Embase, and Cochrane Library were searched. A duplicate selection and data extraction process was performed; we charted the results according to the country of origin, health condition, technology used, and eHealth literacy domain.

**Results:**

We identified 131 unique DHIs conducted in 26 different countries between 2001 and 2020. Most DHIs were conducted in English-speaking countries (n=81, 61.8%), delivered via the web (n=68, 51.9%), and addressed issues related to noncommunicable diseases (n=57, 43.5%) or mental health (n=26, 19.8%). None of the interventions assessed all six domains of the eHealth literacy model. Most studies focused on the domain of health literacy (n=96, 73.2%), followed by digital (n=19, 14.5%), basic and media (n=4, 3%), and information and scientific literacy (n=1, 0.7%). Of the 131 studies, 7 (5.3%) studies covered both health and digital literacy.

**Conclusions:**

Although many selected DHIs assessed health or digital literacy, no studies comprehensively evaluated all domains of the eHealth literacy model; this evidence might be overlooking important factors that can mediate or moderate the effects of these interventions. Future DHIs should comprehensively assess the eHealth literacy model while developing or evaluating interventions to understand how and why interventions can be effective.

## Introduction

### Digital Health Interventions

In the last 20 years, digital health or eHealth has emerged as an important research field. At the intersection of medical informatics, public health, and business, eHealth refers to the use of “health services and information delivered or enhanced through the Internet and related technologies” [1]. Technologies such as web based, mobile based, telehealth, and electronic health records (EHRs) have become widely adopted in the so-called *digital health interventions* (DHIs). DHIs can be defined as “health services delivered electronically through formal or informal care. DHIs can range from electronic medical records used by providers to mobile health (mHealth) apps used by consumers” [2]. The World Health Organization has recently produced a classification of DHIs, identifying four main types: clients, health care providers, health systems, and data services [3]. On PubMed, as of August 3, 2020, the number of records mentioning *eHealth* or *DHIs* in their title or abstract has consistently increased over the past 20 years, starting from 65 in 2000 to 11,395 in 2019, reaching a total of 6720.

Some systematic reviews and meta-analyses have described the effectiveness of DHIs in addressing various public health problems, such as somatic diseases [4], or health literacy and health outcomes [5]. Nevertheless, it is still unclear what makes DHIs superior to nondigital interventions or what components of these interventions facilitate positive outcomes reported [6]. In addition, it is unclear whether DHIs are effective because of their content or the manner in which they are delivered. Regarding the content of interventions, some systematic reviews have focused on exploring the way people process and understand information available on the internet [7,8]. In fact, with so many resources and information available on the internet, patients and users enrolled in DHIs may face challenges in understanding and making sense of the information they receive. Some research has focused on problems related to the ability to process information derived from web-based sources or delivered through technologies.

### The eHealth Literacy Model

In 2006, Norman and Skinner [9] proposed a conceptual model that encompasses six different domains of literacy required to process information from technology sources: traditional literacy, health literacy, information literacy, scientific literacy, media literacy, and computer literacy. According to Norman and Skinner [9], traditional or basic functional literacy includes simple and primitive literacy skills, including the ability to read and understand text and the ability to speak and write in a certain language. Information literacy includes the ability to know how knowledge is structured and how information can be used in a certain way that informs other people. Media literacy is the capability to critique a media subject and place information in different contexts. Health literacy, coined in the 1970s, can be generally defined as “the degree to which individuals can obtain, process, understand, and communicate about health-related information needed to make informed health decisions” (as reported by Berkman et al [10]). According to Norman and Skinner [9], health literacy is the ability to perform basic reading and numerical tasks required to function in the health care environment; patients with adequate health literacy can read, understand, and act on health care information. More recent evolutions of the concept include a variety of competencies and skills, including knowledge, motivation, and competencies related to accessing, understanding, appraising, and applying health-related information in health care, disease prevention, and health promotion settings [11]. Several systematic reviews have analyzed the relationship between health literacy and a variety of health outcomes, indicating that a good level of health literacy is generally associated with positive health outcomes in various health domains, such as vaccination [12], noncommunicable diseases (NCDs) such as chronic kidney disease or coronary artery disease, heart failure [13-15], oral health [16], quality of life [17], and excess body weight [18]. Some other review evidence has shown how interventions promoting critical health literacy [19] could be very beneficial for the community [20] or among specific segments of the population, such as adolescents [21] or older adults [22].

Strictly related to the concept of eHealth is computer or technology literacy, which is the capability to use new technologies and software and the ability to access electronic health information [9]. Recent conceptualizations expand this domain to look at the ability to process information, to engage with patients’ own health, at the motivation and ability to engage with digital devices, at feeling safe and in control, at having access to health care and technological systems that work, and at meeting digital services that suit individuals’ needs [23]. Norman and Skinner [24] have developed a scale to assess eHealth literacy, called *eHealth literacy scale* (*eHEALS*), which has been one of the most adopted and cited, with 449 citations on the *Journal of Medical Internet Research* page and more than 1320 results on Google Scholar (as of August 3, 2020). The last domain of the eHealth literacy model, scientific literacy, involves the ability to allocate health-related findings in the right context by systematically understanding the “nature, aims, methods, applications, limitations, and politics” of building knowledge [9]. Several systematic reviews have analyzed the relationship between health literacy in mHealth apps and interventions [5,7,8,25,26], generally reporting positive associations among health literacy, digital literacy, and health outcomes. Other reviews have specifically examined how technology can affect health literacy in health programs [27-29].

According to the developers of the eHealth literacy model, the six domains can be grouped into two main categories: analytic (traditional, media, and information) and context-specific (health, scientific, and computer). The analytical category refers to a set of competencies that can be applied to a wide range of information sources, whereas context-specific categories include competencies that can only be applied to a specific problem in a specific context [9]. For example, the ability of a person living with type 2 diabetes to process information related to diabetes is different from their ability to process information related to vaccines, mental health, or other chronic conditions. Similarly, the ability to use a mobile phone to call someone does not necessarily translate into the ability to use a mobile app, navigate a website, or evaluate the information retrieved while searching on the internet.

### Related Work and Study Aims

Arguably, researchers developing DHIs should always take into account the domains of computer or technology literacy and health literacy, as these are potential pathways for more effective and equitable interventions [30]. Health literacy can be viewed as both an outcome and a mediator in interventions intended to improve health outcomes [31]. Technologies or delivery modes can also be seen as interacting or moderating factors [32], depending on the type of technology used to deliver an intervention on a specific health topic. DHIs can be developed to improve health literacy (outcome) or they can be developed to improve clinical outcomes in which one or more dimensions of the eHealth literacy model are considered as mediators or moderators of the effects of the intervention. Researchers developing DHIs could then assume that people enrolling in these interventions should have good levels of functional, scientific, media, and information literacy to understand how to write or read information they are exposed to.

However, to what extent are these assumptions tenable? In other words, is the eHealth literacy model purely conceptual or does it find a concrete application in DHIs? To the best of our knowledge, there are no systematic reviews that specifically discuss the application of the complete eHealth literacy model in DHIs. When we were developing the search strategies for this project, we searched for existing systematic reviews in PubMed and PROSPERO databases with the keyword *eHealth literacy* and identified only four systematic reviews [33-36]. However, all these reviews have focused on the domain of digital literacy, looking at specific health outcomes in specific segments of the population, such as people living with HIV [33], underserved populations in the United States [34], older adults [35], or college students [36]. Therefore, this scoping review aims to identify and describe DHIs that assess any domain of the eHealth literacy model and to identify which domains are assessed and evaluated the most. We considered DHIs that were developed to improve clinical outcomes or that were aimed at different literacies, according to the eHealth literacy model. In other words, we considered interventions that looked at eHealth literacy either as an outcome or as a mediator of intervention effects, as long as the domains of the eHealth literacy model were assessed.

## Methods

### Overview

We followed the scoping review framework by Arksey and O’Malley [37], which encompasses five stages: (1) identification of the initial research questions; (2) identification of relevant studies; (3) study selection; (4) charting the data; and (5) collating, summarizing, and reporting the results. The stages are described further in the following sections.

### Stage 1: Identifying the Research Question

The main review question, based on the eHealth literacy model, was “To what extent are DHIs assessing the 6 domains of the eHealth literacy model?” More specifically, we wanted to answer the following research questions: What domains of the eHealth literacy model (ie, computer, health, traditional, media, information, and science literacy) are assessed and reported in the literature? What health conditions have been investigated? What technologies are used?

### Stage 2: Identifying Relevant Studies

We searched four electronic databases that cover most of the medical and public health literature: MEDLINE, CINAHL, Embase, and Cochrane Library.

We used a predefined search strategy, encompassing keywords and medical subject headings to cover three main concepts: *eHealth literacy model*, *digital health*, and the *study design* for interventions. The *eHealth literacy model* concept entailed terms such as health literacy, literacy, computer literacy, information literacy, basic, functional, scientific, media, information, computer, health, eHealth, literacy, literate, illiteracy, and illiterate. The second concept, *digital health*, expanded on the above and entailed keywords, such as *telemedicine*, *internet*, *mobile*, *phone*, *digital*, *medium* or *media*, *mHealth*, *eHealth*, *telemedicine*, and *computer*, based on other systematic reviews recently conducted by one of the authors [6,38,39]. The third concept, that is, the research design, entailed a predefined set of keywords and operands that Cochrane has developed to identify randomized controlled trials (RCTs); this is because we wanted to identify the best level of evidence available [40]. The search strategy used for MEDLINE is provided in Multimedia Appendix 1. Database searches were completed, and references were retrieved on January 24, 2020.

In addition, we used the reference list of identified systematic reviews on the topic to identify other potentially relevant studies.

### Stage 3: Study Selection

We followed the Joanna Briggs’ Institute’s PCC (Population-Concept-Context) framework [41,42] to define our inclusion criteria, as it applies to scoping reviews. We included studies that discussed DHIs (concepts) and reported the assessment of at least one domain of the eHealth literacy model (context). In this context, we conceived the dimensions of the eHealth literacy model as either outcomes or mediators of DHIs. The assessment of the different types of literacy was considered a sufficient indicator for DHIs considering such dimensions as outcomes or mediators of intervention effects. We did not restrict the results to any population, with the idea of inductively categorizing the results according to health condition, hence defining the population of reference in the analytical phase.

The screening process consisted of two stages: title and abstract as well as full-text screening. The first stage involved 2 reviewers (MEB and MB) and one research assistant, who independently screened all records identified by the searches. This task was completed using a web-based application for systematic reviews, Rayyan [43]. The interrater reliability was excellent (agreement 96%; Cohen κ=0.834; Gwet AC1=0.950). All records with disagreement among the 3 reviewers were automatically included in the full-text screening stage. The full-text screening stage was completed by the first author with the help of a research assistant and verified by the fourth author. All disagreements were resolved through discussion.

### Stage 4: Charting the Data

For each retrieved record, 2 authors (MEB and MB) extracted the following information into a Microsoft Excel spreadsheet: first author name, year of publication, article title, journal, and number of trial registry (if available), principal investigator name (if available), country of the first author or of the principal investigator (if available). This information was used to identify and map articles pertaining to the same study. In the full-text stage, we also extracted text to verify whether the record included a digital component, was based on a randomized controlled design, focused on specific health conditions, and measured and reported results related to one of the domains of the eHealth literacy model (health, computer, basic or functional, information, media, and scientific literacy).

When multiple records were available for one study, we chose the country of origin of the first author or of the principal investigator listed in the study protocol; we chose the year of publication of the first published article available.

On the basis of the information extracted, we categorized studies according to the domains of the eHealth literacy model (ie, health, computer, basic or functional, information, media, and scientific literacy). We also inductively categorized the studies according to the health conditions described. When multiple conditions were reported, we categorized the study as having multiple conditions. Finally, we inductively categorized the interventions according to four main types of technology: (1) *mobile-based*, including mobile apps, text messages, and interactive voice response, exclusively designed for mobile or other handheld devices; (2) *web-based*, including those designed for being accessed via computer, explicitly labeled as *web- or internet-based*, *online*, and *e-learning*, delivered through bespoke websites or social media outlets, such as social networking sites (eg, Facebook or Twitter)—social media are web-based apps that can be accessed via different devices connected to the internet, including smartphones [39,44,45]; (3) *telehealth*, comprising telerehabilitation, telemedicine, or other interventions focused on distributing services and information via electronic information and telecommunication devices [46]; (4) *EHRs*, focusing on EHRs that are defined as “a repository of patient data in digital form, stored and exchanged securely, and accessible by multiple authorized users” [47]. Telehealth and EHR interventions use the internet to connect various devices, including tablets and mobile phones, yet they represent a different type of delivery mode and format: EHRs. We labeled interventions using a combination of the modes described earlier, as reported in other studies [6,48]. When studies reported a combination of the abovementioned categories, we categorized the study as a *hybrid*.

The first author and a research assistant independently completed the classifications; in case of inconsistencies or disagreements between the classifications, the fourth author acted as a third reviewer and resolved the disagreements through discussion. All the authors agreed with the final categorization.

### Stage 5: Collating, Summarizing, and Reporting the Results

We performed a descriptive analysis of the characteristics of the included papers and reported the results by year of publication, the country of origin of the study authors, eHealth literacy domain, health condition, and type of technology used.

## Results

### Search Results

**Figure 1:**
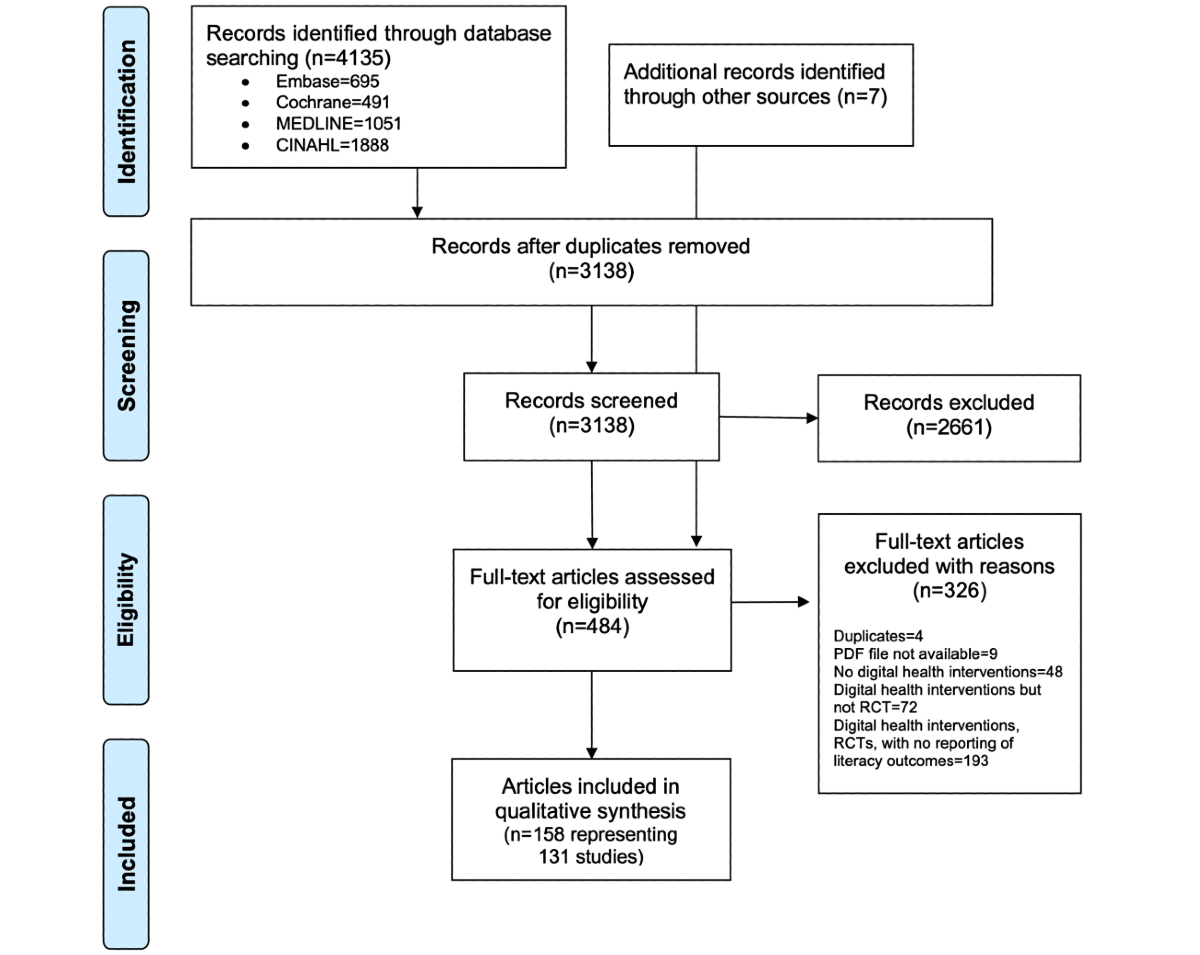
PRISMA (Preferred Reporting Items for Systematic Reviews and Meta-Analyses) flow diagram. RCT: randomized controlled trial.

The electronic database search yielded 4135 records. The selection process is summarized in the PRISMA (Preferred Reporting Items for Systematic Reviews and Meta-Analyses) flow diagram shown in Figure 1. Briefly, after removing duplicates, the titles and abstracts of 3138 records were independently screened by 3 reviewers. During the title and abstract screening, we excluded 2661 records that were deemed irrelevant. The remaining 477 records were assessed for eligibility in the full text. Scanning the reference lists of two relevant systematic reviews [5,49] allowed us to identify seven other eligible records. We evaluated the eligibility of 484 records that were screened in full text. Of these, 326 records were excluded for the following reasons: 48 did not discuss DHIs (wrong context); 72 reported on digital interventions but did not use an RCT or randomized clinical trial design (wrong study design); 193 records discussed DHIs but did not report any type of literacy (no relevant outcome assessed or reported); 4 were duplicate records; and for the remaining 9 records, we could not retrieve a PDF file. The list of excluded references is provided in Multimedia Appendix 2. Overall, we included 158 records: 79 records reported concluded interventions and 79 records reported protocols of ongoing studies, without reporting results. These were included because they described the assessment of some domains of the eHealth literacy model. The 158 records described a total of 131 unique studies.

### Characteristics of the Included Studies

#### Publication Year and Geographic Distribution

**Figure 2:**
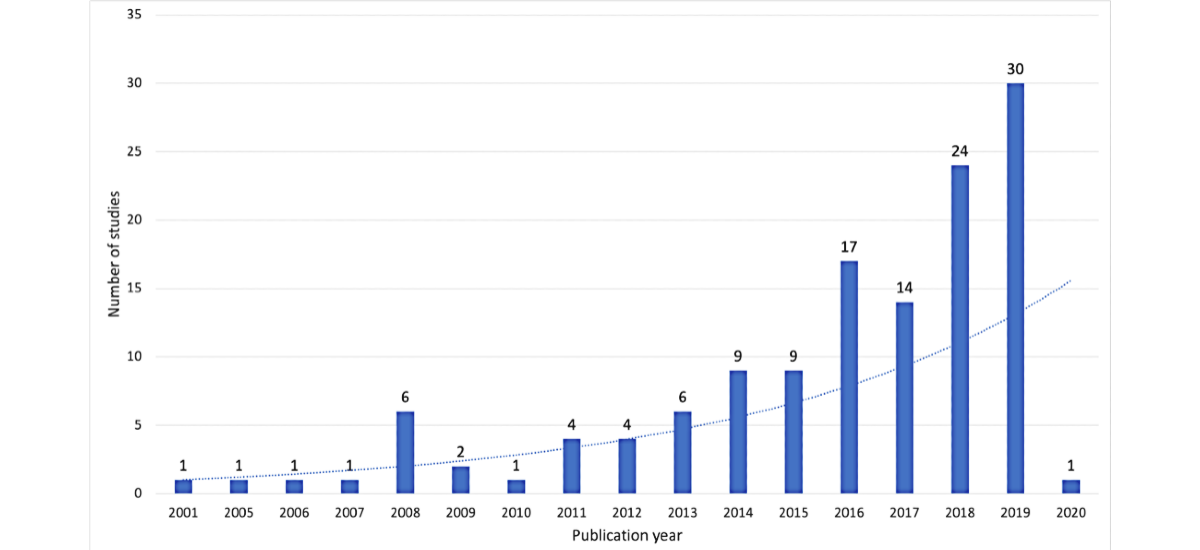
Distribution of selected studies (N=131) by year of publication.

As shown in Figure 2, most of the selected studies were conducted in the last 4 years (86/131, 65.6%), followed by an exponential trend, peaking in 2019 (30/131, 22.9%) and ranging from 2001 to 2020.

The studies were conducted in 26 countries (Table 1). Most studies were conducted across 3 countries (81/131, 61.8%), including the United States (43/131, 32.8%), Australia (28/131, 21.3%), and the United Kingdom (10/131, 7.6%). Approximately one-third of the studies (38/131, 29%) were conducted in European countries such as the United Kingdom (10/38, 26%); Germany (8/38, 21%); Denmark (5/38, 13%); Sweden (4/38, 11%); the Netherlands (3/38, 8%); Norway (2/38, 5%); and Belgium, Finland, Luxemburg, Ireland, Slovakia, and Switzerland (1/38, 3% each). Asian countries were represented by Iran (4/16, 25%); Turkey (3/16, 19%); Hong Kong (2/16, 13%); Singapore (2/16, 13%); Japan (2/16, 13%); and Jordan, Malaysia, and Pakistan (1/16, 6% each). Overall, only 0.8% (1/131) of studies were conducted in Africa (South Africa) and 22.1% (29/131) in Oceania (New Zealand: 1/29, 3%; Australia: 28/29, 97%).

In the following sections, we have reported the results according to our research objectives, whereas a table with the detailed characteristics of the selected studies is provided in Multimedia Appendix 3.

**Table 1:** Distribution of included studies by country (N=131).

Country	Studies, n (%)
United States	43 (32.8)
Australia	28 (21.3)
United Kingdom	10 (7.6)
Germany	8 (6.1)
Denmark	5 (3.8)
Sweden	4 (3.1)
Iran	4 (3.1)
Netherlands	3 (2.2)
Turkey	3 (2.2)
Brazil	2 (1.5)
Canada	2 (1.5)
Hong Kong	2 (1.5)
Japan	2 (1.5)
Norway	2 (1.5)
Singapore	2 (1.5)
Belgium	1 (0.7)
Finland	1 (0.7)
Ireland	1 (0.7)
Jordan	1 (0.7)
Luxemburg	1 (0.7)
Malaysia	1 (0.7)
New Zealand	1 (0.7)
Pakistan	1 (0.7)
Slovakia	1 (0.7)
South Africa	1 (0.7)
Switzerland	1 (0.7)

#### Domains of the eHealth Literacy Model Assessed

**Figure 3:**
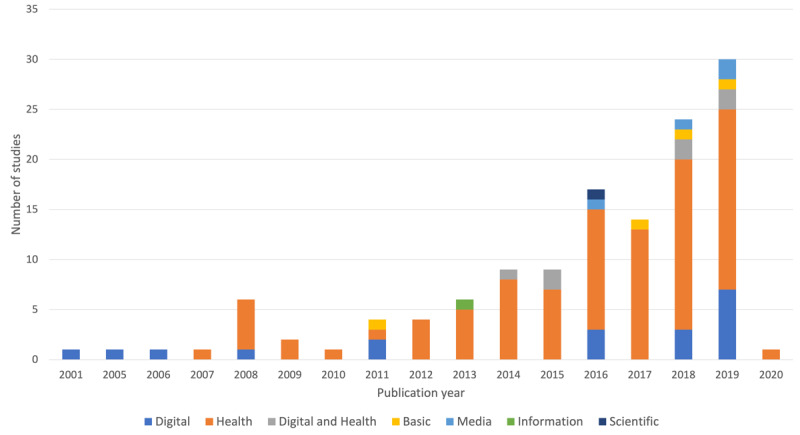
Distribution of studies (N=131) by eHealth literacy model domain.

Figure 3 presents the years of publication of the included studies grouped by domain of the eHealth literacy model. In total, 2.2% (3/131) of the included studies were published before 2006, when the seminal publications of the eHealth literacy model appeared [9,24]. These studies included an assessment of computer literacy. Studies published in 2008 mostly reported assessments of health literacy.

Of the 131 studies included, none assessed or measured all six domains of the eHealth literacy model. Most of the studies (124/131, 94.6%) focused on one of the six domains of the eHealth literacy model; only 5.3% (7/131) of studies reported the assessment of two domains, namely health literacy and digital or computer literacy. Most of the studies that reported on one literacy domain (124/131, 94.7%) focused on health literacy (95/124, 76.6%), followed by digital or computer literacy (19/124, 15.3%), basic or functional literacy (4/124, 3.2%), media literacy (4/124, 3.2%), information literacy (1/124, 0.8%), and scientific literacy (1/124, 0.8%).

#### Health Conditions Addressed and Technologies Used

Table 2 provides a summary of the selected studies grouped by technology category and health condition category.

A large number of studies (61/131, 46.5%) discussed interventions addressing NCDs, such as hypertension, obesity, end-stage kidney disease, type 2 diabetes, chronic kidney disease, heart disease (vascular disease, cerebrovascular disorders, ischemic heart disease, coronary artery disease, and heart failure), fibromyalgia syndrome, and asthma. Of these 61 NCD-focused studies, 3 (5%) also discussed mental health topics, and 1 (2%) covered sexual and reproductive health. The second most covered category of health conditions was mental health (26/131, 19.8%), including depression, eating disorders, mental and behavioral disorders, anxiety, and suicide prevention. Other topics included health education (16/131, 12.2%), such as health promotion, health communication, patient provider communication and literacy, aging and maternal and infant health (4/131, 3.0% of studies), sexual and reproductive health, and substance use (3/131, 2.2% of studies each). The remaining 11.4% (15/131) studies covered a variety of health topics.

**Table 2:** Number of studies by health condition category and type of technology used.

Health condition and technology used	Web based (n=68), n (%)	Mobile based (n=40), n (%)	Telehealth (n=10), n (%)	EHRs^a^ (n=5), n (%)	Hybrid (n=8), n (%)	Total (N=131), n (%)
NCDs^b^	22 (32.4)	19 (47.5)	6 (60)	3 (60)	6 (75)	56 (42.7)
NCDs—mental health	1 (1.5)	2 (5)	0 (0)	0 (0)	0 (0)	3 (2.3)
NCDs—sexual and reproductive health	0 (0)	0 (0)	0 (0)	0 (0)	1 (12.5)	1 (0.8)
Mental health	21 (30.9)	4 (10)	1 (10)	0 (0)	0 (0)	26 (19.8)
Aging	2 (2.9)	1 (2.5)	1 (10)	0 (0)	0 (0)	4 (3.1)
Health education topics	9 (13.2)	4 (10)	1 (10)	2 (40)	0 (0)	16 (12.2)
Maternal and infant health	2 (2.9)	2 (5)	0 (0)	0 (0)	0 (0)	4 (3.1)
Sexual and reproductive health	2 (2.9)	1 (2.5)	0 (0)	0 (0)	0 (0)	3 (2.3)
Substance use	2 (2.9)	1 (2.5)	0 (0)	0 (0)	0 (0)	3 (2.3)
Other health topics	7 (10.3)	6 (15)	1 (10)	0 (0)	1 (12.5)	15 (11.5)

^a^EHR: electronic health record.

^b^NCD: noncommunicable disease.

With regard to the technologies used, most studies included web-based interventions (68/131, 51.9%), followed by mobile-based (40/131, 30.5%), telehealth (10/131, 7.6%) EHRs (5/131, 3.8%), and hybrid interventions (8/131, 6.1%). Examples of web-based technology included e-learning portals for specialized training [50,51], experimental websites, and social media platforms [52-54], which are used to deliver motivational or informational campaigns. Mobile-based interventions included health apps [55-57], SMS text messaging or WhatsApp [58], games [59,60], and interactive voice response [61,62]. Telehealth interventions included rehabilitation programs [63,64] or remote counseling [65]. Hybrid interventions included combinations of mobile apps and EHRs [55-57], SMS text messaging and EHRs [66], or a mix of web- and mobile-based technologies [67].

Among web-based interventions (n=68), most focused on NCDs (22/68, 32%), mental health (21/68, 31%), and health education topics (9/68, 13%). Mobile-based interventions (n=40) followed a similar pattern, with approximately half of the studies focusing on NCDs (19/40, 48%) or other health topics (6/40, 15%). Most telehealth (6/10, 60%), EHR (3/5, 60%), and hybrid (6/8, 75%) interventions focused on NCDs.

## Discussion

### Principal Findings

This is the first scoping review examining the extent to which DHIs have assessed, accounted for, and reported any of the six domains of the eHealth literacy model by Norman and Skinner [9]. We identified a sizable literature discussing DHIs developed in 26 countries, spanning two decades. The eHealth literacy model [9] and eHEALS [24] date back to 2006, but we included 3 studies that were published before that year and all assessed computer literacy. This might indicate that attention toward the ability to use technology was a research interest in the early 2000s. However, this interest has not grown exponentially and concomitantly with the growth of DHIs. It is interesting to observe that the assessment of digital literacy has grown only after 2015, but it has remained below the assessment of health literacy, which was the domain assessed the most over time. There is no clear explanation for these trends. A bibliometric analysis of the studies cited in the seminal papers mentioned above could reveal the connections between publications and demonstrate when the eHealth literacy model has received more citations.

Most of the evidence comes from the *Global North*, that is, from English-speaking countries including the United States, Australia, and the United Kingdom. A few studies have been conducted in countries of the *Global South*, such as Africa, Latin America, or South East Asia. This finding is consistent with that reported in a recent scoping review on digital health innovations [68] and in a recent bibliometric analysis of research on mHealth apps [69], which showed a predominance of articles published in the United States, the United Kingdom, Australia, and Canada. Publication bias and limited evidence from developing countries or the *Global South* has been previously reported in the literature [70-72], yet there seems to be a lack of evidence on DHIs from Africa, the Middle East, South America, or Southeast Asia. There may be various reasons for this absence of evidence. First, research on digital technologies might not have reached an advanced stage to produce interventions with the highest level of evidence (ie, RCTs). Second, the existing digital divide might persist in many countries, both low- and high-income countries [30]; however, mobile phones and telemedicine are becoming more widely adopted [46,73]. Third, researchers based in low- and middle-income countries (LMICs) may be published in languages other than English or might have limited English language proficiency, but this latter assumption does not seem to be grounded in evidence [74,75]. Another reason might be that researchers in LMICs might choose to publish in journals that are not indexed in the databases we searched. Alternatively, researchers in LMICs might not have the possibility to publish their results because of a lack of funding for open access publications or because editors demonstrate publication bias [72]. Regardless of the reasons, we call for digital health researchers based in countries of the Global South to publish more study protocols and diffuse intervention results; we also call the international community of editors and publishing houses to incentivize or support research published from these underrepresented countries, so that stronger conclusions can be drawn from a truly global evidence base.

### Domains of the eHealth Literacy Model Assessed

Our findings showed that none of the 131 selected DHIs conducted in the last 20 years accounted for or assessed all six domains of the eHealth literacy model. Although these interventions were included because they assessed at least one domain of the model, only 5.3% (7/131) of studies included the assessment of more than one domain. These 7 studies assessed only digital and health literacy. Our study also shows that most DHIs have assessed and evaluated health literacy [19] among intervention participants, which is an important factor that can determine the health outcomes of a study [21,31,32]. Although the focus on health literacy in DHIs is consistent with some literature reviews combining the study of health literacy identified through our searches [5,22,26,34,35,49], it is somewhat surprising that none of the other four domains of the eHealth literacy model were concomitantly addressed.

There are numerous explanations for these findings. First, researchers specialized in DHIs might not be familiar with or might have ignored the original model, even though the seminal paper by Norman and Skinner [9] and the paper describing the eHEALS [24] are highly cited (as of October 17, 2020, Google Scholar showed 1128 and 1450 citations, respectively). Second, researchers might have decided to focus on other domains of the model while making implicit or explicit assumptions about the levels of literacy in other domains. For example, the limited evidence related to the assessment of the domains of scientific, information, media, and functional literacy might be based on the assumption that digital literacy instruments, such as the popular eHEALS [24], include questions related to the use of information on the internet as a medium of search information; hence, these could be associated with media and information literacy domains. However, there exist several instruments that specifically assess media literacy [76,77], scientific literacy [78,79], and information literacy [80,81]. Moreover, Norman and Skinner [9] did not consider overlapping elements when they developed the eHealth literacy model, which considers the six domains as distinct and separate.

Although intervention designers should aim to develop content that is understood by people with low functional literacy [82,83], this fact should be proven or verified by the same intervention designers. One way to do so is to assess functional literacy or to report the level of literacy rather than to just develop the content of the intervention through formal readability and usability testing. The fact that other domains of the eHealth literacy model were not always conducted raises concerns about the generalizability of such interventions across the eHealth literacy spectrum. DHIs tend to attract tech-savvy, healthy volunteers who have access to technology and who might have different sociodemographic and psychological profiles compared with people who belong to vulnerable segments of the population and do not have access to technology [30].

Another important finding was that few identified DHIs assessed digital literacy (n=26). Not assessing digital literacy is based on the assumption that all participants are equally able to use technology and are able to make sense of the information delivered. This assumption might not be tenable in all contexts, and it does not allow researchers to understand whether participants appropriately received the intervention. In other words, health literacy is context-specific and varies according to different situations and topics. Arguably, health and digital literacy might act as moderators of intervention effects and not including these factors might underestimate or overestimate intervention effects [84].

The limited assessment of digital literacy in DHIs also raises some ethical considerations in terms of equity and social justice, as these interventions tend to attract highly educated, healthy, and digitally literate individuals who have easy access to technology, leaving out less-educated and poorer segments of the population, who may be most in need of the interventions themselves [30,85]. This selection bias isolates segments of the population that are traditionally difficult to reach [86,87], yet it is important to acknowledge that the results of DHIs might be less generalizable than interventions that do not use technology.

Another reason for the absence of a comprehensive and accurate assessment of the six domains of the eHealth literacy model might be due to the fact that this assessment will be unfeasible and daunting for the participants. Holding constant the basic or functional literacy (ie, numeracy and ability to read), assessing all six domains using existing scales for media, scientific, health, digital, and information literacy would require longer questionnaires that will take more time to complete, which might discourage participation in these studies. For example, one of the most used instruments to assess digital literacy is the relatively short (8 items) eHEALS [24]. However, for context-specific domains such as health literacy [9], there are many more instruments available, which vary in length and complexity [25,88]. A recent review identified 43 different instruments [89], and the Health Literacy Toolshed database included 200 measures [90]. Similar issues of measurement pertain to the assessment of literacy in a digital world [91], including media literacy [92]. Nevertheless, we urge digital health researchers to find ways to assess and evaluate the different domains of the eHealth literacy model, so that they can gain a better understanding of the study participants’ characteristics, abilities, and needs. If measuring all domains might appear unfeasible, we suggest that DHI researchers prioritize the assessment of digital literacy—using the short eHEALS [24]—and health literacy, which is context specific, according to the model by Norman and Skinner [9]. Once the health topic or context is defined (mental health, breast cancer, etc), the choice of a short, yet valid instrument to assess health literacy in that context would become easier. As digital health and health literacy can change due to the intervention itself, we recommend assessing these constructs before and after the intervention. Finally, media, scientific, and traditional literacy are analytical skills that are not specific to any context; it would be easier for researchers to routinely assess these domains before the start of any intervention.

### Health Conditions Addressed and Technologies Used

This scoping review showed that the selected DHIs published in the last 20 years focused mostly on NCDs, delivered via web- or mobile-based platforms. This is consistent with the findings of a few recent scoping reviews focusing on research on DHIs for behavior change [93,94] or in a recent bibliometric analysis of mHealth apps [69]. Although most DHIs have covered NCDs and mental health, there are many avenues for digital health. Further systematic reviews could be developed to specifically qualify and quantify the effectiveness of DHIs delivered via web or mobile phones in reducing NCDs and mental health issues. These systematic reviews could also anticipate sensitivity analyses based on the modes of delivery, length of the interventions, or the assessment of eHealth literacy model domains. This scoping review provides a valuable map of the evidence and sets the research agenda for DHIs in the coming years.

### Strengths and Limitations

To the best of our knowledge, this is the first scoping review that systematically examined evidence pertaining to the application of the eHealth literacy model by Norman and Skinner [9] in DHIs. We looked at the highest quality of evidence, following a predetermined search strategy and a systematic approach to appraise the literature, without restricting our searches to specific periods, populations, countries, or health conditions. Nevertheless, this study has some limitations that are common to many other systematic or scoping reviews. These limitations include the fact that we looked only at peer-reviewed articles available in English. It is possible that some evidence on the use of the eHealth literacy model could have been reported in non–peer-reviewed or gray literature. Another limitation is related to the use of an RCT filter and focus on the RCT study design. Although RCTs provide the highest level of evidence, according to Grading of Recommendations Assessment, Development and Evaluation standards [40], it might be possible that some relevant research entailed the use of other types of study designs.

### Conclusions

This review suggests that future DHIs should focus more on the assessment of the eHealth literacy domains when developing a DHI, especially the domains that are assessed the least, such as scientific, media, basic, and information literacy. Even though assessing all domains of the eHealth literacy model might be unfeasible, it would allow researchers to account for factors that might moderate or mediate the effects of the interventions on the targeted health outcomes.

Future systematic reviews should be conducted to examine the effects of DHIs on various health outcomes identified in this review by anticipating subgroup or sensitivity analyses comparing different types of intervention, delivery modes, and most importantly different levels of health literacy or digital literacy.

## Appendix

Multimedia Appendix 1 MEDLINE search strategy.

Multimedia Appendix 2 Records excluded during the full-text screening phase with full citations.

Multimedia Appendix 3 Characteristics of included studies with references.

## Abbreviations

DHI: digital health intervention
eHEALS: eHealth literacy scale
EHR: electronic health record
LMIC: low- and middle-income country
mHealth: mobile health
NCD: noncommunicable disease
PCC: Population-Concept-Context
PRISMA: Preferred Reporting Items for Systematic Reviews and Meta-Analyses
RCT: randomized controlled trial
