# Supplementary material for: Application of the eHealth Literacy Model in Digital Health Interventions: Scoping Review
Source: J Med Internet Res. 2021 Jun 3;23(6):e23473. doi: 10.2196/23473 (PMC8212628; doi:10.2196/23473)
Supplement: Multimedia Appendix 1 [file jmir_v23i6e23473_app1.docx]

## Multimedia Appendix 1 Medline Search Strategy

Database: Ovid MEDLINE(R) and Epub Ahead of Print, In-Process & Other Non-Indexed Citations and Daily <1946 to January 24, 2020>

Search Strategy:

--------------------------------------------------------------------------------

1 consumer health information/ or exp health literacy/ or literacy/ or computer literacy/ or information literacy/

2 ((basic or functional or scientific or media or information or computer or health or eHealth) adj1 (literacy or literate* or illiteracy or illiterate*)).mp.

3 1 or 2

4 exp Telemedicine/

5 (teleradiology or tele-radiology or telepathology or tele-pathology or tele-cardiology or telecardiology or teleconsultation or tele-consultation or teledermatology or tele-dermatology or telediagnos* or tele-diagnos* or telemonitoring or tele-monitoring or telepsychiatry or tele-psychiatry or teleradiotherapy or tele-radiotherapy or telerehabilitation or tele-rehabilitation or telesurgery or tele-surgery or teletherapy or tele-therapy or (remote adj (rehabilitation or consultation* or diagnos* or monitoring)) or erehabilitation or e-rehabilitation or (virtual adj rehabilitaiton)).mp.

6 Internet/

7 (mobile adj (app? or application? or phone?)).mp.

8 mobile applications/

9 ((mobile or digital) adj2 (medium or media* or network* or application? or app?)).mp.

10 (internet or online or web or web2).mp.

11 "web 2.0".mp.

12 (ehealth or mhealth or e-health or m-health or mHealth or eHealth or tele-medicine or telemedicine or telehealth).mp.

13 ((digital or mobile or electronic or computer or internet) adj1 health).mp.

14 or/4-13

15 ((randomized controlled trial or controlled clinical trial).pt. or randomized.ab. or placebo.ab. or drug therapy.fs. or randomly.ab. or trial.ab. or groups.ab.) not (exp animals/ not humans.sh.)

16 3 and 14 and 15 (1061)
