# Supplementary material for: Application of the eHealth Literacy Model in Digital Health Interventions: Scoping Review
Source: J Med Internet Res. 2021 Jun 3;23(6):e23473. doi: 10.2196/23473 (PMC8212628; doi:10.2196/23473)
Supplement: Multimedia Appendix 2 [file jmir_v23i6e23473_app2.docx]

## Multimedia Appendix 2: Records excluded at the full-text screening phase with full citation

Notes: References are in JMIR reference style. Reasons for exclusion are indicated in the heading of each section

[Wrong context: Not digital interventions (n=48) 1](#_Toc47370657)

[No digital, no RCT study assessing some type of literacy (n=5) 1](#_Toc47370658)

[No digital, no RCT study, not assessing any type of literacy (n=7) 1](#_Toc47370659)

[No digital, Systematic Review not assessing any type of literacy (n=2) 2](#_Toc47370660)

[RCT not using digital, measuring some type of literacy (n=14) 2](#_Toc47370661)

[RCT not using digital, not measuring any type of literacy (n=20) 3](#_Toc47370662)

[Wrong study design: Not RCT (n=72) 5](#_Toc47370663)

[Digital intervention, not RCT, assessing some type of literacy (n=26) 5](#_Toc47370664)

[Digital intervention, not RCT, not assessing any type of literacy (n=44) 7](#_Toc47370665)

[Relevant systematic reviews on DHI or health literacy (n=2) 10](#_Toc47370666)

[No relevant outcome assessed or reported: DHI, RCTs, not assessing any type of literacy (n=193) 10](#_Toc47370667)

[Duplicates (n=4) 25](#_Toc47370668)

[PDF not available (n=9) 26](#_Toc47370669)

## Wrong context: Not digital interventions (n=48)

### No digital, no RCT study assessing some type of literacy (n=5)

1. Cranley N, Curbow B, George T, Christie J, Williams K. Differences in treatment equity among newly diagnosed patients with colorectal cancer. Psycho-Oncology. 2017 2017;26:102.

2. Dashti S, Peyman N, Tajfard M, Esmaeeli H. E-Health literacy of medical and health sciences university students in Mashhad, Iran in 2016: a pilot study. Electron Physician. 2017 Mar;9(3):3966-73. PMID: 28461871. doi: 10.19082/3966.

3. Dolan JG, Cherkasky OA, Chin N, Veazie PJ. Decision Aids: The Effect of Labeling Options on Patient Choices and Decision Making. Med Decis Making. 2015 Nov;35(8):979-86. PMID: 26229084. doi: 10.1177/0272989X15598532.

4. Park H, Lee E. Self-reported eHealth literacy among undergraduate nursing students in South Korea: a pilot study. Nurse Educ Today. 2015 Feb;35(2):408-13. PMID: 25466791. doi: 10.1016/j.nedt.2014.10.022.

5. Samal L, Yeh HC, Gary-Webb TL, Jackson CL, Brancati FL. Computer and internet use of urban african americans with type 2 diabetes in relation to glycemic control, emergency department use, diabetes-related knowledge, and health literacy. Diabetes care. 2010 2010;33(1):e9. PMID: CN-00728724.

### No digital, no RCT study, not assessing any type of literacy (n=7)

1. Bauer AM, Thielke SM, Katon W, Unutzer J, Arean P. Aligning health information technologies with effective service delivery models to improve chronic disease care. Prev Med. 2014 Sep;66:167-72. PMID: 24963895. doi: 10.1016/j.ypmed.2014.06.017.

2. Damodaran L, Olphert CW, Sandhu J. Falling off the bandwagon? Exploring the challenges to sustained digital engagement by older people. Gerontology. 2014;60(2):163-73. PMID: 24356488. doi: 10.1159/000357431.

3. Druce I, Williams C, Baggoo C, Keely E, Malcolm J. A Comparison of Patient and Healthcare Professional Views When Assessing Quality of Information on Pituitary Adenoma Available on the Internet. Endocr Pract. 2017 Oct;23(10):1217-22. PMID: 28816540. doi: 10.4158/EP171892.OR.

4. Lawrentschuk N, Abouassaly R, Hackett N, Groll R, Fleshner NE. Health information quality on the internet in urological oncology: a multilingual longitudinal evaluation. Urology. 2009 Nov;74(5):1058-63. PMID: 19758687. doi: 10.1016/j.urology.2009.05.091.

5. Perret JL, Bonevski B, McDonald CF, Abramson MJ. Smoking cessation strategies for patients with asthma: improving patient outcomes. J Asthma Allergy. 2016 2016;9:117-28. PMID: 27445499. doi: 10.2147/JAA.S85615.

6. Taha J, Sharit J, Czaja S. Use of and satisfaction with sources of health information among older Internet users and nonusers. Gerontologist. 2009 Oct;49(5):663-73. PMID: 19741112. doi: 10.1093/geront/gnp058.

7. Thies K, Anderson D, Cramer B. Lack of Adoption of a Mobile App to Support Patient Self-Management of Diabetes and Hypertension in a Federally Qualified Health Center: Interview Analysis of Staff and Patients in a Failed Randomized Trial. JMIR Hum Factors. 2017 Oct 3;4(4):e24. PMID: 28974481. doi: 10.2196/humanfactors.7709.

### No digital, Systematic Review not assessing any type of literacy (n=2)

1. Jalil S, Myers T, Atkinson I. A meta-synthesis of behavioral outcomes from telemedicine clinical trials for type 2 diabetes and the Clinical User-Experience Evaluation (CUE). J Med Syst. 2015 Mar;39(3):28. PMID: 25677954. doi: 10.1007/s10916-015-0191-9.

2. Madathil KC, Rivera-Rodriguez AJ, Greenstein JS, Gramopadhye AK. Healthcare information on YouTube: A systematic review. Health Informatics J. 2015 Sep;21(3):173-94. PMID: 24670899. doi: 10.1177/1460458213512220.

### RCT not using digital, measuring some type of literacy (n=14)

1. Actrn. Increasing rates of eating disorder detection and treatment in adolescence. http://wwwwhoint/trialsearch/Trial2aspx?TrialID=ACTRN12613000199718. 2013 2013. PMID: CN-01858797.

2. Actrn. Randomised comparison of how different terminologies for papillary thyroid cancer affect decision making about treatment among an Australian community sample. http://wwwwhoint/trialsearch/Trial2aspx?TrialID=ACTRN12616000271404. 2016 2016. PMID: CN-01817602.

3. Actrn. The Melanoma Genomics Managing Your Risk Study: examining the impact of personal melanoma genomic risk information on prevention behaviours in the general population. http://wwwwhoint/trialsearch/Trial2aspx?TrialID=ACTRN12617000691347. 2017 2017. PMID: CN-01886454.

4. Actrn. The Workplace Mental Health Intervention program for the enhancement of Mental Health Literacy and the reduction of stress and burnout among workers: the (WPMHL) Project. http://wwwwhoint/trialsearch/Trial2aspx?TrialID=ACTRN12619000464167. 2019 2019. PMID: CN-01972178.

5. Barker A, Cameron P, Flicker L, Arendts G, Brand C, Etherton-Beer C, et al. Evaluation of RESPOND, a patient-centred program to prevent falls in older people presenting to the emergency department with a fall: A randomised controlled trial. PLoS Med. 2019 May;16(5):e1002807. PMID: 31125354. doi: 10.1371/journal.pmed.1002807.

6. Drks. Lifestyle Intervention in Chronic ischemic heart disease and Diabetes. http://wwwwhoint/trialsearch/Trial2aspx?TrialID=DRKS00015140. 2019 2019. PMID: CN-01949444.

7. Heckel L, Fennell KM, Reynolds J, Boltong A, Botti M, Osborne RH, et al. Efficacy of a telephone outcall program to reduce caregiver burden among caregivers of cancer patients [PROTECT]: A randomised controlled trial. BMC Cancer. 2018 2018;18(1).

8. Isrctn. Promoting mental health and wellbeing in schools: examining Youth Aware of Mental Health (YAM) and the Mental Health & High School Curriculum Guide (The Guide) in English secondary schools. http://wwwwhoint/trialsearch/Trial2aspx?TrialID=ISRCTN17631228. 2018 2018. PMID: CN-01905729.

9. McCarthy ML, Ding R, Roderer NK, Steinwachs DM, Ortmann MJ, Pham JC, et al. Does providing prescription information or services improve medication adherence among patients discharged from the emergency department? A randomized controlled trial. Annals of Emergency Medicine. 2013 2013;62(3):212-23e1.

10. McCormack L, Craig Lefebvre R, Bann C, Taylor O, Rausch P. Consumer Understanding, Preferences, and Responses to Different Versions of Drug Safety Messages in the United States: A Randomized Controlled Trial. Drug Saf. 2016 Feb;39(2):171-84. PMID: 26547718. doi: 10.1007/s40264-015-0358-9.

11. McGrew S, Smith M, Breakstone J, Ortega T, Wineburg S. Improving university students' web savvy: An intervention study. Br J Educ Psychol. 2019 Sep;89(3):485-500. PMID: 30993684. doi: 10.1111/bjep.12279.

12. Ntr. Effectiveness of Moving Stories, a game-based school program aimed at depression literacy and stigma. http://wwwwhoint/trialsearch/Trial2aspx?TrialID=NTR7033. 2018 2018. PMID: CN-01896991.

13. Reder M, Soellner R, Kolip P. Do Women With High eHealth Literacy Profit More From a Decision Aid on Mammography Screening? Testing the Moderation Effect of the eHEALS in a Randomized Controlled Trial. Front Public Health. 2019;7:46. PMID: 30931291. doi: 10.3389/fpubh.2019.00046.

14. Wong A, Rodriguez-Nunez A, Tayjasanant S, Park M, Liu DD, Allo J, et al. Edmonton Symptom Assessment Scale (ESAS): Time duration of self-completion versus assistedcompletion in palliative care patients-A randomized controlled trial. Journal of Clinical Oncology. 2019 2019;34(26):67.

### RCT not using digital, not measuring any type of literacy (n=20)

1. Domnich A, Panatto D, Signori A, Bragazzi NL, Cristina ML, Amicizia D, et al. Uncontrolled Web-based administration of surveys on factual health-related knowledge: a randomized study of untimed versus timed quizzing. J Med Internet Res. 2015 Apr 13;17(4):e94. PMID: 25872617. doi: 10.2196/jmir.3734.

2. Folkvord F, Lupiáñez-Villanueva F, Codagnone C, Bogliacino F, Veltri G, Gaskell G. Does a 'protective' message reduce the impact of an advergame promoting unhealthy foods to children? An experimental study in Spain and The Netherlands. Appetite. 2017 2017;112:117‐23. PMID: CN-01332415.

3. Francis NA, Hood K, Simpson S, Wood F, Nuttall J, Butler CC. The effect of using an interactive booklet on childhood respiratory tract infections in consultations: study protocol for a cluster randomised controlled trial in primary care. BMC family practice. 2008 2008;9:23. PMID: CN-00639172.

4. Hart LM, Cropper P, Morgan AJ, Kelly CM, Jorm AF. teen Mental Health First Aid as a school-based intervention for improving peer support of adolescents at risk of suicide: Outcomes from a cluster randomised crossover trial. Aust N Z J Psychiatry. 2019 Nov 9:4867419885450. PMID: 31707787. doi: 10.1177/0004867419885450.

5. Hart LM, Morgan AJ, Rossetto A, Kelly CM, Mackinnon A, Jorm AF. Helping adolescents to better support their peers with a mental health problem: A cluster-randomised crossover trial of teen Mental Health First Aid. Aust N Z J Psychiatry. 2018 Jul;52(7):638-51. PMID: 29417834. doi: 10.1177/0004867417753552.

6. Hester KL, Newton J, Rapley T, De Soyza A. Evaluation of a novel information resource for patients with bronchiectasis: study protocol for a randomised controlled trial. Trials. 2016 Apr 23;17(1):210. PMID: 27107959. doi: 10.1186/s13063-016-1330-4.

7. Isrctn. An intervention study to improve food shopping and prevent cardiovascular disease among patients in primary care: the PC SHOP trial. http://wwwwhoint/trialsearch/Trial2aspx?TrialID=ISRCTN14279335. 2017 2017. PMID: CN-01894891.

8. Kinsella Y, Gross M, Shin N, Aginam N, Jones S, Carpenter CR, et al. Use of teachback discharge instructions does not improve patient satisfaction in the ED. Academic emergency medicine. 2013 2013;20(5):S225‐. PMID: CN-01007790.

9. Labiner-Wolfe J, Jordan Lin CT, Verrill L. Effect of low-carbohydrate claims on consumer perceptions about food products' healthfulness and helpfulness for weight management. Journal of Nutrition Education and Behavior. 2010 2010;42(5):315-20.

10. Lo Siou G, Csizmadi I, Boucher BA, Akawung AK, Whelan HK, Sharma M, et al. The Comparative Reliability and Feasibility of the Past-Year Canadian Diet History Questionnaire II: Comparison of the Paper and Web Versions. Nutrients. 2017 Feb 13;9(2). PMID: 28208819. doi: 10.3390/nu9020133.

11. Meyers N, Glick AF, Mendelsohn AL, Parker RM, ers LM, Wolf MS, et al. Parents’ Use of Technologies for Health Management: a Health Literacy Perspective. Academic pediatrics. 2019 2019. PMID: CN-01940569.

12. Mittaz Hager A-G, Mathieu N, Lenoble-Hoskovec C, Swanenburg J, de Bie R, Hilfiker R. Effects of three home-based exercise programmes regarding falls, quality of life and exercise-adherence in older adults at risk of falling: protocol for a randomized controlled trial. BMC Geriatrics. 2019 2019;19(1):1-11. PMID: 134124561.

13. Nct. Sleep Health Literacy in Head Start. https://clinicaltrialsgov/show/NCT03556462. 2018 2018. PMID: CN-01609229.

14. Nct. Wellness Champions for Change. https://clinicaltrialsgov/show/NCT03432715. 2018 2018. PMID: CN-01522951.

15. Nct. Home-based Prediabetes Care in Acoma Pueblo - Study 1. https://clinicaltrialsgov/show/NCT04029298. 2019 2019. PMID: CN-01965715.

16. Strasser AA, Orom H, Tang KZ, Dumont RL, Cappella JN, Kozlowski LT. Graphic-enhanced information improves perceived risks of cigar smoking. Addict Behav. 2011 Aug;36(8):865-9. PMID: 21481542. doi: 10.1016/j.addbeh.2011.03.005.

17. Subramanian L, Zhao J, Zee J, Knaus M, Fagerlin A, Perry E, et al. Use of a Decision Aid for Patients Considering Peritoneal Dialysis and In-Center Hemodialysis: A Randomized Controlled Trial. Am J Kidney Dis. 2019 Sep;74(3):351-60. PMID: 30954312. doi: 10.1053/j.ajkd.2019.01.030.

18. Szrek H, Bundorf MK. Enrollment in prescription drug insurance: the interaction of numeracy and choice set size. Health Psychol. 2014 Apr;33(4):340-8. PMID: 23795708. doi: 10.1037/a0032738.

19. Ventura F, Sawatzky R, Öhlén J, Karlsson P, Koinberg I. Evaluation of a web-based educational program for women diagnosed with breast cancer: why is the intervention effect absent? Studies in health technology and informatics. 2013 2013;192:1132.

20. Wong CL, Mendoza J, Henson SJ, Qi Y, Lou W, L'Abbe MR. Consumer attitudes and understanding of cholesterol-lowering claims on food: randomize mock-package experiments with plant sterol and oat fibre claims. Eur J Clin Nutr. 2014 Aug;68(8):946-52. PMID: 24918122. doi: 10.1038/ejcn.2014.107.

## Wrong study design: Not RCT (n=72)

### Digital intervention, not RCT, assessing some type of literacy (n=26)

1. Ahmadvand A, Drennan J, Burgess J, Clark M, Kavanagh D, Burns K, et al. Novel augmented reality solution for improving health literacy around antihypertensives in people living with type 2 diabetes mellitus: protocol of a technology evaluation study. BMJ Open. 2018 Apr 28;8(4):e019422. PMID: 29705754. doi: 10.1136/bmjopen-2017-019422.

2. Brewer LC, Jenkins S, Lackore K, Johnson J, Jones C, Cooper LA, et al. mHealth Intervention Promoting Cardiovascular Health Among African-Americans: Recruitment and Baseline Characteristics of a Pilot Study. JMIR Res Protoc. 2018 Jan 31;7(1):e31. PMID: 29386174. doi: 10.2196/resprot.8842.

3. Dorst MT, Anders SH, Chennupati S, Chen Q, Purcell Jackson G. Health Information Technologies in the Support Systems of Pregnant Women and Their Caregivers: Mixed-Methods Study. J Med Internet Res. 2019 May 9;21(5):e10865. PMID: 31094327. doi: 10.2196/10865.

4. Dworkin MS, Lee S, Chakraborty A, Monahan C, Hightow-Weidman L, Garofalo R, et al. Acceptability, Feasibility, and Preliminary Efficacy of a Theory-Based Relational Embodied Conversational Agent Mobile Phone Intervention to Promote HIV Medication Adherence in Young HIV-Positive African American MSM. AIDS Educ Prev. 2019 Feb;31(1):17-37. PMID: 30742481. doi: 10.1521/aeap.2019.31.1.17.

5. Fernandez-Gutierrez M, Bas-Sarmiento P, Poza-Mendez M. Effect of an mHealth Intervention to Improve Health Literacy in Immigrant Populations: A Quasi-experimental Study. Comput Inform Nurs. 2019 Mar;37(3):142-50. PMID: 30531321. doi: 10.1097/CIN.0000000000000497.

6. Heldenbrand S, Martin BC, Gubbins PO, Hadden K, Renna C, Shilling R, et al. Assessment of medication adherence app features, functionality, and health literacy level and the creation of a searchable Web-based adherence app resource for health care professionals and patients. J Am Pharm Assoc (2003). 2016 May-Jun;56(3):293-302. PMID: 27067551. doi: 10.1016/j.japh.2015.12.014.

7. Jackson GP, Robinson JR, Ingram E, Masterman M, Ivory C, Holloway D, et al. A technology-based patient and family engagement consult service for the pediatric hospital setting. J Am Med Inform Assoc. 2018 Feb 1;25(2):167-74. PMID: 29016789. doi: 10.1093/jamia/ocx067.

8. Kamis K, Janevic MR, Marinec N, Jantz R, Valverde H, Piette JD. A study of mobile phone use among patients with noncommunicable diseases in La Paz, Bolivia: implications for mHealth research and development. Global Health. 2015 Jul 4;11:30. PMID: 26141528. doi: 10.1186/s12992-015-0115-y.

9. Khan D, Fjerbæk A, Andreasen JJ, Thorup CB, Dinesen B. Cardiac surgery patients’ e-health literacy and their use of a digital portal. Health Education Journal. 2018 2018;77(4):482-94.

10. Knudsen MV, Petersen AK, Angel S, Hjortdal VE, Maindal HT, Laustsen S. Tele-rehabilitation and hospital-based cardiac rehabilitation are comparable in increasing patient activation and health literacy: A pilot study. Eur J Cardiovasc Nurs. 2019 Nov 8:1474515119885325. PMID: 31702397. doi: 10.1177/1474515119885325.

11. Lin W-Y, Zhang X, Cao B. How Do New Media Influence Youths' Health Literacy? Exploring the Effects of Media Channel and Content on Safer Sex Literacy. International Journal of Sexual Health. 2018 2018;30(4):354-65. PMID: 134995692.

12. Mackert M, Kahlor L, Tyler D, Gustafson J. Designing e-health interventions for low-health-literate culturally diverse parents: addressing the obesity epidemic. Telemed J E Health. 2009 Sep;15(7):672-7. PMID: 19694596. doi: 10.1089/tmj.2009.0012.

13. Manafò E, Wong S. eSEARCH©: A Tool to Promote the eHealth Literacy Skills of Older Adults. Journal of Consumer Health on the Internet. 2013 2013;17(3):255-71. PMID: 104211392.

14. Mangam K, Fiekowsky E, Bagayoko M, Norris L, Belemvire A, Longhany R, et al. Feasibility and Effectiveness of mHealth for Mobilizing Households for Indoor Residual Spraying to Prevent Malaria: A Case Study in Mali. Glob Health Sci Pract. 2016 Jun 20;4(2):222-37. PMID: 27353616. doi: 10.9745/GHSP-D-15-00381.

15. Nct. INTERGENERATIONAL MOBILE TECHNOLOGY OPPORTUNITIES PROGRAM IN TAIWAN. https://clinicaltrialsgov/show/NCT02971241. 2016 2016. PMID: CN-01560159.

16. Nelson LA, Mulvaney SA, Gebretsadik T, Ho YX, Johnson KB, Osborn CY. Disparities in the use of a mHealth medication adherence promotion intervention for low-income adults with type 2 diabetes. J Am Med Inform Assoc. 2016 Jan;23(1):12-8. PMID: 26186935. doi: 10.1093/jamia/ocv082.

17. Nijland N, van Gemert-Pijnen JE, Kelders SM, Brandenburg BJ, Seydel ER. Factors influencing the use of a Web-based application for supporting the self-care of patients with type 2 diabetes: a longitudinal study. J Med Internet Res. 2011 Sep 30;13(3):e71. PMID: 21959968. doi: 10.2196/jmir.1603.

18. Nyberg A, Wadell K, Lindgren H, Tistad M. Internet-based support for self-management strategies for people with COPD-protocol for a controlled pragmatic pilot trial of effectiveness and a process evaluation in primary healthcare. BMJ Open. 2017 Aug 1;7(7):e016851. PMID: 28765136. doi: 10.1136/bmjopen-2017-016851.

19. Peate M, Smith SK, Pye V, Hucker A, Stern C, Stafford L, et al. Assessing the usefulness and acceptability of a low health literacy online decision aid about reproductive choices for younger women with breast cancer: the aLLIAnCE pilot study protocol. Pilot Feasibility Stud. 2017;3:31. PMID: 28603643. doi: 10.1186/s40814-017-0144-9.

20. Pecorelli N, Fiore JF, Kaneva P, Somasundram A, Charlebois P, Liberman AS, et al. An app for patient education and self-audit within an enhanced recovery program for bowel surgery: a pilot study assessing validity and usability. Surgical Endoscopy. 2018 2018;32(5):2263-73.

21. Petzel S, Vogel RI, Chan D, McClellan M, Gerber M, Cragg J, et al. Patient-centered ovarian cancer care: An interactive website to promote emotional quality of life for women and their caregivers. Psycho-Oncology. 2013 2013;22:131.

22. Saboga-Nunes L. Ehealth literacy (elisa) evaluation in Portugal and behavior change. Atencion Primaria. 2013 2013;45:47-8.

23. Salovey P, Williams-Piehota P, Mowad L, Moret ME, Edlund D, Andersen J. Bridging the digital divide by increasing computer and cancer literacy: community technology centers for head-start parents and families. J Health Commun. 2009 Apr-May;14(3):228-45. PMID: 19440907. doi: 10.1080/10810730902805804.

24. Samerski S, Muller H. [Digital health literacy in Germany - requested, but not supported? Results of the empirical study TK-DiSK]. Z Evid Fortbild Qual Gesundhwes. 2019 Aug;144-145:42-51. PMID: 31307911. doi: 10.1016/j.zefq.2019.05.006.

25. Spratling R, Spezia Faulkner M, Feinberg I, Hayat MJ. Creating opportunities for personal empowerment: Symptom and technology management resources (COPE-STAR) for caregivers of children who require medical technology. J Adv Nurs. 2020 Jan;76(1):347-55. PMID: 31612518. doi: 10.1111/jan.14235.

26. van der Vaart R, Drossaert CHC, Taal E, van de Laar MAFJ. Patient preferences for a hospital-based rheumatology interactive health communication application and factors associated with these preferences. Rheumatology. 2011 2011;50(9):1618-26.

### Digital intervention, not RCT, not assessing any type of literacy (n=44)

1. C. Everett Koop initiates online clinical trials info center for patients. Oncology (08909091). 2000 2000;14(2):288-90. PMID: 107044101.

2. Aikens JE, Trivedi R, Heapy A, Pfeiffer PN, Piette JD. Potential Impact of Incorporating a Patient-Selected Support Person into mHealth for Depression. Journal of General Internal Medicine. 2015 2015;30(6):797-803.

3. Alharbey R, Chatterjee S. An mHealth Assistive System "MyLung" to Empower Patients with Chronic Obstructive Pulmonary Disease: Design Science Research. JMIR Form Res. 2019 Mar 19;3(1):e12489. PMID: 30888329. doi: 10.2196/12489.

4. Alsem MW, van Meeteren KM, Verhoef M, Schmitz M, Jongmans MJ, Meily-Visser JMA, et al. Co-creation of a digital tool for the empowerment of parents of children with physical disabilities. Res Involv Engagem. 2017;3:26. PMID: 29238612. doi: 10.1186/s40900-017-0079-6.

5. Anderson MC. The medium is the messenger: using podcasting to deliver consumer health information. Journal of Consumer Health on the Internet. 2009 2009;13(2):119-28. PMID: 105368096.

6. Baldwin M, Spong A, Doward L, Gnanasakthy A. Patient-reported outcomes, patient-reported information: from randomized controlled trials to the social web and beyond. Patient. 2011;4(1):11-7. PMID: 21766890. doi: 10.2165/11585530-000000000-00000.

7. Barradell A, Houchen-Wolloff L, Clinch L, Orme M, Gardiner N, Singh S. Participant experiences of a digital selfmanagement programme for individuals hospitalised with an exacerbation of COPD. Thorax. 2018 2018;73:A134-A5.

8. Campbell JI, Aturinda I, Mwesigwa E, Burns B, Santorino D, Haberer JE, et al. The Technology Acceptance Model for Resource-Limited Settings (TAM-RLS): A Novel Framework for Mobile Health Interventions Targeted to Low-Literacy End-Users in Resource-Limited Settings. AIDS Behav. 2017 Nov;21(11):3129-40. PMID: 28421356. doi: 10.1007/s10461-017-1765-y.

9. Carolan-Olah M, Steele C, Krenzin G. Development and initial testing of a GDM information website for multi-ethnic women with GDM. BMC Pregnancy Childbirth. 2015 Jul 5;15(1):145. PMID: 26142482. doi: 10.1186/s12884-015-0578-0.

10. Cawdron R, Calder J, Issenman RM. e-Health? Clinical information network interest and impediments in a community paediatric setting. Paediatr Child Health. 2001 Dec;6(10):762-6. PMID: 20084152. doi: 10.1093/pch/6.10.762.

11. Chandra PS, Parameshwaran S, Satyanarayana VA, Varghese M, Liberti L, Duggal M, et al. I have no peace of mind—psychosocial distress expressed by rural women living with HIV in India as part of a mobile health intervention—a qualitative study. Archives of Women's Mental Health. 2018 2018;21(5):525-31. PMID: 131618937.

12. Coyne I, Prizeman G, Sheehan A, Malone H, While AE. An e-health intervention to support the transition of young people with long-term illnesses to adult healthcare services: Design and early use. Patient Educ Couns. 2016 Sep;99(9):1496-504. PMID: 27372524. doi: 10.1016/j.pec.2016.06.005.

13. Deren ME, DiGiovanni CW, Feller E. Web-based portrayal of platelet-rich plasma injections for orthopedic therapy. Clin J Sport Med. 2011 Sep;21(5):428-32. PMID: 21892016. doi: 10.1097/JSM.0B013E31822C629D.

14. Dodakian L, McKenzie A, Burke E, See J, Zhou R, Augsberger R, et al. A home-based telerehabilitation system for patients with stroke. Stroke. 2014 2014;45.

15. Fink A, Beck JC. Developing and Evaluating a Website to Guide Older Adults in Their Health Information Searches: A Mixed-Methods Approach. J Appl Gerontol. 2015 Aug;34(5):633-51. PMID: 24652883. doi: 10.1177/0733464813486961.

16. Gallagher JE, Dobrosielski-Vergona KA, Wingard RG, Williams TM. Web-based vs. traditional classroom instruction in gerontology: a pilot study. J Dent Hyg. 2005 Summer;79(3):7. PMID: 16197772.

17. Giordano A, Lugaresi A, Confalonieri P, Granella F, Radice D, Trojano M, et al. Implementation of the 'Sapere Migliora' information aid for newly diagnosed people with multiple sclerosis in routine clinical practice: a late-phase controlled trial. Multiple sclerosis (houndmills, basingstoke, england). 2014 2014;20(9):1234‐43. PMID: CN-01445200.

18. Girgis A, Durcinoska I, Arnold A, Kaadan N, Miller AA, Descallar J, et al. Phase III non-randomized controlled trial of PROMPT-Care, an eHealth intervention utilizing patient reported outcomes in routine clinical care: Impact on emergency department presentations. Journal of Clinical Oncology. 2019 2019;37.

19. Glynn SM, olph ET, Garrick T, Lui A. A proof of concept trial of an online psychoeducational program for relatives of both veterans and civilians living with schizophrenia. Psychiatric rehabilitation journal. 2010 2010;33(4):278‐87. PMID: CN-00742620.

20. Honda M, Matsumoto T, Hirose M. A system for health promotion using tele-homecare technology in community. Stud Health Technol Inform. 2013;192:940. PMID: 23920714.

21. Isrctn. InterSPACE: feasibility of an integrated telehealth and self-management programme for individuals hospitalised with an exacerbation of COPD. http://wwwwhoint/trialsearch/Trial2aspx?TrialID=ISRCTN13081008. 2015 2015. PMID: CN-01838855.

22. Jacobs RJ, Caballero J, Ownby RL, Kane MN. Development of a culturally appropriate computer-delivered tailored Internet-based health literacy intervention for Spanish-dominant Hispanics living with HIV. BMC Med Inform Decis Mak. 2014 Nov 30;14:103. PMID: 25433489. doi: 10.1186/s12911-014-0103-9.

23. Jacobsen HE. A comparison of on-campus first year undergraduate nursing students' experiences with face-to-face and on-line discussions. Nurse Educ Today. 2006 Aug;26(6):494-500. PMID: 16519969. doi: 10.1016/j.nedt.2006.01.005.

24. James WE, Ch, ler J, Brunner-Jackson B, Sox L, Swem M, et al. Acceptability and efficacy of a mhealth app for sarcoidosis associated fatigue. American Journal of Respiratory and Critical Care Medicine. 2019 2019;199(9).

25. Kowalski SD, Louis MA. Using computer technology to teach critical thinking. Nurse educator. 2000 2000;25(5):210‐2. PMID: CN-00556161.

26. Lind L, Karlsson D. Telehealth for "the digital illiterate"--elderly heart failure patients experiences. Stud Health Technol Inform. 2014;205:353-7. PMID: 25160205.

27. Marsac ML, Kassam-Adams N, Hildenbrand AK, Kohser KL, Winston FK. After the injury: initial evaluation of a web-based intervention for parents of injured children. Health Educ Res. 2011 Feb;26(1):1-12. PMID: 20858769. doi: 10.1093/her/cyq045.

28. Mateo KF, Berner NB, Ricci NL, Seekaew P, Sikerwar S, Tenner C, et al. Development of a 5As-based technology-assisted weight management intervention for veterans in primary care. BMC Health Serv Res. 2018 Jan 29;18(1):47. PMID: 29378584. doi: 10.1186/s12913-018-2834-2.

29. Mathiesen AS, Thomsen T, Jensen T, Schiotz C, Langberg H, Egerod I. The influence of diabetes distress on digital interventions for diabetes management in vulnerable people with type 2 diabetes: A qualitative study of patient perspectives. J Clin Transl Endocrinol. 2017 Sep;9:41-7. PMID: 29067269. doi: 10.1016/j.jcte.2017.07.002.

30. Nct. Online Support for Diabetes Self-Management. https://clinicaltrialsgov/show/NCT02504086. 2015 2015. PMID: CN-01491105.

31. O'Grady LA, Witteman H, Wathen CN. The experiential health information processing model: supporting collaborative web-based patient education. BMC Med Inform Decis Mak. 2008 Dec 16;8:58. PMID: 19087353. doi: 10.1186/1472-6947-8-58.

32. Pezzin LE, Laud P, Neuner J, Yen TW, Nattinger AB. A statewide controlled trial intervention to reduce use of unproven or ineffective breast cancer care. Contemp Clin Trials. 2016 Sep;50:150-6. PMID: 27521808. doi: 10.1016/j.cct.2016.08.005.

33. Poduval S, Ahmed S, Marston L, Hamilton F, Murray E. Crossing the Digital Divide in Online Self-Management Support: Analysis of Usage Data From HeLP-Diabetes. JMIR Diabetes. 2018 Dec 6;3(4):e10925. PMID: 30522988. doi: 10.2196/10925.

34. Poduval S, Marston L, Hamilton F, Stevenson F, Murray E. Feasibility, Acceptability, and Impact of a Web-Based Structured Education Program for Type 2 Diabetes: Real-World Study. JMIR Diabetes. 2020 Jan 6;5(1):e15744. PMID: 31904580. doi: 10.2196/15744.

35. Schultz PL, Carlisle R, Cheatham C, O'Grady M. Evaluating the Use of Plain Language in a Cancer Clinical Trial Website/App. J Cancer Educ. 2017 Dec;32(4):707-13. PMID: 26854082. doi: 10.1007/s13187-016-0994-5.

36. Toscos T, Daley C, Heral L, Doshi R, Chen YC, Eckert GJ, et al. Impact of electronic personal health record use on engagement and intermediate health outcomes among cardiac patients: a quasi-experimental study. J Am Med Inform Assoc. 2016 Jan;23(1):119-28. PMID: 26912538. doi: 10.1093/jamia/ocv164.

37. Vadheim LM, McPherson C, Kassner DR, Vanderwood KK, Hall TO, Butcher MK, et al. Adapted diabetes prevention program lifestyle intervention can be effectively delivered through telehealth. Diabetes Educ. 2010 Jul-Aug;36(4):651-6. PMID: 20534873. doi: 10.1177/0145721710372811.

38. van Deursen AJ. Internet skill-related problems in accessing online health information. Int J Med Inform. 2012 Jan;81(1):61-72. PMID: 22079240. doi: 10.1016/j.ijmedinf.2011.10.005.

39. van Middelaar T, Beishuizen CRL, Guillemont J, Barbera M, Richard E, Moll van Charante EP, et al. Engaging older people in an internet platform for cardiovascular risk self-management: a qualitative study among Dutch HATICE participants. BMJ Open. 2018 Jan 21;8(1):e019683. PMID: 29358447. doi: 10.1136/bmjopen-2017-019683.

40. Veinot TC, Campbell TR, Kruger DJ, Grodzinski A. A question of trust: user-centered design requirements for an informatics intervention to promote the sexual health of African-American youth. J Am Med Inform Assoc. 2013 Jul-Aug;20(4):758-65. PMID: 23512830. doi: 10.1136/amiajnl-2012-001361.

41. Weinert C, Cudney S, Hill W. Retention in a computer-based outreach intervention for chronically ill rural women. Appl Nurs Res. 2008 Feb;21(1):23-9. PMID: 18226760. doi: 10.1016/j.apnr.2006.08.002.

42. Weymann N, Harter M, Petrak F, Dirmaier J. Health information, behavior change, and decision support for patients with type 2 diabetes: development of a tailored, preference-sensitive health communication application. Patient Prefer Adherence. 2013 2013;7:1091-9. PMID: 24174871. doi: 10.2147/PPA.S46924.

43. Yager Z, O'Dea J. A controlled intervention to promote a healthy body image, reduce eating disorder risk and prevent excessive exercise among trainee health education and physical education teachers. Health Education Research. 2010 2010;25(5):841-52. PMID: 105102131.

44. Zaman T, Rife TL, Batki SL, Pennington DL. An electronic intervention to improve safety for pain patients co-prescribed chronic opioids and benzodiazepines. Subst Abus. 2018;39(4):441-8. PMID: 29595408. doi: 10.1080/08897077.2018.1455163.

### Relevant systematic reviews on DHI or health literacy (n=2)

1. Carter MC, Burley VJ, Cade JE. Handheld Electronic Technology for Weight Loss in Overweight/Obese Adults. Curr Obes Rep. 2014 Sep;3(3):307-15. PMID: 26626760. doi: 10.1007/s13679-014-0112-0.

2. Jacobs RJ, Lou JQ, Ownby RL, Caballero J. A systematic review of eHealth interventions to improve health literacy. Health Informatics J. 2016 Jun;22(2):81-98. PMID: 24916567. doi: 10.1177/1460458214534092.

## No relevant outcome assessed or reported: DHI, RCTs, not assessing any type of literacy (n=193)

1. Actrn. BetaMe. An innovative management of diabetes and prediabetes with a comprehensive digital health programme: a randomised controlled trial. http://wwwwhoint/trialsearch/Trial2aspx?TrialID=ACTRN12617000549325. 2017 2017. PMID: CN-01814325.

2. Actrn. Enhancing decision-making about treatment in bipolar II disorder: evaluation of a treatment decision-aid for patients and their family. http://wwwwhoint/trialsearch/Trial2aspx?TrialID=ACTRN12617000840381. 2017 2017. PMID: CN-01892315.

3. Actrn. Effectiveness of Using Social Media in a Family-based Intervention for Childhood Obesity. http://wwwwhoint/trialsearch/Trial2aspx?TrialID=ACTRN12617000844347. 2017 2017. PMID: CN-01894983.

4. Actrn. Evaluation of the effectiveness of an interactive avatar-based education application for improving heart failure patients’ knowledge and self-care behaviours: a pragmatic randomised controlled trial. http://wwwwhoint/trialsearch/Trial2aspx?TrialID=ACTRN12617001403325. 2017 2017. PMID: CN-01885983.

5. Actrn. Helping People to Choose Wisely: evaluating Question Based Interventions to Support Shared Decision Making in Healthcare. http://wwwwhoint/trialsearch/Trial2aspx?TrialID=ACTRN12618002014235. 2018 2018. PMID: CN-01949007.

6. Actrn. Decision support for colorectal cancer prevention - what is the best way to present access to online information: frequently Asked Questions (FAQs) targeted to age and gender or a generic information topic list? http://wwwwhoint/trialsearch/Trial2aspx?TrialID=ACTRN12618000137291. 2018 2018. PMID: CN-01896750.

7. Advocat J, Lindsay J. Internet-based trials and the creation of health consumers. Soc Sci Med. 2010 Feb;70(3):485-92. PMID: 19926185. doi: 10.1016/j.socscimed.2009.10.051.

8. Al Ghamdi E, Yunus F, Da'ar O, El-Metwally A, Khalifa M, Aldossari B, et al. The Effect of Screen Size on Mobile Phone User Comprehension of Health Information and Application Structure: An Experimental Approach. J Med Syst. 2016 Jan;40(1):11. PMID: 26573648. doi: 10.1007/s10916-015-0381-5.

9. Alexander GL, McClure JB, Calvi JH, Divine GW, Stopponi MA, Rolnick SJ, et al. A randomized clinical trial evaluating online interventions to improve fruit and vegetable consumption. American journal of public health. 2010 2010;100(2):319‐26. PMID: CN-00732612.

10. Anstey KJ, Bahar-Fuchs A, Herath P, Rebok GW, Cherbuin N. A 12-week multidomain intervention versus active control to reduce risk of Alzheimer's disease: study protocol for a randomized controlled trial. Trials. 2013 Feb 27;14:60. PMID: 23442574. doi: 10.1186/1745-6215-14-60.

11. Atkinson NL, Massett HA, Mylks C, McCormack LA, Kish-Doto J, Hesse BW, et al. Assessing the impact of user-centered research on a clinical trial eHealth tool via counterbalanced research design. J Am Med Inform Assoc. 2011 Jan-Feb;18(1):24-31. PMID: 21169619. doi: 10.1136/jamia.2010.006122.

12. Atreja A, Khan S, Otobo E, Webb L, Rogers J, Ullman T, et al. Impact of real world home-based remote monitoring on quality of care and quality of life in IBD patients: Interim results of pragmatic randomized trial. Gastroenterology. 2017 2017;152(5):S600-S1.

13. Atreja A, Szigethy E, Otobo E, Chang HL, Keefer L, Rogers J, et al. Improved Quality of Care and Quality of Life for IBD Patients Using Healthpromise App: A Randomized Control Trial. Gastroenterology. 2018 2018;154(6):S-6.

14. Bailey SC, Paasche-Orlow MK, Adams WG, Brokenshire SA, Hedlund LA, Hickson RP, et al. The electronic medication complete communication (EMC(2)) study: Rationale and methods for a randomized controlled trial of a strategy to promote medication safety in ambulatory care. Contemp Clin Trials. 2016 Nov;51:72-7. PMID: 27777127. doi: 10.1016/j.cct.2016.10.005.

15. Bailey SC, Wismer GA, Parker RM, Walton SM, Wood AJJ, Wallia A, et al. Development and rationale for a multifactorial, randomized controlled trial to test strategies to promote adherence to complex drug regimens among older adults. Contemp Clin Trials. 2017 Nov;62:21-6. PMID: 28823927. doi: 10.1016/j.cct.2017.08.013.

16. Bajaj HS, Venn K, Ye C, Aronson R. Randomized Trial of Long-Acting Insulin Glargine Titration Web Tool (LTHome) Versus Enhanced Usual Therapy of Glargine Titration (INNOVATE Trial). Diabetes Technol Ther. 2016 Oct;18(10):610-5. PMID: 27652718. doi: 10.1089/dia.2016.0182.

17. Baker DW, Dewalt DA, Schillinger D, Hawk V, Ruo B, Bibbins-Domingo K, et al. The effect of progressive, reinforcing telephone education and counseling versus brief educational intervention on knowledge, self-care behaviors and heart failure symptoms. J Card Fail. 2011 Oct;17(10):789-96. PMID: 21962415. doi: 10.1016/j.cardfail.2011.06.374.

18. Barahimi H, Zolfaghari M, Abolhassani F, Rahimi Foroushani A, Mohammadi A, Rajaee F. E-Learning Model in Chronic Kidney Disease Management: a Controlled Clinical Trial. Iran J Kidney Dis. 2017 Jul;11(4):280-5. PMID: 28794290.

19. Barnard KD, Blatch-Jones A. Web-Based Management Trial of Diabetes Care. Diabetes Technol Ther. 2016 Oct;18(10):605-6. PMID: 27749115. doi: 10.1089/dia.2016.0320.

20. Beaujean DJ, Gassner F, Wong A, Steenbergen JE, Crutzen R, Ruwaard D. Education on tick bite and Lyme borreliosis prevention, aimed at schoolchildren in the Netherlands: comparing the effects of an online educational video game versus a leaflet or no intervention. BMC Public Health. 2016 Nov 16;16(1):1163. PMID: 27852247. doi: 10.1186/s12889-016-3811-5.

21. Berry D, Yeh Y, Halpenny B. Symptom distress by education levels in ambulatory patients at a comprehensive cancer center. Psycho-Oncology. 2012 2012;21:84.

22. Bickmore TW, Utami D, Matsuyama R, Paasche-Orlow MK. Improving Access to Online Health Information With Conversational Agents: A Randomized Controlled Experiment. J Med Internet Res. 2016 Jan 4;18(1):e1. PMID: 26728964. doi: 10.2196/jmir.5239.

23. Block RC, Abdolahi A, Niemiec CP, Rigby CS, Williams GC. Effects of an evidence-based computerized virtual clinician on low-density lipoprotein and non-high-density lipoprotein cholesterol in adults without cardiovascular disease: The Interactive Cholesterol Advisory Tool. Health Informatics Journal. 2016 2016;22(4):897-910. PMID: 119546245. Language: English. Entry Date: 20161130. Revision Date: 20180530. Publication Type: Article.

24. Bosworth HB, Olsen MK, McCant F, Stechuchak KM, Danus S, Crowley MJ, et al. Telemedicine cardiovascular risk reduction in veterans: The CITIES trial. Am Heart J. 2018 May;199:122-9. PMID: 29754649. doi: 10.1016/j.ahj.2018.02.002.

25. Carmel AS, Cornelius-Schecter A, Frankel B, Jannat-Khah D, Sinha S, Pelzman F, et al. Evaluation of the Patient Activated Learning System (PALS) to improve knowledge acquisition, retention, and medication decision making among hypertensive adults: Results of a pilot randomized controlled trial. Patient Educ Couns. 2019 Aug;102(8):1467-74. PMID: 30928344. doi: 10.1016/j.pec.2019.03.001.

26. Chi CI. Internet-based mindfulness and rumination-focused cognitive behavioural therapy as selective prevention of anxiety and depression: a randomised controlled trial. http://wwwwhoint/trialsearch/Trial2aspx?TrialID=ChiCTR-IOR-15006470. 2015 2015. PMID: CN-01883204.

27. Chi CI. 21 day mindfulness navigation intervention. http://wwwwhoint/trialsearch/Trial2aspx?TrialID=ChiCTR-INR-17011880. 2017 2017. PMID: CN-01892111.

28. Chi CT. HealthyLiving – an Internet-based mental health program. http://wwwwhoint/trialsearch/Trial2aspx?TrialID=ChiCTR-TRC-12002623. 2012 2012. PMID: CN-01817192.

29. Chu A, Huber J, Mastel-Smith B, Cesario S. 'Partnering with seniors for better health': computer use and internet health information retrieval among older adults in a low socioeconomic community. Journal of the Medical Library Association. 2009 2009;97(1):12-20. PMID: 105632239. Language: English. Entry Date: 20090626. Revision Date: 20150820. Publication Type: Journal Article.

30. Chu A, Mastel-Smith B. The outcomes of anxiety, confidence, and self-efficacy with Internet health information retrieval in older adults: a pilot study. Comput Inform Nurs. 2010 Jul-Aug;28(4):222-8. PMID: 20571374. doi: 10.1097/NCN.0b013e3181e1e271.

31. Chu JTW, Whittaker R, Jiang Y, Wadham A, Stasiak K, Shepherd M, et al. Evaluation of MyTeen - a SMS-based mobile intervention for parents of adolescents: a randomised controlled trial protocol. BMC Public Health. 2018 Oct 26;18(1):1203. PMID: 30367613. doi: 10.1186/s12889-018-6132-z.

32. Coley N, Rosenberg A, van Middelaar T, Soulier A, Barbera M, Guillemont J, et al. Older Adults' Reasons for Participating in an eHealth Prevention Trial: A Cross-Country, Mixed-Methods Comparison. J Am Med Dir Assoc. 2019 Jul;20(7):843-9 e5. PMID: 30541689. doi: 10.1016/j.jamda.2018.10.019.

33. Cortese J, Lustria MLA. Can tailoring increase elaboration of health messages delivered via an adaptive educational site on adolescent sexual health and decision making? Journal of the American Society for Information Science & Technology. 2012 2012;63(8):1567-80. PMID: 104482094. Language: English. Entry Date: 20120801. Revision Date: 20150820. Publication Type: Journal Article.

34. Coughlin SS, Besenyi GM, Bowen D, De Leo G. Development of the Physical activity and Your Nutrition for Cancer (PYNC) smartphone app for preventing breast cancer in women. Mhealth. 2017;3:5. PMID: 28293621. doi: 10.21037/mhealth.2017.02.02.

35. Cowdery J, Majeske P, Frank R, Brown D. Exergame Apps and Physical Activity: The Results of the ZOMBIE Trial. American Journal of Health Education. 2015 2015;46(4):216-22. PMID: 109815555. Language: English. Entry Date: 20150714. Revision Date: 20150923. Publication Type: Journal Article.

36. Crowley MJ, Powers BJ, Olsen MK, Grubber JM, Koropchak C, Rose CM, et al. The Cholesterol, Hypertension, And Glucose Education (CHANGE) study: results from a randomized controlled trial in African Americans with diabetes. Am Heart J. 2013 Jul;166(1):179-86. PMID: 23816038. doi: 10.1016/j.ahj.2013.04.004.

37. Crutzen R, Cyr D, de Vries NK. The role of user control in adherence to and knowledge gained from a website: randomized comparison between a tunneled version and a freedom-of-choice version. Journal of medical internet research. 2012 2012;14(2):e45. PMID: CN-00832226.

38. Cudney S, Weinert C. An online approach to providing chronic illness self-management information. Comput Inform Nurs. 2012 Feb;30(2):110-7. PMID: 21915043. doi: 10.1097/NCN.0b013e31822b899a.

39. D'Alessandro DM, Kreiter CD, Kinzer SL, Peterson MW. A randomized controlled trial of an information prescription for pediatric patient education on the Internet. Arch Pediatr Adolesc Med. 2004 Sep;158(9):857-62. PMID: 15351750. doi: 10.1001/archpedi.158.9.857.

40. Dadds MR, Thai C, Mendoza Diaz A, Broderick J, Moul C, Tully LA, et al. Therapist-assisted online treatment for child conduct problems in rural and urban families: Two randomized controlled trials. J Consult Clin Psychol. 2019 Aug;87(8):706-19. PMID: 31204839. doi: 10.1037/ccp0000419.

41. Danila M, Rahn E, Outman R, Mudano A, Thomas T, Allison J, et al. Factors associated with the online uptake of a multi-modal educational intervention for the activating patients at risk for osteoporosis (APROPOS) study: A randomized trial within the glow cohort. Journal of Bone and Mineral Research. 2017 2017;31.

42. Davies EB, Beever E, Glazebrook C. A pilot randomised controlled study of the mental health first aid eLearning course with UK medical students. BMC Med Educ. 2018 Mar 21;18(1):45. PMID: 29562906. doi: 10.1186/s12909-018-1154-x.

43. Del Salto G, Patel D, Curtis LM, Parker R, Rachman F, Rittner SS, et al. An electronic health record-enabled (EHR) universal medication schedule to promote adherence: A pragmatic trial. Journal of General Internal Medicine. 2017 2017;32(2):S113.

44. Drks. Pro-HEAD - Promoting Help-seeking using E-technology for ADolescents. Subproject 5: mental Health Promotion: universal School-Based Prevention for Healthy Adolescents Using Online or Face-to-Face Interventions. http://wwwwhoint/trialsearch/Trial2aspx?TrialID=DRKS00014693. 2018 2018. PMID: CN-01899322.

45. DuBenske LL, Gustafson DH, Namkoong K, Hawkins RP, Atwood AK, Brown RL, et al. CHESS improves cancer caregivers' burden and mood: results of an eHealth RCT. Health Psychol. 2014 Oct;33(10):1261-72. PMID: 24245838. doi: 10.1037/a0034216.

46. Duggleby W, Ploeg J, McAiney C, Fisher K, Jovel Ruiz K, Ghosh S, et al. A Comparison of Users and Nonusers of a Web-Based Intervention for Carers of Older Persons With Alzheimer Disease and Related Dementias: Mixed Methods Secondary Analysis. J Med Internet Res. 2019 Oct 17;21(10):e14254. PMID: 31625947. doi: 10.2196/14254.

47. Dunn ME, Fried-Somerstein A, Flori JN, Hall TV, Dvorak RD. Reducing Alcohol Use in Mandated College Students: A Comparison of a Brief Motivational Intervention (BMI) and the Expectancy Challenge Alcohol Literacy Curriculum (ECALC). Experimental and Clinical Psychopharmacology. 2019 2019.

48. Ekeland AG, Grottland A. Assessment of Mast in European Patient-Centered Telemedicine Pilots. Int J Technol Assess Health Care. 2015 Jan;31(5):304-11. PMID: 26679197. doi: 10.1017/S0266462315000574.

49. Elbert SP, Dijkstra A, Oenema A. A Mobile Phone App Intervention Targeting Fruit and Vegetable Consumption: The Efficacy of Textual and Auditory Tailored Health Information Tested in a Randomized Controlled Trial. J Med Internet Res. 2016 Jun 10;18(6):e147. PMID: 27287823. doi: 10.2196/jmir.5056.

50. Elliott MJ, Straus SE, Pannu N, Ahmed SB, Laupacis A, Chong GC, et al. A randomized controlled trial comparing in-person and wiki-inspired nominal group techniques for engaging stakeholders in chronic kidney disease research prioritization. BMC Med Inform Decis Mak. 2016 Aug 24;16:113. PMID: 27553026. doi: 10.1186/s12911-016-0351-y.

51. Eschenbeck H, Lehner L, Hofmann H, Bauer S, Becker K, Diestelkamp S, et al. School-based mental health promotion in children and adolescents with StresSOS using online or face-to-face interventions: study protocol for a randomized controlled trial within the ProHEAD Consortium. Trials. 2019 2019;20(1):N.PAG-N.PAG. PMID: 134181738. Language: English. Entry Date: In Process. Revision Date: 20190605. Publication Type: journal article. Journal Subset: Biomedical.

52. Etter J, le Houezec J, feldt B. Impact of messages on concomitant use of nicotine replacement therapy and cigarettes: a randomized trial on the Internet. Addiction. 2003 2003;98(7):941-50. PMID: 106686870. Language: English. Entry Date: 20050425. Revision Date: 20150711. Publication Type: Journal Article.

53. Fields J, Cemballi A, Michalec C, Uchida D, DeSmidt H, Cuellar J, et al. In-home technology training to reduce social isolation and improve tablet use among older adults: Findings from the tech allies program. Journal of Applied Gerontology. 2020 2019:1-11. doi: 10.1177/0733464820910028.

54. Finkelstein J, Bedra M. Is Internet search better than structured instruction for web-based health education? Studies in health technology and informatics. 2013 2013;190:65‐7. PMID: CN-00996839.

55. Fleming JN, Treiber F, McGillicuddy J, Gebregziabher M, Taber DJ. Improving Transplant Medication Safety Through a Pharmacist-Empowered, Patient-Centered, mHealth-Based Intervention: TRANSAFE Rx Study Protocol. JMIR Res Protoc. 2018 Mar 2;7(3):e59. PMID: 29500161. doi: 10.2196/resprot.9078.

56. Freeman E, Barker C, Pistrang N. Outcome of an online mutual support group for college students with psychological problems. Cyberpsychol Behav. 2008 Oct;11(5):591-3. PMID: 18817485. doi: 10.1089/cpb.2007.0133.

57. Gagnon MP, Ndiaye MA, Larouche A, Chabot G, Chabot C, Buyl R, et al. Optimising patient active role with a user-centred eHealth platform (CONCERTO+) in chronic diseases management: a study protocol for a pilot cluster randomised controlled trial. BMJ Open. 2019 Apr 2;9(4):e028554. PMID: 30944143. doi: 10.1136/bmjopen-2018-028554.

58. Gayed A, Bryan BT, Petrie K, Deady M, Milner A, LaMontagne AD, et al. A protocol for the HeadCoach trial: the development and evaluation of an online mental health training program for workplace managers. BMC Psychiatry. 2018 Jan 29;18(1):25. PMID: 29378536. doi: 10.1186/s12888-018-1603-4.

59. Genz J, Haastert B, Muller H, Verheyen F, Cole D, Rathmann W, et al. [Prevention of Type 2 Diabetes: Evidence-Based Patient Information--A Randomised Controlled Trial]. Gesundheitswesen. 2015 Sep;77 Suppl 1:S91-2. PMID: 23549653. doi: 10.1055/s-0032-1329998.

60. Glaser E, Richard C, Lussier MT. The impact of a patient web communication intervention on reaching treatment suggested guidelines for chronic diseases: A randomized controlled trial. Patient Educ Couns. 2017 Nov;100(11):2062-70. PMID: 28535926. doi: 10.1016/j.pec.2017.05.022.

61. Goe R, Ipsen C, Bliss S. Pilot Testing a Digital Career Literacy Training for Vocational Rehabilitation Professionals. Rehabilitation Counseling Bulletin. 2018 2018;61(4):236-43. PMID: 129952269. Language: English. Entry Date: 20180608. Revision Date: 20180613. Publication Type: Article.

62. Gordon EJ, Sohn MW, Chang CH, McNatt G, Vera K, Beauvais N, et al. Effect of a Mobile Web App on Kidney Transplant Candidates' Knowledge About Increased Risk Donor Kidneys: a Randomized Controlled Trial. Transplantation. 2017 2017;101(6):1167‐76. PMID: CN-01411244.

63. Graumlich JF, Wang H, Madison A, Wolf MS, Kaiser D, Dahal K, et al. Effects of a Patient-Provider, Collaborative, Medication-Planning Tool: A Randomized, Controlled Trial. J Diabetes Res. 2016 2016;2016:2129838. PMID: 27699179. doi: 10.1155/2016/2129838.

64. Griffiths KM, Walker J, Batterham PJ. Help seeking for social anxiety: A pilot randomised controlled trial. Digit Health. 2017 Jan-Dec;3:2055207617712047. PMID: 29942603. doi: 10.1177/2055207617712047.

65. Gulliver A, Griffiths KM, Christensen H, Mackinnon A, Calear AL, Parsons A, et al. Internet-based interventions to promote mental health help-seeking in elite athletes: an exploratory randomized controlled trial. J Med Internet Res. 2012 Jun 29;14(3):e69. PMID: 22743352. doi: 10.2196/jmir.1864.

66. Haesum LKE, Ehlers LH, Hejlesen OK. The long-term effects of using telehomecare technology on functional health literacy: results from a randomized trial. Public Health. 2017 Sep;150:43-50. PMID: 28623766. doi: 10.1016/j.puhe.2017.05.002.

67. Hartman S, Dunsiger S, Bock B, Larsen B, Linke S, Pekmezi D, et al. Physical activity maintenance among Spanish-speaking Latinas in a randomized controlled trial of an Internet-based intervention. Journal of Behavioral Medicine. 2017 2017;40(3):392-402. PMID: 122710182. Language: English. Entry Date: 20170501. Revision Date: 20190308. Publication Type: Article.

68. Hawkins RP, Pingree S, Shaw B, Serlin RC, Swoboda C, Han JY, et al. Mediating processes of two communication interventions for breast cancer patients. Patient Educ Couns. 2010 Dec;81 Suppl:S48-53. PMID: 21081261. doi: 10.1016/j.pec.2010.10.021.

69. Hedlund LA, Curtis LM, Moore A, Oramasionwu C, Mc- Carthy DM, Adams B, et al. A medication risk communication and surveillance strategy for primary care: Introducing the EMC2 trial. Journal of General Internal Medicine. 2016 2016;31(2):S857.

70. Heitkemper E, Mamykina L, Cassells A, Tobin J, Smaldone A. Characteristics of underserved adults enrolled in the mobile diabetes detective (MoDD) randomized controlled trial. Diabetes. 2017 2017;66:A188-A9.

71. Heitkemper EM, Mamykina L, Tobin JN, Cassells A, Smaldone A. Baseline Characteristics and Technology Training of Underserved Adults With Type 2 Diabetes in the Mobile Diabetes Detective (MoDD) Randomized Controlled Trial. Diabetes Educ. 2017 Dec;43(6):576-88. PMID: 29059017. doi: 10.1177/0145721717737367.

72. Henney S, Irving R. Prospective, randomised, controlled trial comparing delivery of patient information for functional endoscopic sinus surgery via website versus printed leaflet. J Laryngol Otol. 2014 Mar;128(3):249-54; quiz 54. PMID: 24666802. doi: 10.1017/S0022215114000449.

73. Herring SJ, Albert JJ, Darden N, Bailer B, Cruice J, Hassan S, et al. Targeting pregnancy-related weight gain to reduce disparities in obesity: Baseline results from the Healthy Babies trial. Contemp Clin Trials. 2019 Dec;87:105822. PMID: 31400513. doi: 10.1016/j.cct.2019.105822.

74. Hickey KT, Hauser NR, Valente LE, Riga TC, Frulla AP, Masterson Creber R, et al. A single-center randomized, controlled trial investigating the efficacy of a mHealth ECG technology intervention to improve the detection of atrial fibrillation: the iHEART study protocol. BMC Cardiovasc Disord. 2016 Jul 16;16(1):152. PMID: 27422639. doi: 10.1186/s12872-016-0327-y.

75. Hill W, Weinert C, Cudney S. Influence of a computer intervention on the psychological status of chronically ill rural women: preliminary results. Nurs Res. 2006 Jan-Feb;55(1):34-42. PMID: 16439927. doi: 10.1097/00006199-200601000-00005.

76. Hudaya I. Smartphone application to support self-management of abortion in Indonesia. International Journal of Gynecology and Obstetrics. 2018 2018;143:53-4.

77. Hudson JL, Moss-Morris R, Norton S, Picariello F, Game D, Carroll A, et al. Tailored online cognitive behavioural therapy with or without therapist support calls to target psychological distress in adults receiving haemodialysis: a feasibility randomised controlled trial. Journal of psychosomatic research. 2017 2017;102:61‐70. PMID: CN-01423168.

78. Imamura K, Kawakami N, Tsuno K, Tsuchiya M, Shimada K, Namba K. Effects of web-based stress and depression literacy intervention on improving symptoms and knowledge of depression among workers: A randomized controlled trial. J Affect Disord. 2016 Oct;203:30-7. PMID: 27280960. doi: 10.1016/j.jad.2016.05.045.

79. Imamura K, Kawakami N, Tsuno K, Tsuchiya M, Shimada K, Namba K, et al. Effects of web-based stress and depression literacy intervention on improving work engagement among workers with low work engagement: An analysis of secondary outcome of a randomized controlled trial. J Occup Health. 2017 Jan 24;59(1):46-54. PMID: 27885247. doi: 10.1539/joh.16-0187-OA.

80. Irct138901212621N. The Effect of Electronic Education on Predisposing Factors and Metabolic Control Indicators of Diabetic Patients on Insulin. http://wwwwhoint/trialsearch/Trial2aspx?TrialID=IRCT138901212621N5. 2011 2011. PMID: CN-01854131.

81. Irct2013082914522N. The Study on the Effect of Web-based Iranian Diabetic Personal Health Record System. http://wwwwhoint/trialsearch/Trial2aspx?TrialID=IRCT2013082914522N1. 2015 2015. PMID: CN-01858500.

82. Irct2015091423996N. e-learning Chronic Kidney Disease Interventional Study. http://wwwwhoint/trialsearch/Trial2aspx?TrialID=IRCT2015091423996N2. 2015 2015. PMID: CN-01856835.

83. Irct20160808029255N. The effect of virtual education to promote of sexual function, genital self â€“ image and sexual distress in women with Rokitansky syndrome. http://wwwwhoint/trialsearch/Trial2aspx?TrialID=IRCT20160808029255N7. 2019 2019. PMID: CN-01974799.

84. Isrctn. Working memory training for children who have survived a brain injury. http://wwwwhoint/trialsearch/Trial2aspx?TrialID=ISRCTN01872582. 2013 2013. PMID: CN-01854182.

85. Isrctn. The mental health impact of computer and internet training on a mainly non-white, depressed sample of non-institutionalized older adults. http://wwwwhoint/trialsearch/Trial2aspx?TrialID=ISRCTN27927877. 2013 2013. PMID: CN-01864900.

86. Isrctn. Measuring the effectiveness of brief web-based acceptance and commitment therapy to improve psychological well-being in college students. http://wwwwhoint/trialsearch/Trial2aspx?TrialID=ISRCTN12086191. 2016 2016. PMID: CN-01862863.

87. Jack B, Bickmore T, Hempstead M, Yinusa-Nyahkoon L, Sadikova E, Mitchell S, et al. Reducing preconception risks among African American women with conversational agent technology. Journal of the American Board of Family Medicine. 2015 2015;28(4):441-51.

88. Jalota L, Aryal MR, Mahmood M, Wasser T, Donato A. Interventions to increase physician efficiency and comfort with an electronic health record system. Methods Inf Med. 2015 2015;54(1):103-9. PMID: 25377629. doi: 10.3414/ME14-01-0047.

89. Kantarcigil C, Malandraki GA. First Step in Telehealth Assessment: A Randomized Controlled Trial to Investigate the Effectiveness of an Electronic Case History Form for Dysphagia. Dysphagia. 2017 Aug;32(4):548-58. PMID: 28424897. doi: 10.1007/s00455-017-9798-y.

90. Kim S, Werner P, Richardson A, Anstey KJ. Dementia Stigma Reduction (DESeRvE): Study protocol for a randomized controlled trial of an online intervention program to reduce dementia-related public stigma. Contemp Clin Trials Commun. 2019 Jun;14:100351. PMID: 30997434. doi: 10.1016/j.conctc.2019.100351.

91. Kong EH, Song E, Evans LK. Effects of a Multicomponent Restraint Reduction Program for Korean Nursing Home Staff. J Nurs Scholarsh. 2017 May;49(3):325-35. PMID: 28384390. doi: 10.1111/jnu.12296.

92. Krishnamurti L, Ross D, Sinha C, Leong T, Bakshi N, Mittal N, et al. Comparative Effectiveness of a Web-Based Patient Decision Aid for Therapeutic Options for Sickle Cell Disease: Randomized Controlled Trial. J Med Internet Res. 2019 Dec 4;21(12):e14462. PMID: 31799940. doi: 10.2196/14462.

93. Lalor JP, Woolf B, Yu H. Improving Electronic Health Record Note Comprehension With NoteAid: Randomized Trial of Electronic Health Record Note Comprehension Interventions With Crowdsourced Workers. J Med Internet Res. 2019 Jan 16;21(1):e10793. PMID: 30664453. doi: 10.2196/10793.

94. Lee S, Odenthal K, Radovic A. 184. Identifying Risk Factors for Depressed and Anxious Adolescents with Low Perceived Need for Treatment. Journal of Adolescent Health. 2020 2020;66(2):S93-S4.

95. Lee TJ, Cameron LD, Wunsche B, Stevens C. A randomized trial of computer-based communications using imagery and text information to alter representations of heart disease risk and motivate protective behaviour. Br J Health Psychol. 2011 Feb;16(Pt 1):72-91. PMID: 21226785. doi: 10.1348/135910710X511709.

96. Lopez-Olivo MA, Barbo A, Rizvi T, Volk R, Lin H, Suarez-Almazor ME. Improving outcomes with a multimedia patient education tool in patients with osteoporosis after 6 months. A randomized controlled trial. Arthritis and rheumatology. 2015 2015;67. PMID: CN-01126736.

97. Lopez-Olivo MA, Barbo A, Rizvi T, Volk R, Lin H, Suarez-Almazor ME. A randomized controlled trial to evaluate a multimedia patient education tool in patients with knee osteoarthritis. Six-month results. Arthritis and rheumatology. 2015 2015;67. PMID: CN-01126737.

98. Lubman DI, Grigg J, Manning V, Hall K, Volpe I, Dias S, et al. A structured telephone-delivered intervention to reduce problem alcohol use (Ready2Change): study protocol for a parallel group randomised controlled trial. Trials. 2019 2019;20(1). PMID: CN-01986282.

99. Maidment D, Brassington W, Wharrad H, Ferguson M. Internet Competency Predicts Practical Hearing Aid Knowledge and Skills in First-Time Hearing Aid Users. Am J Audiol. 2016 Oct 1;25(3S):303-7. PMID: 27768193. doi: 10.1044/2016_AJA-16-0022.

100. Mattheos N, Nattestad A, Christersson C, Jansson H, Attstrom R. The effects of an interactive software application on the self-assessment ability of dental students. Eur J Dent Educ. 2004 Aug;8(3):97-104. PMID: 15233773. doi: 10.1111/j.1600-0579.2004.00325.x.

101. Mc Master M, Clare L, Kim S, Torres S, Anstey KJ. A protocol for a randomised controlled trial of multidomain dementia risk reduction for mild cognitive impairment. Alzheimer's and Dementia. 2017 2017;13(7):P1201.

102. McCloud RF, Okechukwu CA, Sorensen G, Viswanath K. Entertainment or Health? Exploring the Internet Usage Patterns of the Urban Poor: A Secondary Analysis of a Randomized Controlled Trial. J Med Internet Res. 2016 Mar 3;18(3):e46. PMID: 26940637. doi: 10.2196/jmir.4375.

103. McMaster M, Kim S, Clare L, Torres SJ, D'Este C, Anstey KJ. Body, Brain, Life for Cognitive Decline (BBL-CD): protocol for a multidomain dementia risk reduction randomized controlled trial for subjective cognitive decline and mild cognitive impairment. Clin Interv Aging. 2018 2018;13:2397-406. PMID: 30538436. doi: 10.2147/CIA.S182046.

104. Miller Jr DP, Spangler JG, Case LD, Goff Jr DC, Singh S, Pignone MP. Effectiveness of a web-based colorectal cancer screening patient decision aid: A randomized controlled trial in a mixed-literacy population. American Journal of Preventive Medicine. 2011 2011;40(6):608-15.

105. Morrow DG, Conner-Garcia T, Graumlich JF, Wolf MS, McKeever S, Madison A, et al. An EMR-based tool to support collaborative planning for medication use among adults with diabetes: design of a multi-site randomized control trial. Contemporary clinical trials. 2012 2012;33(5):1023‐32. PMID: CN-00854079.

106. Muller I, Rowsell A, Stuart B, Hayter V, Little P, Ganahl K, et al. Effects on Engagement and Health Literacy Outcomes of Web-Based Materials Promoting Physical Activity in People With Diabetes: An International Randomized Trial. J Med Internet Res. 2017 Jan 23;19(1):e21. PMID: 28115299. doi: 10.2196/jmir.6601.

107. Muscat DM, Chang EH, Thompson R, Cvejic E, Tracy M, Zadro J, et al. Evaluation of the Choosing Wisely Australia 5 Questions resource and a shared decision-making preparation video: protocol for an online experiment. BMJ Open. 2019 Nov 14;9(11):e033126. PMID: 31727667. doi: 10.1136/bmjopen-2019-033126.

108. Nahm ES, Resnick B, Brown C, Zhu S, Magaziner J, Bellantoni M, et al. The Effects of an Online Theory-Based Bone Health Program for Older Adults. J Appl Gerontol. 2017 Sep;36(9):1117-44. PMID: 26675352. doi: 10.1177/0733464815617284.

109. Nct. Use of Telehealth In-home Messaging to Improve GI (Gastrointestinal) Endoscopy Completion Rates. https://clinicaltrialsgov/show/NCT00310362. 2006 2006. PMID: CN-01481509.

110. Nct. Using Effective Provider-Patient Communication to Improve Cancer Screening Among Low Literacy Patients. https://clinicaltrialsgov/show/NCT01361035. 2011 2011. PMID: CN-01486689.

111. Nct. Evaluation of DVD and Internet Decision Aids for Hip and Knee Osteoarthritis: focus on Health Literacy. https://clinicaltrialsgov/show/NCT01618097. 2012 2012. PMID: CN-01503973.

112. Nct. Scale-up of an Internet-Delivered Study for HIV+ Men. https://clinicaltrialsgov/show/NCT02023580. 2013 2013. PMID: CN-01480058.

113. Nct. Music for Health Project. https://clinicaltrialsgov/show/NCT01786148. 2013 2013. PMID: CN-01540354.

114. Nct. Health Literacy Intervention for Informed Consent of Cancer Patients Considering Clinical Trial Participation. https://clinicaltrialsgov/show/NCT01964222. 2013 2013. PMID: CN-01536817.

115. Nct. Prevention of the Metabolic Syndrome by New Lifestyle Intervention Methods. https://clinicaltrialsgov/show/NCT01959763. 2013 2013. PMID: CN-01536709.

116. Nct. Dissemination of a Theory-Based Bone Health Program in Online. https://clinicaltrialsgov/show/NCT01963169. 2013 2013. PMID: CN-01536791.

117. Nct. Relational Agent for Alcohol Screening and Treatment. https://clinicaltrialsgov/show/NCT02030288. 2014 2014. PMID: CN-01480257.

118. Nct. Using Virtual Counselors to Overcome Genetic Literacy Barriers. https://clinicaltrialsgov/show/NCT02344433. 2015 2015. PMID: CN-01551662.

119. Nct. Verizon mHealth Solution for Patients With Peripheral Artery Disease (PAD). https://clinicaltrialsgov/show/NCT02472561. 2015 2015. PMID: CN-01552899.

120. Nct. Make Safe Happen App Evaluation Study. https://clinicaltrialsgov/show/NCT02751203. 2016 2016. PMID: CN-01557689.

121. Nct. The Management of Diabetes in Everyday Life Program. https://clinicaltrialsgov/show/NCT02957513. 2016 2016. PMID: CN-01559842.

122. Nct. Digital Health Game as an Intervention Supporting Tobacco-related Health Literacy in Early Adolescents. https://clinicaltrialsgov/show/NCT02717910. 2016 2016. PMID: CN-01556807.

123. Nct. Evaluating How a Mobile App Can Improve Prenatal Care. https://clinicaltrialsgov/show/NCT03305003. 2017 2017. PMID: CN-01564707.

124. Nct. A Pilot Study of the Impact of the Patient Activated Learning System (PALS) on Knowledge Acquisition, Recall, and Decision Making. https://clinicaltrialsgov/show/NCT03156634. 2017 2017. PMID: CN-01494243.

125. Nct. Yo Puedo! Diabetes Self-Management Education + mHealth in Mexico City. https://clinicaltrialsgov/show/NCT03159299. 2017 2017. PMID: CN-01494306.

126. Nct. Interactive Technology for Media Literacy Drug Prevention in Community Groups. https://clinicaltrialsgov/show/NCT03157700. 2017 2017. PMID: CN-01494269.

127. Nct. The Morehouse-Emory Cardiovascular Center for Health Equity Study: clinical Intervention Project. https://clinicaltrialsgov/show/NCT03308812. 2017 2017. PMID: CN-01564809.

128. Nct. Development and Evaluation of an Electronic Health Record-based Medication Complete Communication (EMC2) Strategy. https://clinicaltrialsgov/show/NCT03652272. 2018 2018. PMID: CN-01662780.

129. Nct. Bridging the Gap - Tools for Finding Health, Mental Health and Wellness Resources for University and College Students. https://clinicaltrialsgov/show/NCT03412461. 2018 2018. PMID: CN-01522359.

130. Nct. Cognitive Behavioral Therapy for African Americans With Uncontrolled Type-2 Diabetes. https://clinicaltrialsgov/show/NCT03562767. 2018 2018. PMID: CN-01609308.

131. Nct. Evaluation Study of the Online High School Media Aware Program. https://clinicaltrialsgov/show/NCT04035694. 2019 2019. PMID: CN-01983454.

132. Nct. Atrial Fibrillation Health Literacy and Information Technology Trial in Rural PA Counties. https://clinicaltrialsgov/show/NCT04076020. 2019 2019. PMID: CN-01968325.

133. Nct. Atrial Fibrillation Health Literacy and Information Technology Trial in Pittsburgh, PA. https://clinicaltrialsgov/show/NCT04075994. 2019 2019. PMID: CN-01983949.

134. Nct. Face-it: health Promotion for Women With Prior Gestational Diabetes. https://clinicaltrialsgov/show/NCT03997773. 2019 2019. PMID: CN-01952810.

135. Nct. Be a Mom: effectiveness of an eHealth Intervention for Promoting Maternal Mental Health. https://clinicaltrialsgov/show/NCT04055974. 2019 2019. PMID: CN-01966436.

136. Nct. Preemie Prep For Parents (P3) Mobile App: home Antenatal Prematurity Education. https://clinicaltrialsgov/show/NCT04093492. 2019 2019. PMID: CN-01991941.

137. Nct. EHR-Embedded Decision Support to Prevent Stroke in Patients With AF. https://clinicaltrialsgov/show/NCT04099485. 2019 2019. PMID: CN-01992096.

138. Nct. Multi-Level Communication Strategies for HPV Vaccination in Hmong Adolescents. https://clinicaltrialsgov/show/NCT04017143. 2019 2019. PMID: CN-01953254.

139. Neuenschwander LM, Abbott A, Mobley AR. Comparison of a web-based vs in-person nutrition education program for low-income adults. J Acad Nutr Diet. 2013 Jan;113(1):120-6. PMID: 23092741. doi: 10.1016/j.jand.2012.07.034.

140. Newton KT, Ashley A. Pilot study of a web-based intervention for adolescents with type 1 diabetes. J Telemed Telecare. 2013 Dec;19(8):443-9. PMID: 24197399. doi: 10.1177/1357633X13512069.

141. Olmos-Ochoa TT, Niv N, Hellemann G, Cohen AN, Oberman R, Goldberg R, et al. Barriers to participation in web-based and in-person weight management interventions for serious mental illness. Psychiatr Rehabil J. 2019 Sep;42(3):220-8. PMID: 31081651. doi: 10.1037/prj0000363.

142. Ontario HQ. Ontario health technology assessment series: Internet-delivered cognitive behavioural therapy for major depression and anxiety disorders: A health technology assessment. Ontario Health Technology Assessment Series. 2019 2019;19(6):1-199.

143. Pachankis JE, Lelutiu-Weinberger C, Golub SA, Parsons JT. Developing an online health intervention for young gay and bisexual men. AIDS Behav. 2013 Nov;17(9):2986-98. PMID: 23673791. doi: 10.1007/s10461-013-0499-8.

144. Pakarinen A, Flemmich M, Parisod H, Selanne L, Hamari L, Aromaa M, et al. Protocol for digital intervention for effective health promotion of small children-A cluster randomized trial. J Adv Nurs. 2018 Jul;74(7):1685-99. PMID: 29517802. doi: 10.1111/jan.13561.

145. Patel MR, Heisler M, Piette JD, Resnicow K, Song PXK, Choe HM, et al. Study protocol: CareAvenue program to improve unmet social risk factors and diabetes outcomes- A randomized controlled trial. Contemp Clin Trials. 2020 Jan 7;89:105933. PMID: 31923472. doi: 10.1016/j.cct.2020.105933.

146. Patten CA, Croghan IT, Meis TM, Decker PA, Pingree S, Colligan RC, et al. Randomized clinical trial of an Internet-based versus brief office intervention for adolescent smoking cessation. Patient Educ Couns. 2006 Dec;64(1-3):249-58. PMID: 16616449. doi: 10.1016/j.pec.2006.03.001.

147. Perestelo-Perez L, Rivero-Santana A, Sanchez-Afonso JA, Perez-Ramos J, Castellano-Fuentes CL, Sepucha K, et al. Effectiveness of a decision aid for patients with depression: A randomized controlled trial. Health Expect. 2017 Oct;20(5):1096-105. PMID: 28295915. doi: 10.1111/hex.12553.

148. Perry TT, Halterman JS, Brown RH, Luo C, Randle SM, Hunter CR, et al. Results of an asthma education program delivered via telemedicine in rural schools. Ann Allergy Asthma Immunol. 2018 Apr;120(4):401-8. PMID: 29471032. doi: 10.1016/j.anai.2018.02.013.

149. Piette JD, Marinec N, Janda K, Morgan E, Schantz K, Yujra AC, et al. Structured Caregiver Feedback Enhances Engagement and Impact of Mobile Health Support: A Randomized Trial in a Lower-Middle-Income Country. Telemed J E Health. 2016 Apr;22(4):261-8. PMID: 26352854. doi: 10.1089/tmj.2015.0099.

150. Poelman MP, Steenhuis IHM, de Vet E, Seidell JC. The Development and Evaluation of an Internet-Based Intervention to Increase Awareness About Food Portion Sizes: A Randomized, Controlled Trial. Journal of Nutrition Education & Behavior. 2013 2013;45(6):701-7. PMID: 107920158. Language: English. Entry Date: 20140213. Revision Date: 20150712. Publication Type: Journal Article.

151. Politi MC, Barker AR, Kaphingst KA, McBride T, Shacham E, Kebodeaux CS. Show Me My Health Plans: a study protocol of a randomized trial testing a decision support tool for the federal health insurance marketplace in Missouri. BMC Health Serv Res. 2016 Feb 16;16:55. PMID: 26880251. doi: 10.1186/s12913-016-1314-9.

152. Politi MC, Lee CN, Philpott-Streiff SE, Foraker RE, Olsen MA, Merrill C, et al. A randomized controlled trial evaluating the BREASTChoice tool for personalized decision support about breast reconstruction after mastectomy. Annals of Surgery. 2020 2020;271(2):230-7.

153. Potdar R, Karki S, Dourado CM, Mohiuddin K, Djibo DA, Leighton JC, et al. A randomized, controlled trial to assess a multi-level intervention to improve adherence to oral cancer medications. Journal of Clinical Oncology. 2018 2018;36(15).

154. Prabhakaran D, Jha D, Ajay VS, Roy A, Perel P. Response by Prabhakaran et al to Letter Regarding Article, "effectiveness of an mHealth-Based Electronic Decision Support System for Integrated Management of Chronic Conditions in Primary Care: The mWellcare Cluster-Randomized Controlled Trial". Circulation. 2019 2019;139(24):E1039.

155. Przytula K, Bailey SC, Galanter WL, Lambert BL, Shrestha N, Dickens C, et al. A primary care, electronic health record-based strategy to promote safe drug use: study protocol for a randomized controlled trial. Trials. 2015 Jan 27;16:17. PMID: 25622970. doi: 10.1186/s13063-014-0524-x.

156. Reder M, Kolip P. Does a decision aid improve informed choice in mammography screening? Study protocol for a randomized controlled trial. BMC Womens Health. 2015 Jul 22;15:53. PMID: 26198675. doi: 10.1186/s12905-015-0210-5.

157. Ritamarie J. Effects of parent-focused media interventions on body mass index, waist size, self-perception, family eating habits, and family activity habits in overweight Hispanic children: Teachers College, Columbia University; 2009.

158. Roberto A, Colombo C, Candiani G, Giordano L, Mantellini P, Paci E, et al. Personalised informed choice on evidence and controversy on mammography screening: study protocol for a randomized controlled trial. BMC Cancer. 2017 Jun 19;17(1):429. PMID: 28629329. doi: 10.1186/s12885-017-3428-9.

159. Robinson JK, Friedewald JJ, Desai A, Gordon EJ. Response Across the Health-Literacy Spectrum of Kidney Transplant Recipients to a Sun-Protection Education Program Delivered on Tablet Computers: Randomized Controlled Trial. JMIR Cancer. 2015 Aug 18;1(2):e8. PMID: 28410176. doi: 10.2196/cancer.4787.

160. Rosenberger E, Abebe K, Belnap BH, Karp JF, Rollman BL. Predictors of engagement in an internet support group for treating mood and anxiety disorders in primary care. Journal of General Internal Medicine. 2014 2014;29:S177-S8.

161. Sakakibara BM, Chakrabarti S, Krahn A, Mackay MH, Sedlak T, Singer J, et al. Delivery of Peer Support Through a Self-Management mHealth Intervention (Healing Circles) in Patients With Cardiovascular Disease: Protocol for a Randomized Controlled Trial. JMIR Res Protoc. 2019 Jan 11;8(1):e12322. PMID: 30635261. doi: 10.2196/12322.

162. Sarfati D, McLeod M, Stanley J, Signal V, Stairmand J, Krebs J, et al. BetaMe: impact of a comprehensive digital health programme on HbA1c and weight at 12 months for people with diabetes and pre-diabetes: study protocol for a randomised controlled trial. Trials. 2018 Mar 5;19(1):161. PMID: 29506562. doi: 10.1186/s13063-018-2528-4.

163. Sayakhot P, Carolan-Olah M, Steele C. Use of a web-based educational intervention to improve knowledge of healthy diet and lifestyle in women with Gestational Diabetes Mellitus compared to standard clinic-based education. BMC Pregnancy Childbirth. 2016 Aug 5;16(1):208. PMID: 27495978. doi: 10.1186/s12884-016-0996-7.

164. Schomerus G, Angermeyer MC, Baumeister SE, Stolzenburg S, Link BG, Phelan JC. An online intervention using information on the mental health-mental illness continuum to reduce stigma. Eur Psychiatry. 2016 Feb;32:21-7. PMID: 26802980. doi: 10.1016/j.eurpsy.2015.11.006.

165. Schweiger S, Cress U. How Confidence in Prior Attitudes, Social Tag Popularity, and Source Credibility Shape Confirmation Bias Toward Antidepressants and Psychotherapy in a Representative German Sample: Randomized Controlled Web-Based Study. J Med Internet Res. 2019 Apr 23;21(4):e11081. PMID: 31012865. doi: 10.2196/11081.

166. Scull TM, Kupersmidt JB, Malik CV, Keefe EM. Examining the efficacy of an mHealth media literacy education program for sexual health promotion in older adolescents attending community college. J Am Coll Health. 2018 Apr;66(3):165-77. PMID: 29068772. doi: 10.1080/07448481.2017.1393822.

167. Scull TM, Kupersmidt JB, Weatherholt TN. The effectiveness of online, family-based media literacy education for substance abuse prevention in elementary school children: Study of the Media Detective Family program. J Community Psychol. 2017 Aug;45(6):796-809. PMID: 28989205. doi: 10.1002/jcop.21893.

168. Scull TM, Malik CV, Morrison A, Keefe EM. Study protocol for a randomized controlled trial to evaluate a web-based comprehensive sexual health and media literacy education program for high school students. Trials. 2020 Jan 8;21(1):50. PMID: 31915060. doi: 10.1186/s13063-019-3992-1.

169. Shi D. Study on management model of chinese hypertensive patients based on health literacy and mobile internet. Journal of Hypertension. 2018 2018;36:e183.

170. Skrovseth SO, Arsand E, Godtliebsen F, Joakimsen RM. Model-driven diabetes care: study protocol for a randomized controlled trial. Trials. 2013 May 14;14(1):139. PMID: 23672413. doi: 10.1186/1745-6215-14-139.

171. Steffen LE, Boucher KM, Damron BH, Pappas LM, Walters ST, Flores KG, et al. Efficacy of a Telehealth Intervention on Colonoscopy Uptake When Cost Is a Barrier: the Family CARE Cluster Randomized Controlled Trial. Cancer epidemiology, biomarkers & prevention. 2015 2015;24(9):1311‐8. PMID: CN-01096654.

172. Stockwell MS, Hofstetter AM, DuRivage N, Barrett A, Fernandez N, Vargas CY, et al. Text message reminders for second dose of influenza vaccine: a randomized controlled trial. Pediatrics. 2015 Jan;135(1):e83-91. PMID: 25548329. doi: 10.1542/peds.2014-2475.

173. T MS, C VM, E MK, Schoemann A. Evaluating the Short-term Impact of Media Aware Parent, a Web-based Program for Parents with the Goal of Adolescent Sexual Health Promotion. J Youth Adolesc. 2019 Sep;48(9):1686-706. PMID: 31304562. doi: 10.1007/s10964-019-01077-0.

174. Thabrew H, D'Silva S, Darragh M, Goldfinch M, Meads J, Goodyear-Smith F. Comparison of YouthCHAT, an Electronic Composite Psychosocial Screener, With a Clinician Interview Assessment for Young People: Randomized Controlled Trial. J Med Internet Res. 2019 Dec 3;21(12):e13911. PMID: 31793890. doi: 10.2196/13911.

175. Till B, Tran US, Voracek M, Niederkrotenthaler T. Beneficial and harmful effects of educative suicide prevention websites: randomised controlled trial exploring Papageno v. Werther effects. Br J Psychiatry. 2017 Aug;211(2):109-15. PMID: 28522433. doi: 10.1192/bjp.bp.115.177394.

176. Tongpeth J, Du H, Barry T, Clark RA. Effectiveness of an Avatar application for teaching heart attack recognition and response: A pragmatic randomized control trial. J Adv Nurs. 2020 Jan;76(1):297-311. PMID: 31566810. doi: 10.1111/jan.14210.

177. Topooco N, Andersson G. Treating adolescent depression: Development and results for a chat-and internet-based CBT intervention targeting youth in a community setting. Bipolar Disorders. 2019 2019;21:54.

178. Usher-Smith JA, Masson G, Mills K, Sharp SJ, Sutton S, Klein WMP, et al. A randomised controlled trial of the effect of providing online risk information and lifestyle advice for the most common preventable cancers: study protocol. BMC Public Health. 2018 Jun 26;18(1):796. PMID: 29940914. doi: 10.1186/s12889-018-5712-2.

179. van den Brink JL, Moorman PW, de Boer MF, Hop WC, Pruyn JF, Verwoerd CD, et al. Impact on quality of life of a telemedicine system supporting head and neck cancer patients: a controlled trial during the postoperative period at home. J Am Med Inform Assoc. 2007 Mar-Apr;14(2):198-205. PMID: 17213498. doi: 10.1197/jamia.M2199.

180. Vernon SW, Bartholomew LK, McQueen A, Bettencourt JL, Greisinger A, Coan SP, et al. A randomized controlled trial of a tailored interactive computer-delivered intervention to promote colorectal cancer screening: sometimes more is just the same. Annals of Behavioral Medicine. 2011 2011;41(3):284-99. PMID: 104904662. Language: English. Entry Date: 20110930. Revision Date: 20180307. Publication Type: journal article.

181. Vosgerau S, Urbanski D, Kollenbach P, Schone M, Gedamke M, Donatz V, et al. Easipro3-enhancing satisfaction with prostate cancer treatment decision with the mobile health program prostana: a multicenter randomized controlled trial. Value in health. 2017 2017;20(9):A504‐. PMID: CN-01431372.

182. Wallace P, Struzzo P, Della Vedova R, Scafuri F, Tersar C, Lygidakis C, et al. Randomised controlled non-inferiority trial of primary care-based facilitated access to an alcohol reduction website. BMJ Open. 2017 Nov 3;7(11):e014576. PMID: 29102982. doi: 10.1136/bmjopen-2016-014576.

183. Walther B, Hanewinkel R, Morgenstern M. Effects of a brief school-based media literacy intervention on digital media use in adolescents: cluster randomized controlled trial. Cyberpsychol Behav Soc Netw. 2014 Sep;17(9):616-23. PMID: 25126888. doi: 10.1089/cyber.2014.0173.

184. Westlake C, Evangelista LS, Stromberg A, Ter-Galstanyan A, Vazirani S, Dracup K. Evaluation of a Web-based education and counseling pilot program for older heart failure patients. Prog Cardiovasc Nurs. 2007 Winter;22(1):20-6. PMID: 17342002. doi: 10.1111/j.0889-7204.2007.05703.x.

185. White V, Farrelly A, Pitcher M, Hill D. Does access to an information‐based, breast cancer specific website help to reduce distress in young women with breast cancer? Results from a randomised trial. European Journal of Cancer Care. 2018 2018;27(6):N.PAG-N.PAG. PMID: 133260593. Language: English. Entry Date: 20181201. Revision Date: 20191101. Publication Type: Article. Journal Subset: Core Nursing.

186. Whittemore R, Grey M, Lindemann E, Ambrosino J, Jaser S. Development of an Internet coping skills training program for teenagers with type 1 diabetes. Comput Inform Nurs. 2010 Mar-Apr;28(2):103-11. PMID: 20182161. doi: 10.1097/NCN.0b013e3181cd8199.

187. Wiljer D, Abi-Jaoude A, Johnson A, Ferguson G, Sanches M, Levinson A, et al. Enhancing Self-Efficacy for Help-Seeking Among Transition-Aged Youth in Postsecondary Settings With Mental Health and/or Substance Use Concerns, Using Crowd-Sourced Online and Mobile Technologies: The Thought Spot Protocol. JMIR Res Protoc. 2016 Nov 4;5(4):e201. PMID: 27815232. doi: 10.2196/resprot.6446.

188. Wiljer D, Urowitz S, Barbera L, Chivers ML, Quartey NK, Ferguson SE, et al. A qualitative study of an internet-based support group for women with sexual distress due to gynecologic cancer. Journal of Cancer Education. 2011 2011;26(3):451-8. PMID: 104679040. Language: English. Entry Date: 20111028. Revision Date: 20171115. Publication Type: journal article.

189. Williams ED, Bird D, Forbes AW, Russell A, Ash S, Friedman R, et al. Randomised controlled trial of an automated, interactive telephone intervention (TLC Diabetes) to improve type 2 diabetes management: baseline findings and six-month outcomes. BMC Public Health. 2012 Aug 3;12:602. PMID: 22857017. doi: 10.1186/1471-2458-12-602.

190. Wolff JL, Aufill J, Echavarria D, Heughan JA, Lee KT, Connolly RM, et al. Sharing in care: engaging care partners in the care and communication of breast cancer patients. Breast Cancer Res Treat. 2019 Aug;177(1):127-36. PMID: 31165374. doi: 10.1007/s10549-019-05306-9.

191. Woodhead ZVJ, Kerry SJ, Aguilar OM, Ong YH, Hogan JS, Pappa K, et al. Randomized trial of iReadMore word reading training and brain stimulation in central alexia. Brain. 2018 Jul 1;141(7):2127-41. PMID: 29912350. doi: 10.1093/brain/awy138.

192. Yeates K, Campbell N, Maar MA, Perkins N, Liu P, Sleeth J, et al. The Effectiveness of Text Messaging for Detection and Management of Hypertension in Indigenous People in Canada: Protocol for a Randomized Controlled Trial. JMIR Res Protoc. 2017 Dec 19;6(12):e244. PMID: 29258978. doi: 10.2196/resprot.7139.

193. Zoellner J, You W, Almeida F, Blackman KC, Harden S, Glasgow RE, et al. The Influence of Health Literacy on Reach, Retention, and Success in a Worksite Weight Loss Program. Am J Health Promot. 2016 Mar;30(4):279-82. PMID: 27404064. doi: 10.1177/0890117116639558.

## Duplicates (n=4)

1. Petersen C, Bertram N, Schliffke M, Amelung VE, Göhl M, Binder S, et al. DIMINI - Activation of health literacy in persons with an increased risk of type 2 diabetes mellitus by coaching at general practitioners: Evaluation concept and study design. Diabetologie und Stoffwechsel. 2018 2018;13.

2. Shi D, Lyu X, Chen X. Study on economic evaluation of management model in chinese hypertensive patients based on health literacy and mobile internet. Journal of Hypertension. 2018 2018;36:e183.

3. Vosgerau S, Urbanski D, Kollenbach P, Schöne M, Gedamke M, Donatz V, et al. Easipro3-enhancing satisfaction with prostate cancer treatment decision with the mobile health program prostana: A multicenter randomized controlled trial. Value in Health. 2017 2017;20(9):A504.

4. Rezvani F, Härter M, Dirmaier J. Promoting a home-based walking exercise using telephone-based health coaching and activity monitoring for patients with intermittent claudication (TeGeCoach): Protocol for a randomized controlled trial. PPmP Psychotherapie Psychosomatik Medizinische Psychologie. 2018 2018;68(8):e43.

## PDF not available (n=9)

1. Carter JA, O'Shea M, Walton A, Thorndike AN, Donelan K, Metlay J. Implementing 30 day patient-community health worker pairings at the time of hospital discharge. Journal of General Internal Medicine. 2018 2018;33(2):796.

2. Inoue R, Nakayama M, Shibuya A, Ohkubo T, Asayama K, Metoki H, et al. Large scale intervention study to hypertension with information technology: Hypertension objective treatment based on Measurement by Electrical Devices of Blood Pressure (HOMED-BP) Study. AMIA Annu Symp Proc. 2008 Nov 6:986. PMID: 18998824.

3. Jessop AB, Bass SB, Gashat MT, Al Hajii M, Forry J. Creating and testing usability of an mhealth tool using targeted messages to affect HCV treatment decisions in hcv+ methadone patients. Hepatology. 2017 2017;66:316A.

4. Mehta MP, Sinha P, Kanwar K, Inman A, Albanese M, Fahl W. Evaluation of Internet-based oncologic teaching for medical students. J Cancer Educ. 1998 Winter;13(4):197-202. PMID: 9883777. doi: 10.1080/08858199809528546.

5. Roberto A, Colombo C, Candiani G, Giordano L, Mantellini P, Paci E, et al. [Decision aid and informed decision on breast cancer screening: the "DonnaInformata-Mammografia" project.]. Recenti Prog Med. 2018 Feb;109(2):139-42. PMID: 29493642. doi: 10.1701/2865.28911.

6. Sharma S, Mishuris RG, Gergen-Barnett K, Maypole J. Usability of M-health applications for safety net populations. Journal of General Internal Medicine. 2019 2019;34(2):S396.

7. Sheridan SL, Draeger LB, Pignone M, Keyserling TC. Patients' choices for lifestyle change versus medication use to reduce elevated CVD risk. Journal of General Internal Medicine. 2012 2012;27:S268.

8. Valenza JA, Spence JM, Taylor D, Walji MF. SmartConsent: a computerized informed consent for dental patients. AMIA Annu Symp Proc. 2008 Nov 6:1161. PMID: 18998872.

9. Witteman H, Chignell M, Krahn M. A recommender system for prostate cancer websites. AMIA Annu Symp Proc. 2008 Nov 6:1177. PMID: 18999034.
