# Supplementary material for: Application of the eHealth Literacy Model in Digital Health Interventions: Scoping Review
Source: J Med Internet Res. 2021 Jun 3;23(6):e23473. doi: 10.2196/23473 (PMC8212628; doi:10.2196/23473)
Supplement: Multimedia Appendix 3 [file jmir_v23i6e23473_app3.docx]

## Multimedia Appendix 3: Characteristics of included studies with references

Notes: The studies are reported alphabetically. References are in JMIR reference style.

[Multimedia Appendix 3: Characteristics of included studies with references 1](#_Toc53851607)

[1. Mobile-based Interventions (40 studies) 2](#_Toc53851608)

[2. Web-based Interventions (68 studies) 7](#_Toc53851609)

[3. Telehealth Interventions (10 studies) 15](#_Toc53851610)

[4. Electronic Health Records (5 studies) 16](#_Toc53851611)

[5. Hybrid interventions (8 studies) 17](#_Toc53851612)

[Reference List 18](#_Toc53851613)

### Mobile-based Interventions (40 studies)

| **Study ID [reference]**  **Trial number** | **Country** | **Technology used** | **Health condition category** | **Domains of the eHealth literacy model assessed** |
| --- | --- | --- | --- | --- |
| Bakker 2018 [1] | Australia | App | Mental health: Mental health in general | Health Literacy |
| Bukova 2018 [2]  NCT03329053 | Slovakia | App | NCDs/Chronic conditions: Cardiovascular diseases in general | Health Literacy |
| Chu 2019 [3] | New Zealand | Text messaging | Mental health: Parental competence and mental health literacy | Health Literacy |
| Connor 2017 [4]  ACTRN12617001246370 | Australia | App | Other health topics: Ulcerative colitis | Health Literacy |
| Cruvinel 2019 [5]  U1111-1216-1393; PRR1-10.2196/13656 | Brazil | Text messaging | Other health topics: Early childhood caries | Computer/digital |
| Cyan 2016 [6] | Pakistan | IVR/Voicemail | Maternal and infant health: General health | Health Literacy |
| Delbaere 2019 [7]  ACTRN12619000540112 | Australia | App | Ageing: Accidental falls | Health Literacy |
| Ekstedt 2018 [8,9]  ISRCTN18055968 | Sweden | App | NCDs/Chronic conditions: Prostate cancer | Health Literacy |
| Forren 2015 [10]  NCT02610894 | USA | App | Health education topics: Orthopedic Surgery | Health Literacy |
| Hwayoung 2017 [11] | USA | App | Sexual and reproductive health: HIV prevention | Health Literacy |
| Jensen 2019 [12]  NCT04058847 | Denmark | App | NCDs/Chronic conditions: Risk Communication; Cardiovascular Risk Factor | Health Literacy |
| Jessop 2020 [13] | USA | App | NCDs/Chronic conditions: Hepatitis C | Health Literacy |
| Kamal 2016 [14,15]  NCT02354040 | USA | IVR/Voicemail | NCDs/Chronic conditions: Vascular Disease; Cerebrovascular Disorders; Ischemic Heart Disease; Coronary Artery Disease | Health Literacy |
| Khader 2019 [16] | Jordan | App | Other health topics: Vaccination | Computer/digital |
| Khaleghi 2019 [17] | Iran | Whatsapp, Telegram | Health education topics: Health literacy evaluation | Health Literacy |
| Khalesi 2019 [18]  IRCT20180707040364N1 | Iran | App | Maternal and infant health: Pregnancy | Health Literacy |
| Langius-Eklöf 2017 [19]  NCT02477137; NCT02479607 | Sweden | App | NCDs/Chronic conditions: Breast cancer, prostate cancer | Health Literacy |
| Lanpher 2016 [20] | USA | IVR/Voicemail | NCDs/Chronic conditions: Obesity; weight gain prevention | Health Literacy |
| Lepley 2019 [21] | USA | App | Health education topics: Acute illness education | Health Literacy |
| Livingston 2016 [22]  ACTRN12616001251415 | Australia | App | NCDs/Chronic conditions; Mental health: Any cancer, Anxiety | Health Literacy |
| Lotto 2018 [23]  U1111-1216-1393 | Brazil | App | Other health topics: Early childhood caries | Computer/digital |
| Lunde 2019 [24]  NCT03174106 | Norway | App | NCDs/Chronic conditions: Cardiac Rehabilitation | Health Literacy |
| Lygidakis 2019 [25]  NCT03376607 | Luxemburg | App | NCDs/Chronic conditions: Diabetes Mellitus | Health Literacy |
| Miller 2018 [26] | USA | App | NCDs/Chronic conditions: Colorectal cancer | Health Literacy |
| Milner 2019 [27]  ACTRN12619000625178 | Australia | App | Mental health: Suicide health literacy | Health Literacy |
| Olives 2012 [28,29] | USA | Text messaging; IVR/Voicemail | NCDs/Chronic conditions: Outpatient antibiotics | Health Literacy |
| Ownby 2017 [30,31]  NCT02922439 | USA | App | NCDs/Chronic conditions; Mental health: Fatigue Depression, Pain Sleep Wake Disorders Chronic Disease | Health Literacy |
| Parisod 2018 [32] | Finland | Game | Substance use: Smoking, health education | Health Literacy |
| Patel 2019 [33,34] | USA | App | NCDs/Chronic conditions: Obesity | Health Literacy |
| Patzer 2016 [35] | USA | App | Other health topics: Kidney transplantation | Health Literacy |
| Patzer 2018 [36]  NCT02235571 | USA | App | NCDs/Chronic conditions: End-Stage Kidney Disease | Health Literacy |
| Petersen 2018 [37] | Germany | App | NCDs/Chronic conditions: Type 2 diabetes mellitus | Health Literacy |
| Rajabi 2018 [38]  IRCT20180515039675N1 | Iran | Text messaging | NCDs/Chronic conditions: Breast cancer | Health Literacy |
| Redfern 2019 [39] | Australia | App | NCDs/Chronic conditions: Cardiovascular risk management | Computer/digital |
| Sanchez 2017 [40] | USA | Game | Mental health: General mental health | Basic |
| Skau 2016 [41]  NCT02617693 | South Africa | App | NCDs/Chronic conditions: Diabetes Mellitus, Type 2 | Health Literacy |
| van de Hei 2019 [42]  NL7854 | Netherlands | App | NCDs/Chronic conditions: Asthma | Computer/digital |
| Wong 2019 [43]  NCT04054258 | Singapore | App | NCDs/Chronic conditions: Coronary Heart Disease | Health Literacy |
| Wood 2011 [44] | United Kingdom | Text messaging | Other health topics: Phonological awareness | Basic |
| Yee 2014 [45] | USA | App | Health education topics: Patient knowledge, genetic screening | Health Literacy |

### Web-based Interventions (68 studies)

| **Study ID [reference]**  **Trial number** | **Country** | **Technology used** | **Health condition category** | **Domains of the eHealth literacy model assessed** |
| --- | --- | --- | --- | --- |
| Arao 2015 [46]  JPRN-UMIN000019376 | Japan | Internet-based program | Health education topics: Adult health | Health Literacy |
| Arciuli 2019 [47] | Australia | Web application | Mental health | Basic literacy |
| Austvoll-Dahlgren 2012 [48]  NCT01266798 | Norway | Web Portal | Health education topics: Health literacy skills | Health Literacy |
| Batterham 2014 [49] | Australia | Online psychological education | Mental health: Depression, Anxiety Suicide prevention | Health Literacy |
| Bauer 2018 [50,51]  DRKS00014679 | Germany | Internet-Based program | Mental health: Eating disorders | Health Literacy |
| Blalock 2017 [52] | USA | Online Patient Community | NCDs/Chronic conditions: Rheumatoid arthritis | Health Literacy |
| Brettle 2013 [53] | United Kingdom | Web-based intervention | Health education topics: Information literacy | Information |
| Briges 2015 [54] | Hong Kong | Social media advertisements | Health education topics: Health Literacy Education | Health Literacy |
| Camerini 2012 [55] | Switzerland | Web-Based Intervention | NCDs/Chronic conditions: Fibromyalgia syndrome | Health Literacy |
| Carroll 2019 [56] | USA | Web-searching for health information | Sexual and reproductive health: HIV prevention | Computer/digital |
| Christensen 2008 [57,58]  ISRCTN69603913 | Australia | Internet-assisted therapy | Mental health: Depressive and anxiety disorders | Health Literacy |
| Chu AY 2008 [59] | USA | Online health information seeking | Ageing: Older adults health | Computer/digital |
| Comer 2018 [60]  NCT03707158 | USA | Online digital intervention | Mental health: Child or adolescent Anxiety | Computer/digital |
| Dalgıç 2019 [61]  NCT04144478 | Turkey | Web-based | Other health topics: Epilepsy | Computer/digital |
| Delbaere 2018 [62]  ACTRN12618000926235 | Australia | Online cognitive behavioral therapy program | Ageing: Concerns about falling, falls | Health Literacy |
| Demir 2018 [63]  NCT03708835 | Turkey | Web-based intervention | NCDs/Chronic conditions: Stroke | Computer/digital |
| Farrand 2008 [64]  ISRCTN65803720 | United Kingdom | Web-based intervention | Mental health: Depression, with or without anxiety | Health Literacy |
| Friedl 2006 [65] | Germany | e-learning | Health education topics: Students’ procedural knowledge in operative procedures in heart surgery | Computer/digital |
| Fu 2015 [66] | Hong Kong | Website: Social media (Facebook); email | Mental health: Depression, help-seeking | Health Literacy |
| Gabel 2017 [67]  NCT03253822 | Denmark | Web-based | NCDs/Chronic conditions: Decision-making knowledge, attitudes, practice | Health Literacy |
| Geller 2013 [68,69] | USA | Patient-centered website | Other health topics: Palliative care and end-of-life decision-making | Health Literacy |
| Girardin 2016 [70]  NCT02507089 | USA | Web-based | Other health topics: Healthcare disparities, minority health, sleep disorders | Health Literacy |
| Goff 2013 [71]  NCT01784575 | USA | Website | Maternal and infant health: Pregnancy | Health Literacy |
| Griffiths 2009 [72]  ACTRN12609000497202 | Australia | Online intervention program | Mental health: Depression | Health Literacy |
| Griffiths 2013 [73]  ACTRN12613001083785 | Australia | Internet-based | Mental health: Depression, anxiety | Health Literacy |
| Guvenc 2016 [74]  NCT03009617 | Turkey | Web-Based | Health education topics: Adolescent health | Computer/digital |
| Harris 2018 [75]  ACTRN12617001508369 | Australia | eLearning courseware | NCDs/Chronic conditions: Obesity, low health literacy, chronic disease | Health Literacy + Computer/digital |
| Härter 2013 [76–78]  DRKS00003322 | Germany | Online intervention | NCDs/Chronic conditions: Type 2 diabetes | Health Literacy |
| Heckman 2015 [79] | USA | Internet intervention | NCDs/Chronic conditions: Skin cancer risk | Health Literacy |
| Hodges 2019 [80,81]  ACTRN12617001292369 | Australia | Web-based program | NCDs/Chronic conditions: Low back pain | Health Literacy |
| Hua 2018 [82]  NCT03534336 | Singapore | Online program | NCDs/Chronic conditions: Weight Reduction, health knowledge, attitudes, practice | Health Literacy |
| Jaffery 2008 [83]  NCT00693277 | USA | Internet-based | NCDs/Chronic conditions: Chronic Kidney Disease | Health Literacy |
| Jorm 2010 [84] | Australia | e-learning | Mental health: Mental health | Health Literacy |
| Kauer 2014 [85,86]  ACTRN12614000386639 | Australia | Web-based program | Mental health: Depression, anxiety, addiction | Health Literacy |
| Kimmerle 2016 [87] | Germany | Social media (forum) | Other health topics: self-efficacy; tentativeness | Scientific |
| Kiropoulos 2011 [88]  ISRCTN76460837 | Australia | Website Intervention | Mental health: Mental and behavioral disorders | Health Literacy |
| Kurz 2016 [89]  DRKS00009891 | Germany, France, United Kingdom | Internet-based | Mental health: Young onset dementia | Computer/digital |
| Lyles 2019 [90] | USA | Online video curriculum | NCDs/Chronic conditions: Chronic Disease | Health Literacy + Computer/digital |
| Martinez 2007 [91]  ISRCTN71327173 | United Kingdom | Online resources | Mental health: Mental and behavioral disorders | Health Literacy |
| McCaffery 2018 [92,93]  ACTRN12618001409268 | Australia | Online | NCDs/Chronic conditions: Diabetes, overweight/obesity, unhealthy snacking | Health Literacy |
| McCaffery 2019 [94]  ACTRN12617001194358 | Australia | Online intervention | Other health topics: Low health literacy, unhealthy snacking | Health Literacy |
| McClay 2009 [95,96]  ISRCTN41034162 | United Kingdom | Online life skills package | Mental health: Bulimia nervosa | Health Literacy |
| McLean 2018 [97]  ACTRN12618001408279 | Australia | Social media | Mental health: Body dissatisfaction, disordered eating, depressed mood | Media |
| Mitsuhashi 2018 [98] | Japan | Online intervention | Health education topics: eHealth literacy | Health Literacy + Computer/digital |
| Moll 2018 [99] | Canada | Online sessions | Mental health: Mental health literacy, workplace | Health Literacy |
| Muller 2013 [100,101]  ISRCTN43587048 | United Kingdom | Web-based | NCDs/Chronic conditions: Diabetes | Health Literacy |
| Nelson 2019 [102]  NCT04109443 | USA | Online sexual health media literacy materials | Sexual and reproductive health: Sexual Behavior | Media |
| Nilsson 2016 [103]  NCT02492191 | Sweden | Web-version of Quality of Recovery questionnaire | Other health topics: Postoperative complications | Health Literacy |
| Nyberg 2017 [104] | Sweden | Internet based tool | NCDs/Chronic conditions: Chronic obstructive pulmonary disease (COPD) | Health Literacy |
| Oddone 2019 [105–108]  NCT01828567 | USA | Web-portal | NCDs/Chronic conditions: Health Risk Appraisal, heart disease, prevention | Health Literacy |
| Oenema 2001 [109] | Netherlands | Web-based education | Other health topics: Nutrition education | Computer/digital |
| Pekow 2014 [110] | USA | Patient navigator website | Maternal and infant health: Pregnant women | Health Literacy |
| Proudfoot 2008 [111]  ACTRN12608000411347 | Australia | Web-based | Mental health: Mental health, bipolar disorder | Health Literacy |
| Reisi 2017 [112]  IRCT2017080535509N1 | Iran | Web-based | NCDs/Chronic conditions: Obesity or overweight | Health Literacy |
| Reupert 2019 [113]  ACTRN12619000335190 | Australia | Online intervention program | Mental health: Family related mental illness, depression, schizophrenia, anxiety | Health Literacy |
| Rosen 2015 [114]  NCT02601794 | USA | Web-based program | NCDs/Chronic conditions: Breast cancer | Health Literacy + Computer/digital |
| Schwinn 2019 [115] | USA | Web-based program | Substance use: Drug abuse | Media |
| Shensa 2016 [116] | USA | Web-based program | Substance use: Smoking | Media |
| Sidik 2015 [117,118]  ISRCTN39656144 | Malaysia | Web-based program | Mental health: Depression and anxiety | Health Literacy |
| Smith 2016 [119–121]  ACTRN12616000135415 | Australia | Website | Health education topics: Health literacy | Health Literacy |
| Stellamanns 2018 [122] | Germany | Experimental website | NCDs/Chronic conditions: Breast cancer, mammography | Basic |
| Sullivan 2016 [123] | USA | Prescription drug website | Mental health: Depression | Health Literacy |
| Swallow 2012 [124] | United Kingdom | Online | NCDs/Chronic conditions: Chronic kidney disease | Health Literacy |
| Van den Broucke 2014 [125] | Belgium | Web-Based | NCDs/Chronic conditions: Type 2 diabetes | Health Literacy |
| Weiner 2018 [126]  NCT03522740 | USA | Web-based | NCDs/Chronic conditions: Chronic Kidney Diseases, Kidney Failure | Health Literacy |
| Weinert 2005 [127] | USA | Website | NCDs/Chronic conditions: Chronic illness in general | Computer/digital |
| Williams 2008 [128]  ISRCTN93089066 | United Kingdom | Online CCBT package | NCDs/Chronic conditions: Diabetes  Mental health: Depression | Health Literacy |
| Xie 2011 [129] | USA | Website | Health education topics: eHealth Literacy | Computer/digital |

### Telehealth Interventions (10 studies)

| **Study ID [reference]**  **Trial number** | **Country** | **Technology used** | **Health condition category** | **Domains of the eHealth literacy model assessed** |
| --- | --- | --- | --- | --- |
| Bohingamu 2019 [130] | Australia | Telehealth intervention-technology platform | NCDs/Chronic conditions: Chronic disease management | Health Literacy |
| Boyne 2014 [131] | Netherlands | Tele-home care | NCDs/Chronic conditions: heart failure patients | Health Literacy |
| Dinesen 2019 [132]  NCT03388918 | Denmark | Telerehabilitation | NCDs/Chronic conditions: Heart Failure | Health Literacy; Computer/digital |
| Fields 2019 [133] | USA | Tablet + broadband access | E-Learning/multimedia course/web-based program/educational intervention | Ageing: Socially isolated older adults |
| Korsbakke 2016 [136] | Denmark | Tele-home care | NCDs/Chronic conditions: Chronic disease management | Health Literacy |
| Morony 2018 [137] | Australia | Telehealth service | Health education topics: Communication quality | Health Literacy |
| Rezvani 2018 [138] | Germany | Tele home care | Other health topics: Intermittent claudication | Health Literacy |
| Salisbury 2016 [139] | United Kingdom | Telemedicine/telehealth (device + internet) | Mental health: Depression | Computer/digital |
| Stockdill 2019 [140] | USA | Telehealth intervention | NCDs/Chronic conditions: Heart Failure | Health Literacy |
| Whelan 2017 [141]  ISRCTN17545949 | United Kingdom | Mobile apps and Fitbit devices | App/Computer Software/Decision support system | NCDs/Chronic conditions: Prediabetes |

### Electronic Health Records (5 studies)

| **Study ID [reference]**  **Trial number** | **Country** | **Technology category** | **Health condition category** | **Domains of the eHealth literacy model assessed** |
| --- | --- | --- | --- | --- |
| Ancker 2017 [142] | USA | Electronic Health Record | Health education topics: Patient instructions | Health Literacy |
| Apter 2015 [143] | USA | Electronic Health Record | NCDs/Chronic conditions: Asthma | Health Literacy + Computer/digital |
| Jackson 2018 [144]  NCT03434886 | Ireland | Electronic Health Record | NCDs/Chronic conditions: Cystic fibrosis | Health Literacy |
| Pavlik 2014 [145] | USA | Electronic Health Record | Health education topics: Patient satisfaction | Health Literacy |
| Persell 2016 [146,147]  NCT01578577 | USA | Electronic Health Record–Based Medication Support; App | NCDs/Chronic conditions: Hypertension | Health Literacy |

### Hybrid interventions (8 studies)

| **Study ID [reference]**  **Trial number** | **Country** | **Technology used** | **Health condition category** | **Domains of the eHealth literacy model assessed** |
| --- | --- | --- | --- | --- |
| Bostom 2011 [148] | Canada | EHR; App | NCDs/Chronic conditions: Bladder cancer | Computer/digital |
| Duncan 2014 [149,150]  ACTRN12611000081910 | Australia | Website; Text messaging | NCDs/Chronic conditions: Cardiovascular disease | Health Literacy |
| Hjollund 2017 [134,135] | Denmark | Telemedicine service; telePRO app | Other health topics: Epilepsy | Health Literacy |
| Johnson 2015 [151]  NCT01082978 | Australia | EHR; App | NCDs/Chronic conditions: Chronic Medical Conditions | Health Literacy |
| Navaneethan 2017 [152] | USA | EHR; App | NCDs/Chronic conditions: Chronic kidney disease (CKD) | Health Literacy |
| Ramaswamy 2019 [153]  NCT03984695 | USA | Website; Text messaging | NCDs/Chronic conditions: Cervical Cancer, breast Cancer  Sexual and reproductive health: Contraceptive Usage, Sexually Transmitted Diseases | Health Literacy |
| Redfern 2014 [154–156]  ACTRN12613000715774 | Australia | EHR; Website; App | NCDs/Chronic conditions: Cardiovascular disease | Health Literacy + Computer/digital |
| Wolf 2017 [157,158]  NCT03185741 | USA | Text messaging; EHR | NCDs/Chronic conditions: Diabetes Mellitus, Type 2 Medication Adherence | Health Literacy |

### Reference List

1. Bakker D, Kazantzis N, Rickwood D, Rickard N. A randomized controlled trial of three smartphone apps for enhancing public mental health. Behav Res Ther 2018 Oct;109:75–83. PMID:30125790

2. Zelko A, Bukova A, Kolarcik P, Bakalar P, Majercak I, Potocnikova J, Reijneveld SA, van Dijk JP. A randomized controlled trial to evaluate utilization of physical activity recommendations among patients of cardiovascular healthcare centres in Eastern Slovakia: study design and rationale of the AWATAR study. BMC Public Health 2018 Apr 4;18(1):454. PMID:29618329

3. Chu JTW, Wadham A, Jiang Y, Whittaker R, Stasiak K, Shepherd M, Bullen C. Effect of MyTeen SMS-Based Mobile Intervention for Parents of Adolescents: A Randomized Clinical Trial. JAMA Netw Open 2019 Sep 4;2(9):e1911120. PMID:31509210

4. Actrn. Use of an Internet-based Decision Aid (myAID) for Ulcerative Colitis Patients to Improve Quality of Life, Empowerment, Decision Making and Disease Control. http://www.who.int/trialsearch/Trial2.aspx?TrialID=ACTRN12617001246370 [Internet] 2017; Available from: https://www.cochranelibrary.com/central/doi/10.1002/central/CN-01885778/full

5. Aguirre PEA, Lotto M, Strieder AP, Cruvinel AFP, Cruvinel T. The Effectiveness of Educational Mobile Messages for Assisting in the Prevention of Early Childhood Caries: Protocol for a Randomized Controlled Trial. JMIR Res Protoc 2019 Sep 3;8(9):e13656. PMID:31482856

6. Cyan R, Hussain H, Shehki I, Ishaq Z, Khan T, Rothenberg R. Affordable technology for saving maternal and infant lives: moving on with solutions. Annals of global health 2016;82(3):382‐. [doi: 10.1016/j.aogh.2016.04.620]

7. Actrn. StandingTall-Plus: a 1-year randomised controlled trial of a novel multifactorial intervention for preventing falls in older people. http://www.who.int/trialsearch/Trial2.aspx?TrialID=ACTRN12619000540112 [Internet] 2019; Available from: https://www.cochranelibrary.com/central/doi/10.1002/central/CN-01970513/full

8. Ekstedt M, Schildmeijer K, Wennerberg C, Nilsson L, Wannheden C, Hellstrom A. Enhanced Patient Activation in Cancer Care Transitions: Protocol for a Randomized Controlled Trial of a Tailored Electronic Health Intervention for Men With Prostate Cancer. JMIR Res Protoc 2019 Mar 22;8(3):e11625. PMID:30900999

9. Isrctn. Supporting self-management by an e-health application in men with prostate cancer who have had their prostate removed. http://www.who.int/trialsearch/Trial2.aspx?TrialID=ISRCTN18055968 [Internet] 2018; Available from: https://www.cochranelibrary.com/central/doi/10.1002/central/CN-01904954/full

10. Nct. An mHealth Self-Management Program to Decrease Postoperative Symptom Distress. https://clinicaltrials.gov/show/NCT02610894 [Internet] 2015; Available from: https://www.cochranelibrary.com/central/doi/10.1002/central/CN-01553876/full

11. Hwayoung C. Development and Usability Evaluation of an mHealth Application for Symptom Self-Management in Underserved Persons Living with HIV [Internet]. 2017. Available from: http://search.ebscohost.com/login.aspx?direct=true&db=ccm&AN=130413209&site=ehost-live

12. Nct. Danish Evaluation of Your Heart Forecast. https://clinicaltrials.gov/show/NCT04058847 [Internet] 2019; Available from: https://www.cochranelibrary.com/central/doi/10.1002/central/CN-01983498/full

13. Jessop AB, Bass SB, Brajuha J, Alhajji M, Burke M, Gashat MT, Wellington C, Ventriglia N, Coleman J, D’Avanzo P. “Take Charge, Get Cured”: Pilot testing a targeted mHealth treatment decision support tool for methadone patients with hepatitis C virus for acceptability and promise of efficacy. Journal of Substance Abuse Treatment 2020;109:23–33. PMID:31856947

14. Kamal AK, Khalid W, Muqeet A, Jamil A, Farhat K, Gillani SRA, Zulfiqar M, Saif M, Muhammad AA, Zaidi F, Mustafa M, Gowani A, Sharif S, Bokhari SS, Tai J, Rahman N, Sultan FAT, Sayani S, Virani SS. Making prescriptions “talk” to stroke and heart attack survivors to improve adherence: Results of a randomized clinical trial (The Talking Rx Study). PLoS One 2018;13(12):e0197671. PMID:30571697

15. Kamal AK, Khalid W, Muqeet A, Jamil A, Gillani S, Farhat K, Muhammad AA, Zaidi F, Gowani A, Sharif S, Bokhari SS, Rahman N, Sultan FAT, Sayani S, Virani SS, Khoja A. Using a tailored health information technology driven intervention to improve health literacy and medication adherence in a pakistani population with vascular disease. Cerebrovascular Diseases 2016;42:29. PMID:26944938

16. Khader YS, Laflamme L, Schmid D, El-Halabi S, Abu Khdair M, Sengoelge M, Atkins S, Tahtamouni M, Derrough T, El-Khatib Z. Children Immunization App (CImA) Among Syrian Refugees in Zaatari Camp, Jordan: Protocol for a Cluster Randomized Controlled Pilot Trial Intervention Study. JMIR Res Protoc 2019 Oct 7;8(10):e13557. PMID:31593549

17. Khaleghi M, Shokravi FA, Peyman N, Moridi M. Evaluating the Effect of Educational Interventions on Health Literacy through Social Networking Services to Promote Students’ Quality of Life. Korean J Fam Med 2019 May;40(3):188–193. PMID:30997783

18. Irct20180707040364N. Survey the effect of educational intervention using mobile application on health literacy in pregnant women. http://www.who.int/trialsearch/Trial2.aspx?TrialID=IRCT20180707040364N1 [Internet] 2019; Available from: https://www.cochranelibrary.com/central/doi/10.1002/central/CN-01971254/full

19. Langius-Eklof A, Crafoord MT, Christiansen M, Fjell M, Sundberg K. Effects of an interactive mHealth innovation for early detection of patient-reported symptom distress with focus on participatory care: protocol for a study based on prospective, randomised, controlled trials in patients with prostate and breast cancer. BMC Cancer 2017 Jul 4;17(1):466. PMID:28676102

20. Lanpher MG, Askew S, Bennett GG. Health Literacy and Weight Change in a Digital Health Intervention for Women: A Randomized Controlled Trial in Primary Care Practice. J Health Commun 2016;21 Suppl 1:34–42. PMID:27043756

21. Lepley BE, Brousseau DC, May MF, Morrison AK. Randomized Controlled Trial of Acute Illness Educational Intervention in the Pediatric Emergency Department: Written Versus Application-Based Education. Pediatr Emerg Care [Internet] 2019 Jan 7; PMID:30624425

22. Actrn. A smartphone application for people with cancer to help reduce distress and unmet needs. http://www.who.int/trialsearch/Trial2.aspx?TrialID=ACTRN12616001251415 [Internet] 2016; Available from: https://www.cochranelibrary.com/central/doi/10.1002/central/CN-01872343/full

23. b6r7q RBR. Aleatory clinical trial: evaluation of the effect of an application for mobile phones as an aid in the prevention of Bottle Caries. http://www.who.int/trialsearch/Trial2.aspx?TrialID=RBR-2b6r7q [Internet] 2018; Available from: https://www.cochranelibrary.com/central/doi/10.1002/central/CN-01908618/full

24. Lunde P, Bye A, Bergland A, Nilsson BB. Effects of individualized follow-up with a smartphone-application after cardiac rehabilitation: protocol of a randomized controlled trial. BMC Sports Sci Med Rehabil 2019;11(1):34. PMID:31768261

25. Lygidakis C, Uwizihiwe JP, Kallestrup P, Bia M, Condo J, Vogele C. Community-and mHealth-based integrated management of diabetes in primary healthcare in Rwanda (D2;Rwanda): the protocol of a mixed-methods study including a cluster randomised controlled trial. BMJ open 2019;9(7). PMID:31345971

26. Miller DP Jr, Denizard-Thompson N, Weaver KE, Case LD, Troyer JL, Spangler JG, Lawler D, Pignone MP. Effect of a Digital Health Intervention on Receipt of Colorectal Cancer Screening in Vulnerable Patients: A Randomized Controlled Trial. Ann Intern Med 2018 Apr 17;168(8):550–557. PMID:29532054

27. Actrn. A blended face-to-face and smartphone intervention for suicide prevention in the construction industry (MATESMobile): protocol for a randomized controlled trial with MATES in Construction. http://www.who.int/trialsearch/Trial2.aspx?TrialID=ACTRN12619000625178 [Internet] 2019; Available from: https://www.cochranelibrary.com/central/doi/10.1002/central/CN-01972471/full

28. Olives TD, Patel RG, Nelson RS, Yew A, Joing S, Miner JR. Randomized controlled trial of three instructional modalities for patients prescribed outpatient antibiotics from the ED: the potential of cell phone technology to reach limited health literacy patients. Academic emergency medicine 2012;19:S219‐.

29. Olives TD, Patel RG, Nelson RS, Yew A, Joing S, Miner JR. Variation of patient preferences for written and cell phone instructional modality of discharge instructions by patient health literacy level. Academic emergency medicine 2012;19:S90.

30. Ownby RL, Acevedo A, Waldrop-Valverde D, Caballero J, Simonson M, Davenport R, Kondwani K, Jacobs RJ. A Mobile App for Chronic Disease Self-Management: Protocol for a Randomized Controlled Trial. JMIR Res Protoc 2017 Apr 5;6(4):e53. PMID:28381395

31. Nct. Health Literacy Assessment and Intervention to Reduce Disparities: FLIGHT/VIDAS II. https://clinicaltrials.gov/show/NCT02922439 [Internet] 2016; Available from: https://www.cochranelibrary.com/central/doi/10.1002/central/CN-01521276/full

32. Parisod H, Pakarinen A, Axelin A, Loyttyniemi E, Smed J, Salantera S. Feasibility of mobile health game “Fume” in supporting tobacco-related health literacy among early adolescents: a three-armed cluster randomized design. International journal of medical informatics 2018;113:26‐37. PMID:29602430

33. Patel ML, Hopkins CM, Brooks TL, Bennett GG. Comparing Self-Monitoring Strategies for Weight Loss in a Smartphone App: Randomized Controlled Trial. JMIR mHealth and uHealth 2019 Feb 28;7(2):e12209. [doi: 10.2196/12209]

34. Patel ML, Brooks TL, Bennett GG. Consistent self-monitoring in a commercial app-based intervention for weight loss: results from a randomized trial. J Behav Med 2019 Aug 8;8:08. PMID:31396820

35. Patzer RE, Basu M, Mohan S, Smith KD, Wolf M, Ladner D, Friedewald JJ, Chiles M, Russell A, McPherson L, Gander J, Pastan S. A Randomized Controlled Trial of a Mobile Clinical Decision Aid to Improve Access to Kidney Transplantation: iChoose Kidney. Kidney Int Rep 2016 May;1(1):34–42. PMID:27610423

36. Patzer RE, McPherson L, Basu M, Mohan S, Wolf M, Chiles M, Russell A, er JC, Friedewald JJ, Ladner D, Larsen CP, Pearson T, Pastan S. Effect of the iChoose Kidney decision aid in improving knowledge about treatment options among transplant candidates: A randomized controlled trial. American Journal of Transplantation 2018;18(8):1954–1965. PMID:29446209

37. Petersen C, Bertram N, Schliffke M, Amelung VE, Gohl M, Binder S, Bergen S. DIMINI - Activation of health literacy in persons with an increased risk of type 2 diabetes mellitus by coaching at general practitioners: evaluation concept and study design. Diabetologie und stoffwechsel [Internet] 2018;13. Available from: https://www.cochranelibrary.com/central/doi/10.1002/central/CN-01771046/full

38. Irct20180515039675N. Comparison the effect of education using compact disk versus a Messenger Service on health literacy and using screening methods of breast cancer in middle aged Iranian women. http://www.who.int/trialsearch/Trial2.aspx?TrialID=IRCT20180515039675N1 [Internet] 2018; Available from: https://www.cochranelibrary.com/central/doi/10.1002/central/CN-01905504/full

39. Redfern J, Usherwood T, Coorey G, Mulley J, Scaria A, Neubeck L, Hafiz N, Chow C, Peiris D. A consumer-direct digital health intervention for cardiovascular risk management in primary care: The Consumer Navigation of Electronic Cardiovascular Tools (CONNECT) randomised controlled trial. European Heart Journal 2019;40:3206. [doi: 10.1093/eurheartj/ehz746.0278]

40. Sanchez R, Brown E, Kocher K, DeRosier M. Improving Children’s Mental Health with a Digital Social Skills Development Game: A Randomized Controlled Efficacy Trial of Adventures aboard the S.S. GRIN. Games Health J 2017 Feb;6(1):19–27. PMID:28051877

41. Skau JK, Nordin AB, Cheah JC, Ali R, Zainal R, Aris T, Ali ZM, Matzen P, Biesma R, Aagaard-Hansen J, Hanson MA, Norris SA. A complex behavioural change intervention to reduce the risk of diabetes and prediabetes in the pre-conception period in Malaysia: study protocol for a randomised controlled trial. Trials 2016 Apr 27;17(1):215. PMID:27117703

42. Nl. A cluster Randomised Controlled Trial of the effectiveness, usability and acceptability of a smart inhaler programme in asthma patients: the ACCEPTANCE study. http://www.who.int/trialsearch/Trial2.aspx?TrialID=NL7854 [Internet] 2019; Available from: https://www.cochranelibrary.com/central/doi/10.1002/central/CN-01971129/full

43. Nct. Program Using a Mobile Application Versus Telephone Advice on Patients at Risk of Coronary Heart Disease : a Pilot RCT. https://clinicaltrials.gov/show/NCT04054258 [Internet] 2019; Available from: https://www.cochranelibrary.com/central/doi/10.1002/central/CN-01966381/full

44. Wood C, Jackson E, Hart L, Plester B, Wilde L. The effect of text messaging on 9- and 10-year-old children’s reading, spelling and phonological processing skills. Journal of Computer Assisted Learning 2011;27(1):28–36.

45. Yee LM, Wolf M, Mullen R, Bergeron AR, Cooper Bailey S, Levine R, Grobman WA. A randomized trial of a prenatal genetic testing interactive computerized information aid. Prenat Diagn 2014 Jun;34(6):552–7. PMID:24578289

46. Arao T, Oida Y, Maruyama C, Mutou T, Sawada S, Matsuzuki H, Nakanishi Y. Impact of lifestyle intervention on physical activity and diet of Japanese workers. Preventive Medicine 2007;45(2–3):146–152. [doi: 10.1016/j.ypmed.2007.05.004]

47. Arciuli J, Bailey B. Efficacy of ABRACADABRA literacy instruction in a school setting for children with autism spectrum disorders. Res Dev Disabil 2019 Feb;85:104–115. PMID:30530104

48. Austvoll-Dahlgren A, Bjorndal A, Odgaard-Jensen J, Helseth S. Evaluation of a web portal for improving public access to evidence-based health information and health literacy skills: a pragmatic trial. PLoS One 2012;7(5):e37715. PMID:22701531

49. Taylor-Rodgers E, Batterham PJ. Evaluation of an online psychoeducation intervention to promote mental health help seeking attitudes and intentions among young adults: randomised controlled trial. J Affect Disord 2014 Oct;168:65–71. PMID:25038293

50. Drks. Promoting Help-seeking using E-technology for Adolescents (ProHEAD). Sub-project 2: efficacy and Cost-Effectiveness of Internet-Based Selective Eating Disorder Prevention. http://www.who.int/trialsearch/Trial2.aspx?TrialID=DRKS00014679 [Internet] 2018; Available from: https://www.cochranelibrary.com/central/doi/10.1002/central/CN-01907241/full

51. Bauer S, Bilić S, Reetz C, Ozer F, Becker K, Eschenbeck H, Kaess M, Rummel-Kluge C, Salize H-J, Diestelkamp S, Moessner M, ProHEAD Consortium. Efficacy and cost-effectiveness of Internet-based selective eating disorder prevention: study protocol for a randomized controlled trial within the ProHEAD Consortium. Trials 2019 Jan 30;20(1):91. PMID:30700318

52. Blalock SJ, Solow E, Nowell WB, Woloshin S, Schwartz L, Carpenter DM, Curtis JR, Morel, L. W., Hunt C, Hickey G, Reyna V. A trial testing strategies to enhance patient understanding of drug information: Experience recruiting subjects through an online patient community. Arthritis and Rheumatology 2017;69.

53. Brettle A, Raynor M. Developing information literacy skills in pre-registration nurses: an experimental study of teaching methods. Nurse Educ Today 2013 Feb;33(2):103–9. PMID:22257584

54. Tse CK, Bridges SM, Srinivasan DP, Cheng BS. Social media in adolescent health literacy education: a pilot study. JMIR Res Protoc 2015 Mar 9;4(1):e18. PMID:25757670

55. Camerini L, Schulz PJ. Effects of functional interactivity on patients’ knowledge, empowerment, and health outcomes: an experimental model-driven evaluation of a web-based intervention. J Med Internet Res 2012 Jul 18;14(4):e105. PMID:22810046

56. Carroll JK, Tobin JN, Luque A, Farah S, ers M, Cassells A, Fine SM, Cross W, Boyd M, Holder T, et al. “Get Ready and Empowered About Treatment (GREAT) “Study: a Pragmatic Randomized Controlled Trial of Activation in Persons Living with HIV. Journal of general internal medicine 2019; PMID:31240605

57. Donker T, Bennett K, Bennett A, Mackinnon A, van Straten A, Cuijpers P, Christensen H, Griffiths KM. Internet-delivered interpersonal psychotherapy versus internet-delivered cognitive behavioral therapy for adults with depressive symptoms: randomized controlled noninferiority trial. J Med Internet Res 2013 May 13;15(5):e82. PMID:23669884

58. Isrctn. Internet-assisted therapy for adults with depressive symptoms: a study for the effectiveness of interpersonal therapy (IPT) compared to cognitive behaviour therapy (CBT). http://www.who.int/trialsearch/Trial2.aspx?TrialID=ISRCTN69603913 [Internet] 2008; Available from: https://www.cochranelibrary.com/central/doi/10.1002/central/CN-01839425/full

59. Chu AY. Psychosocial influences of computer anxiety, computer confidence, and computer self-efficacy with online health information in older adults. Texas Woman’s University; 2008.

60. Nct. Kids FACE FEARS Comparative Effectiveness Research. https://clinicaltrials.gov/show/NCT03707158 [Internet] 2018; Available from: https://www.cochranelibrary.com/central/doi/10.1002/central/CN-01664133/full

61. Nct. Web-Based Epilepsy Education Program for Adolescents and Parents. https://clinicaltrials.gov/show/NCT04144478 [Internet] 2019; Available from: https://www.cochranelibrary.com/central/doi/10.1002/central/CN-02001500/full

62. Actrn. StandingTall-plus Balance Confidence: an online cognitive behavioural therapy program to address concerns about falling in older people. http://www.who.int/trialsearch/Trial2.aspx?TrialID=ACTRN12618000926235 [Internet] 2018; Available from: https://www.cochranelibrary.com/central/doi/10.1002/central/CN-01896937/full

63. Nct. The Effect of Transitional Care Model-Based Interventions for Stroke Patients and Their Caregivers. https://clinicaltrials.gov/show/NCT03708835 [Internet] 2018; Available from: https://www.cochranelibrary.com/central/doi/10.1002/central/CN-01664181/full

64. Isrctn. LIVELIFE: effectiveness of two types of support for low mood. http://www.who.int/trialsearch/Trial2.aspx?TrialID=ISRCTN65803720 [Internet] 2008; Available from: https://www.cochranelibrary.com/central/doi/10.1002/central/CN-01811702/full

65. Friedl R, Hoppler H, Ecard K, Scholz W, Hannekum A, Ochsner W, Stracke S. Multimedia-driven teaching significantly improves students’ performance when compared with a print medium. Ann Thorac Surg 2006 May;81(5):1760–6. PMID:16631668

66. Hui A, Wong PW, Fu KW. Evaluation of an Online Campaign for Promoting Help-Seeking Attitudes for Depression Using a Facebook Advertisement: An Online Randomized Controlled Experiment. JMIR Ment Health 2015 Jan;2(1):e5. PMID:26543911

67. Nct. The Effect of a Colorectal Cancer Screening Decision Aid Tailored to Lower Educational Attainment Citizens. https://clinicaltrials.gov/show/NCT03253822 [Internet] 2017; Available from: https://www.cochranelibrary.com/central/doi/10.1002/central/CN-01495849/full

68. Geller M, Petzel S, Vogel R, Chan D, McClellan M, Gerber M, Cragg J, Jacko J, Sainfort F. A novel approach to palliative care and end-of-life decision-making: A patient-centeredwebsite to promote health care decision-making. Gynecologic Oncology 2013;130(1):e139.

69. Geller M, Petzel S, Vogel R, McClellan M, Jacko J, Cragg J, Gerber M, Chan D, Sainfort F. An interactive website for patients with ovarian cancer and their care givers-can we improve quality of life? Gynecologic Oncology 2013;130(1):e145.

70. Williams NJ, Robbins R, Rapoport D, Allegrante JP, Cohall A, Ogedgebe G, Jean-Louis G. Tailored approach to sleep health education (TASHE): study protocol for a web-based randomized controlled trial. Trials 2016 Dec 8;17(1):585. PMID:27931249

71. Goff SL, Pekow PS, White KO, Lagu T, Mazor KM, Lindenauer PK. IDEAS for a healthy baby--reducing disparities in use of publicly reported quality data: study protocol for a randomized controlled trial. Trials 2013 Aug 7;14:244. PMID:23919671

72. Actrn. Help-seeking for Depression by Young People - considering the impact of Stigma. http://www.who.int/trialsearch/Trial2.aspx?TrialID=ACTRN12609000497202 [Internet] 2009; Available from: https://www.cochranelibrary.com/central/doi/10.1002/central/CN-01836922/full

73. Actrn. Randomised controlled trial of the effectiveness of Mental Health Guru, an internet-based workplace induction program for depression and anxiety. http://www.who.int/trialsearch/Trial2.aspx?TrialID=ACTRN12613001083785 [Internet] 2013; Available from: https://www.cochranelibrary.com/central/doi/10.1002/central/CN-01835552/full

74. Nct. Effectiveness of Web-Based Health Education and Consultation on Health Promotion Behaviors of Adolescents. https://clinicaltrials.gov/show/NCT03009617 [Internet] 2016; Available from: https://www.cochranelibrary.com/central/doi/10.1002/central/CN-01561005/full

75. Parker SM, Stocks N, Nutbeam D, Thomas L, Denney-Wilson E, Zwar N, Karnon J, Lloyd J, Noakes M, Liaw ST, et al. Preventing chronic disease in patients with low health literacy using eHealth and teamwork in primary healthcare: protocol for a cluster randomised controlled trial. BMJ open 2018;8(6). PMID:29866737

76. Dirmaier J, Harter M, Weymann N. A tailored, dialogue-based health communication application for patients with chronic low back pain: study protocol of a randomised controlled trial. BMC Med Inform Decis Mak 2013 Jun 14;13:66. PMID:23768119

77. Weymann N, Harter M, Dirmaier J. A tailored, interactive health communication application for patients with type 2 diabetes: study protocol of a randomised controlled trial. BMC Med Inform Decis Mak 2013 Feb 13;13:24. PMID:23406466

78. Weymann N, Harter M, Dirmaier J. Information and decision support needs in patients with type 2 diabetes. Health Informatics J 2016 Mar;22(1):46–59. PMID:24916569

79. Heckman C, Darlow S, Munshi T, Caruso C, Ritterb, L., Raivitch S, Fleisher L, Manne S. Development of an internet intervention to address behaviors associated with skin cancer risk among young adults. Internet Interventions 2015;2(3):340–350. PMID:26640776

80. Hall LM, Ferreira M, Setchell J, French S, Kasza J, Bennell KL, Hunter D, Vicenzino B, Dickson C, Hodges P. MyBackPain-evaluation of an innovative consumer-focused website for low back pain: study protocol for a randomised controlled trial. BMJ Open 2019 May 14;9(5):e027516. PMID:31092664

81. Actrn. Efficacy of a multi-faceted web based resource on spinal health literacy in patients with low back pain - a randomised controlled trial. http://www.who.int/trialsearch/Trial2.aspx?TrialID=ACTRN12617001292369 [Internet] 2017; Available from: https://www.cochranelibrary.com/central/doi/10.1002/central/CN-01894158/full

82. Nct. Behavioral Economics-Based Online Lifestyle Balance Program. https://clinicaltrials.gov/show/NCT03534336 [Internet] 2018; Available from: https://www.cochranelibrary.com/central/doi/10.1002/central/CN-01659788/full

83. Nct. Assessing Novel Methods of Improving Patient Education of Nutrition. https://clinicaltrials.gov/show/NCT00693277 [Internet] 2008; Available from: https://www.cochranelibrary.com/central/doi/10.1002/central/CN-01587251/full

84. Jorm AF, Kitchener BA, Fischer JA, Cvetkovski S. Mental health first aid training by e-learning: a randomized controlled trial. Aust N Z J Psychiatry 2010 Dec;44(12):1072–81. PMID:21070103

85. Actrn. Can a dedicated online help-seeking website facilitate help-seeking for young adults? Pilot randomised controlled trial. http://www.who.int/trialsearch/Trial2.aspx?TrialID=ACTRN12614000386639 [Internet] 2014; Available from: https://www.cochranelibrary.com/central/doi/10.1002/central/CN-01812549/full

86. Kauer SD, Buhagiar K, Blake V, Cotton S, Sanci L. Facilitating mental health help-seeking by young adults with a dedicated online program: a feasibility study of Link. BMJ Open 2017 Jul 9;7(7):e015303. PMID:28694345

87. Feinkohl I, Flemming D, Cress U, Kimmerle J. The Impact of Personality Factors and Preceding User Comments on the Processing of Research Findings on Deep Brain Stimulation: A Randomized Controlled Experiment in a Simulated Online Forum. J Med Internet Res 2016 Mar 3;18(3):e59. PMID:26940848

88. Kiropoulos LA, Griffiths KM, Blashki G. Effects of a multilingual information website intervention on the levels of depression literacy and depression-related stigma in Greek-born and Italian-born immigrants living in Australia: a randomized controlled trial. J Med Internet Res 2011 Apr 19;13(2):e34. PMID:21504872

89. Drks. RHAPSODY: a pilot study evaluating the usability and benefits of an online learning programme for carers of people with young onset dementia. http://www.drks.de/DRKS00009891 [Internet] 2016; Available from: https://www.cochranelibrary.com/central/doi/10.1002/central/CN-01308120/full

90. Lyles CR, Tieu L, Sarkar U, Kiyoi S, Sadasivaiah S, Hoskote M, Ratanawongsa N, Schillinger D. A Randomized Trial to Train Vulnerable Primary Care Patients to Use a Patient Portal. J Am Board Fam Med 2019 Mar;32(2):248–258. PMID:30850461

91. Isrctn. A pilot research project into the use of online resources for low mood and anxiety in the voluntary sector. http://www.who.int/trialsearch/Trial2.aspx?TrialID=ISRCTN71327173 [Internet] 2007; Available from: https://www.cochranelibrary.com/central/doi/10.1002/central/CN-01869088/full

92. Ayre J, Cvejic E, Bonner C, Turner RM, Walter SD, McCaffery KJ. Accounting for health literacy and intervention preferences when reducing unhealthy snacking: protocol for an online randomised controlled trial. BMJ Open 2019 May 28;9(5):e028544. PMID:31142536

93. Actrn. Randomised Controlled Trial to reduce unhealthy snacking: evaluation of planning tools and method of allocation in a sample with diverse health literacy and diabetes or high BMI. http://www.who.int/trialsearch/Trial2.aspx?TrialID=ACTRN12618001409268 [Internet] 2018; Available from: https://www.cochranelibrary.com/central/doi/10.1002/central/CN-01908337/full

94. Ayre J, Bonner C, Cvejic E, McCaffery K. Randomized trial of planning tools to reduce unhealthy snacking: Implications for health literacy. PLoS One 2019;14(1):e0209863. PMID:30653531

95. Isrctn. Will an online life skills package be helpful for individuals with bulimia nervosa? http://www.who.int/trialsearch/Trial2.aspx?TrialID=ISRCTN41034162 [Internet] 2009; Available from: https://www.cochranelibrary.com/central/doi/10.1002/central/CN-01820055/full

96. McClay C-A, Waters L, McHale C, Schmidt U, Williams C. Online Cognitive Behavioral Therapy for Bulimic Type Disorders, Delivered in the Community by a Nonclinician: Qualitative Study. J Med Internet Res [Internet] 2013 Mar 15 [cited 2020 May 14];15(3). PMID:23502689

97. Actrn. Reel2Real: evaluation of an online social media literacy program to decrease body dissatisfaction in young adults. http://www.who.int/trialsearch/Trial2.aspx?TrialID=ACTRN12618001408279 [Internet] 2018; Available from: https://www.cochranelibrary.com/central/doi/10.1002/central/CN-01909268/full

98. Mitsuhashi T. Effects of two-week e-learning on eHealth literacy: a randomized controlled trial of Japanese Internet users. PeerJ 2018;6:e5251. PMID:30013857

99. Moll SE, Patten S, Stuart H, MacDermid JC, Kirsh B. Beyond Silence: A Randomized, Parallel-Group Trial Exploring the Impact of Workplace Mental Health Literacy Training with Healthcare Employees. Can J Psychiatry 2018 Jan 1;706743718766051. PMID:29673271

100. Isrctn. Web-based support for self-management of diabetes. http://www.who.int/trialsearch/Trial2.aspx?TrialID=ISRCTN43587048 [Internet] 2013; Available from: https://www.cochranelibrary.com/central/doi/10.1002/central/CN-01814433/full

101. Muller I, Rowsell A, Stuart B, Hayter V, Little P, Ganahl K, Müller G, Doyle G, Chang P, Lyles CR, Nutbeam D, Yardley L. Effects on Engagement and Health Literacy Outcomes of Web-Based Materials Promoting Physical Activity in People With Diabetes: An International Randomized Trial. J Med Internet Res 2017 23;19(1):e21. PMID:28115299

102. Nct. Young Men and Media Study. https://clinicaltrials.gov/show/NCT04109443 [Internet] 2019; Available from: https://www.cochranelibrary.com/central/doi/10.1002/central/CN-01992390/full

103. Nilsson U, Jaensson M, Dahlberg K, Odencrants S, Gronlund A, Hagberg L, Eriksson M. RAPP, a systematic e-assessment of postoperative recovery in patients undergoing day surgery: study protocol for a mixed-methods study design including a multicentre, two-group, parallel, single-blind randomised controlled trial and qualitative interview studies. BMJ Open 2016 Jan 13;6(1):e009901. PMID:26769788

104. Nyberg A, Tistad M, Wadell K. Effects of an internet based tool for self-management in patients with COPD-a controlled pragmatic pilot trial. European respiratory journal 2017;50:OA515. [doi: 10.1183/1393003.congress-2017.OA515]

105. Nouri SS, Damschroder LJ, Olsen MK, Gierisch JM, Fagerlin A, ers LL, McCant F, Oddone EZ. Health Coaching Has Differential Effects on Veterans with Limited Health Literacy and Numeracy: a Secondary Analysis of ACTIVATE. Journal of General Internal Medicine 2019;34(4):552–558. PMID:30756302

106. Sloan C, Stechuchak KM, Olsen MK, Oddone EZ, Damschroder LJ, Maciejewski ML. Short-Term VA Health Care Expenditures Following a Health Risk Assessment and Coaching Trial. J Gen Intern Med 2020 May;35(5):1452–1457. PMID:31898118

107. Oddone EZ, Gierisch JM, Sanders LL, Fagerlin A, Sparks J, McCant F, May C, Olsen MK, Damschroder LJ. A Coaching by Telephone Intervention on Engaging Patients to Address Modifiable Cardiovascular Risk Factors: a Randomized Controlled Trial. J Gen Intern Med 2018;33(9):1487–1494. PMID:29736750

108. Oddone EZ, Damschroder LJ, Gierisch J, Olsen M, Fagerlin A, Sanders L, Sparks J, Turner M, May C, McCant F, Curry D, White-Clark C, Juntilla K. A Coaching by Telephone Intervention for Veterans and Care Team Engagement (ACTIVATE): A study protocol for a Hybrid Type I effectiveness-implementation randomized controlled trial. Contemp Clin Trials 2017;55:1–9. PMID:28126455

109. Oenema A, Brug J, Lechner L. Web-based tailored nutrition education: results of a randomized controlled trial. Health Educ Res 2001 Dec;16(6):647–60. PMID:11780705

110. Youssef Y, Pekow PS, Lindenauer PK, Roberts JL, White KO, Mazor KM, Goff SL. How an intervention to help pregnant women use publicly available pediatric quality data affects their trust in the internet as a source for these data. Journal of General Internal Medicine 2014;29:S110–S111.

111. Actrn. Using the web to improve knowledge and self-management of Bipolar Disorder: a randomised controlled trial. http://www.who.int/trialsearch/Trial2.aspx?TrialID=ACTRN12608000411347 [Internet] 2008; Available from: https://www.cochranelibrary.com/central/doi/10.1002/central/CN-01819536/full

112. Irct2017080535509N. The effect of education on the amount of physical activity. http://www.who.int/trialsearch/Trial2.aspx?TrialID=IRCT2017080535509N1 [Internet] 2017; Available from: https://www.cochranelibrary.com/central/doi/10.1002/central/CN-01894940/full

113. Actrn. mi.spot (supportive, preventative, online and targeted): a moderated online intervention for young adults who have a parent with a mental illness and/or substance use concern: a study protocol for a randomised controlled trial. http://www.who.int/trialsearch/Trial2.aspx?TrialID=ACTRN12619000335190 [Internet] 2019; Available from: https://www.cochranelibrary.com/central/doi/10.1002/central/CN-01972700/full

114. Nct. A Study of Delivering a Mindfulness App Intervention to Accompany Supportive Care Among Women With Breast Cancer. https://clinicaltrials.gov/show/NCT02601794 [Internet] 2015; Available from: https://www.cochranelibrary.com/central/doi/10.1002/central/CN-01553617/full

115. Schwinn TM, Schinke SP, Keller B, Hopkins J. Two- and three-year follow-up from a gender-specific, web-based drug abuse prevention program for adolescent girls. Addict Behav 2019 Jun;93:86–92. PMID:30703667

116. Shensa A, Phelps-Tschang J, Miller E, Primack BA. A randomized crossover study of web-based media literacy to prevent smoking. Health Educ Res 2016 Feb;31(1):48–59. PMID:26675176

117. Isrctn. A web-based psycho-education intervention program for an adult community in Selangor, Malaysia. http://www.who.int/trialsearch/Trial2.aspx?TrialID=ISRCTN39656144 [Internet] 2015; Available from: https://www.cochranelibrary.com/central/doi/10.1002/central/CN-01801449/full

118. Kader Maideen SF, Mohd-Sidik S, Rampal L, Mukhtar F, Ibrahim N, Phang CK, Tan KA, Ahmad R. A Web-Based Psychoeducational Intervention Program for Depression and Anxiety in an Adult Community in Selangor, Malaysia: Protocol of a Randomized Controlled Trial. JMIR Res Protoc 2016 Jun 21;5(2):e112. PMID:27329333

119. Actrn. Health literacy following an educational intervention in older Australians. http://www.who.int/trialsearch/Trial2.aspx?TrialID=ACTRN12616000135415 [Internet] 2016; Available from: https://www.cochranelibrary.com/central/doi/10.1002/central/CN-01863010/full

120. Smith CA, Chang E, Gallego G, Balneaves LG. An education intervention to improve health literacy and decision making about supporting self-care among older Australians: a study protocol for a randomised controlled trial. Trials 2017 Sep 26;18(1):441. PMID:28950888

121. Smith CA, Chang E, Gallego G, Khan A, Armour M, Balneaves LG. An education intervention to improve decision making and health literacy among older Australians: a randomised controlled trial. BMC Geriatrics 2019;19(1):1–12. PMID:31064336

122. Stellamanns J, Dahal K, Schillmoeller Z. Can interactive visualizations about mammography screening improve risk knowledge and informed choice? Interim results of a randomized-controlled online experiment. Oncology Research and Treatment 2018;41:188.

123. Sullivan HW, O’Donoghue AC, Rupert DJ, Willoughby JF, Amoozegar JB, Aikin KJ. Are Disease Awareness Links on Prescription Drug Websites Misleading? A Randomized Study. J Health Commun 2016 Nov;21(11):1198–1207. PMID:27805473

124. Swallow V, Knafl K, Sanatacroce S, Hall A, Smith T, Campbell M, Webb NJ. The Online Parent Information and Support project, meeting parents’ information and support needs for home-based management of childhood chronic kidney disease: research protocol. J Adv Nurs 2012 Sep;68(9):2095–102. PMID:22224560

125. Van den Broucke S, Van der Zanden G, Chang P, Doyle G, Levin D, Pelikan J, Schillinger D, Schwarz P, Sorensen K, Yardley L, Riemenschneider H. Enhancing the effectiveness of diabetes self-management education: the diabetes literacy project. Horm Metab Res 2014 Dec;46(13):933–8. PMID:25337960

126. Nct. Decision Aid for Renal Therapy. https://clinicaltrials.gov/show/NCT03522740 [Internet] 2018; Available from: https://www.cochranelibrary.com/central/doi/10.1002/central/CN-01574352/full

127. Weinert C, Hill WG. Rural women with chronic illness: computer use and skill acquisition. Women’s health issues 2005;15(5):230‐236. PMID:16165009

128. Isrctn. Screening and support for depression in people with diabetes: a randomised controlled study. http://www.who.int/trialsearch/Trial2.aspx?TrialID=ISRCTN93089066 [Internet] 2008; Available from: https://www.cochranelibrary.com/central/doi/10.1002/central/CN-01868499/full

129. Xie B. Effects of an eHealth literacy intervention for older adults. J Med Internet Res 2011 Nov 3;13(4):e90. PMID:22052161

130. Bohingamu Mudiyanselage S, Stevens J, Watts JJ, Toscano J, Kotowicz MA, Steinfort CL, Bell J, Byrnes J, Bruce S, Carter S, Hunter C, Barrand C, Hayles R. Personalised telehealth intervention for chronic disease management: A pilot randomised controlled trial. J Telemed Telecare 2019 Jul;25(6):343–352. PMID:29793387

131. Boyne JJ, Vrijhoef HJ, Spreeuwenberg M, De Weerd G, Kragten J, Gorgels AP, investigators T. Effects of tailored telemonitoring on heart failure patients’ knowledge, self-care, self-efficacy and adherence: a randomized controlled trial. Eur J Cardiovasc Nurs 2014 Jun;13(3):243–52. PMID:23630403

132. Dinesen B, Dittmann L, Gade JD, Jorgensen CK, Hollingdal M, Leth S, Melholt C, Spindler H, Refsgaard J. “Future Patient” Telerehabilitation for Patients With Heart Failure: Protocol for a Randomized Controlled Trial. JMIR Res Protoc 2019 Sep 19;8(9):e14517. PMID:31538944

133. Fields J, Cemballi A, Michalec C, Uchida D, DeSmidt H, Cuellar J, Chodos A, Lyles CR. In-home technology training to reduce social isolation among older adults: Preliminary findings from the tech allies program. Journal of the American Geriatrics Society 2019;67:S226.

134. Schougaard LM, Mejdahl CT, Petersen KH, Jessen A, de Thurah A, Sidenius P, Lomborg K, Hjollund NH. Effect of patient-initiated versus fixed-interval telePRO-based outpatient follow-up: study protocol for a pragmatic randomised controlled study. BMC Health Serv Res 2017 Jan 26;17(1):83. PMID:28122609

135. Schougaard LMV, Mejdahl CT, Christensen J, Lomborg K, Maindal HT, de Thurah A, Hjollund NH. Patient-initiated versus fixed-interval patient-reported outcome-based follow-up in outpatients with epilepsy: a pragmatic randomized controlled trial. J Patient Rep Outcomes 2019 Sep 13;3(1):61. PMID:31520247

136. Korsbakke Emtekaer Haesum L, Ehlers L, Hejlesen OK. Interaction between functional health literacy and telehomecare: short-term effects from a randomized trial. Nursing & health sciences 2016;18(3):328‐333. PMID:26856258

137. Morony S, Weir KR, Bell KJL, Biggs J, Duncan G, Nutbeam D, McCaffery KJ. A stepped wedge cluster randomised trial of nurse-delivered Teach-Back in a consumer telehealth service. PLoS One 2018;13(10):e0206473. PMID:30379942

138. Rezvani F, Harter M, Dirmaier J. Promoting a home-based walking exercise using telephone-based health coaching and activity monitoring for patients with intermittent claudication (TeGeCoach): protocol for a randomized controlled trial. PPMP psychotherapie psychosomatik medizinische psychologie 2018;68(8):e43‐.

139. Salisbury C, O’Cathain A, Edwards L, Thomas C, Gaunt D, Hollinghurst S, Nicholl J, Large S, Yardley L, Lewis G, Foster A, Garner K, Horspool K, Man MS, Rogers A, Pope C, Dixon P, Montgomery AA. Effectiveness of an integrated telehealth service for patients with depression: a pragmatic randomised controlled trial of a complex intervention. Lancet Psychiatry 2016 Jun;3(6):515–25. PMID:27132075

140. Stockdill M, Wells R, Dionne-Odom J, Azuero A, Ejem D, Engler S, Steinhauser K, Pamboukian S, Burgio K, Durant R, Kvale E, Tucker R, Swetz K, Bakitas M. Recruitment Outcomes Among African-American and Rural Populations with Heart Failure to an Early Palliative Care Clinical Trial (S871). Journal of Pain and Symptom Management 2019;57(2):519–520.

141. Isrctn. Combining novel self-monitoring technologies for persuasive behaviour change in people at moderate or high risk of developing Type 2 diabetes. http://www.who.int/trialsearch/Trial2.aspx?TrialID=ISRCTN17545949 [Internet] 2017; Available from: https://www.cochranelibrary.com/central/doi/10.1002/central/CN-01892199/full

142. Ancker JS, Send A, Hafeez B, Osorio SN, Abramson E. Health IT Usability Focus Section: Adapting EHR-Based Medication Instructions to Comply with Plain Language Guidance-A Randomized Experiment. Appl Clin Inform 2017 Oct;8(4):1127–1143. PMID:29241250

143. Apter AJ, Bryant S, Morales KH, Wan F, Hardy S, Reed-Wells S, Dominguez M, Gonzalez R, Mak N, Nardi A, Park H, Howell JT, Localio R. Using IT to improve access, communication, and asthma in African American and Hispanic/Latino Adults: Rationale, design, and methods of a randomized controlled trial. Contemporary Clinical Trials 2015;44:119–128. PMID:26264737

144. Nct. Outcomes in CF Patients Accessing Their Registry Health Records. https://clinicaltrials.gov/show/NCT03434886 [Internet] 2018; Available from: https://www.cochranelibrary.com/central/doi/10.1002/central/CN-01523021/full

145. Pavlik V, Brown AE, Nash S, Gossey JT. Association of patient recall, satisfaction, and adherence to content of an electronic health record (EHR)-generated after visit summary: a randomized clinical trial. J Am Board Fam Med 2014 Mar;27(2):209–18. PMID:24610183

146. Friesema EM, Wagner JM, Lee JY, Lazar D, Wolf MS, Persell SD. Baseline medication reconciliation results among federally qualified health center (FQHC) patients with uncontrolled hypertension participating in the Northwestern and access community health network medication education study (NAMES). Journal of General Internal Medicine 2016;31(2):S134. PMID:16631668

147. Persell SD, Karmali KN, Lazar D, Friesema EM, Lee JY, Rademaker A, Kaiser D, Eder M, French DD, Brown T, Wolf MS. Effect of Electronic Health Record-Based Medication Support and Nurse-Led Medication Therapy Management on Hypertension and Medication Self-management: A Randomized Clinical Trial. JAMA Intern Med 2018 Aug 1;178(8):1069–1077. PMID:29987324

148. Bostrom PJ, Toren PJ, Xi H, Chow R, Truong T, Liu J, Lane K, Legere L, Chagpar A, Zlotta AR, Finelli A, Fleshner NE, Grober ED, Jewett MA. Point-of-care clinical documentation: assessment of a bladder cancer informatics tool (eCancerCareBladder): a randomized controlled study of efficacy, efficiency and user friendliness compared with standard electronic medical records. J Am Med Inform Assoc 2011 Nov;18(6):835–41. PMID:21816957

149. Duncan M, Vandelanotte C, Kolt GS, Rosenkranz RR, Caperchione CM, George ES, Ding H, Hooker C, Karunanithi M, Maeder AJ, Noakes M, Tague R, Taylor P, Viljoen P, Mummery WK. Effectiveness of a Web- and mobile phone-based intervention to promote physical activity and healthy eating in middle-aged males: Randomized controlled trial of the ManUp study. Journal of Medical Internet Research 2014;16(6):40–60. PMID:24927299

150. Duncan MJ, Vandelanotte C, Rosenkranz RR, Caperchione CM, Ding H, Ellison M, George ES, Hooker C, Karunanithi M, Kolt GS, Maeder A, Noakes M, Tague R, Taylor P, Viljoen P, Mummery WK. Effectiveness of a website and mobile phone based physical activity and nutrition intervention for middle-aged males: trial protocol and baseline findings of the ManUp Study. BMC Public Health 2012;12:656. PMID:22894747

151. Lassere MN, Baker S, Parle A, Sara A, Johnson KR. Improving quality of care and long-term health outcomes through continuity of care with the use of an electronic or paper patient-held portable health file (COMMUNICATE): study protocol for a randomized controlled trial. Trials 2015 Jun 4;16:253. PMID:26040644

152. Navaneethan SD, Jolly SE, Schold JD, Arrigain S, Nakhoul G, Konig V, Hyland J, Burrucker YK, Dann PD, Tucky BH, Sharp J, Nally JV. Pragmatic Randomized, Controlled Trial of Patient Navigators and Enhanced Personal Health Records in CKD. Clin J Am Soc Nephrol 2017 Sep 7;12(9):1418–1427. PMID:28778854

153. Nct. Sexual Health Empowerment for Women’s Health. https://clinicaltrials.gov/show/NCT03984695 [Internet] 2019; Available from: https://www.cochranelibrary.com/central/doi/10.1002/central/CN-01945499/full

154. Actrn. Using a patient-focussed electronic health system for reducing heart disease risk in people with cardiovascular disease. http://www.who.int/trialsearch/Trial2.aspx?TrialID=ACTRN12613000715774 [Internet] 2013; Available from: https://www.cochranelibrary.com/central/doi/10.1002/central/CN-01876979/full

155. Redfern J, Usherwood T, Harris MF, Rodgers A, Hayman N, Panaretto K, Chow C, Lau AY, Neubeck L, Coorey G, Hersch F, Heeley E, Patel A, Jan S, Zwar N, Peiris D. A randomised controlled trial of a consumer-focused e-health strategy for cardiovascular risk management in primary care: the Consumer Navigation of Electronic Cardiovascular Tools (CONNECT) study protocol. BMJ Open 2014 Jan 31;4(2):e004523. PMID:24486732

156. Coorey GM, Neubeck L, Usherwood T, Peiris D, Parker S, Lau AY, Chow C, Panaretto K, Harris M, Zwar N, Redfern J. Implementation of a consumer-focused eHealth intervention for people with moderate-to-high cardiovascular disease risk: protocol for a mixed-methods process evaluation. BMJ Open 2017 Jan 11;7(1):e014353. PMID:28077414

157. Nct. Regimen Education and Messaging in Diabetes (REMinD). https://clinicaltrials.gov/show/NCT03185741 [Internet] 2017; Available from: https://www.cochranelibrary.com/central/doi/10.1002/central/CN-01588605/full

158. O’Conor R, Arvanitis M, Wismer G, Opsasnick L, Sanchez Munoz A, Kannry J, Lin JJ, Kaiser D, Kwasny MJ, Persell SD, Parker R, Wood AJJ, Federman AD, Wolf MS. Rationale and design of the regimen education and messaging in diabetes (REMinD) trial. Contemp Clin Trials 2019 Aug;83:46–52. PMID:31260791
